# Systematic discovery of linear binding motifs targeting an ancient protein interaction surface on MAP kinases

**DOI:** 10.15252/msb.20156269

**Published:** 2015-11-04

**Authors:** András Zeke, Tomas Bastys, Anita Alexa, Ágnes Garai, Bálint Mészáros, Klára Kirsch, Zsuzsanna Dosztányi, Olga V Kalinina, Attila Reményi

**Affiliations:** ^1^ Lendület Protein Interaction GroupInstitute of EnzymologyResearch Center for Natural SciencesHungarian Academy of SciencesBudapestHungary; ^2^ Max Planck Institute for InformaticsSaarbrückenGermany; ^3^ Graduate School of Computer ScienceSaarland UniversitySaarbrückenGermany; ^4^ Institute of EnzymologyResearch Center for Natural SciencesHungarian Academy of SciencesBudapestHungary; ^5^ MTA‐ELTE Lendület Bioinformatics Research GroupDepartment of BiochemistryEötvös Loránd UniversityBudapestHungary

**Keywords:** cellular signaling, linear motif, MAP kinase, protein–protein interaction, Computational Biology, Signal Transduction, Structural Biology

## Abstract

Mitogen‐activated protein kinases (MAPK) are broadly used regulators of cellular signaling. However, how these enzymes can be involved in such a broad spectrum of physiological functions is not understood. Systematic discovery of MAPK networks both experimentally and *in silico* has been hindered because MAPKs bind to other proteins with low affinity and mostly in less‐characterized disordered regions. We used a structurally consistent model on kinase‐docking motif interactions to facilitate the discovery of short functional sites in the structurally flexible and functionally under‐explored part of the human proteome and applied experimental tools specifically tailored to detect low‐affinity protein–protein interactions for their validation *in vitro* and in cell‐based assays. The combined computational and experimental approach enabled the identification of many novel MAPK‐docking motifs that were elusive for other large‐scale protein–protein interaction screens. The analysis produced an extensive list of independently evolved linear binding motifs from a functionally diverse set of proteins. These all target, with characteristic binding specificity, an ancient protein interaction surface on evolutionarily related but physiologically clearly distinct three MAPKs (JNK, ERK, and p38). This inventory of human protein kinase binding sites was compared with that of other organisms to examine how kinase‐mediated partnerships evolved over time. The analysis suggests that most human MAPK‐binding motifs are surprisingly new evolutionarily inventions and newly found links highlight (previously hidden) roles of MAPKs. We propose that short MAPK‐binding stretches are created in disordered protein segments through a variety of ways and they represent a major resource for ancient signaling enzymes to acquire new regulatory roles.

## Introduction

**Figure 1:**
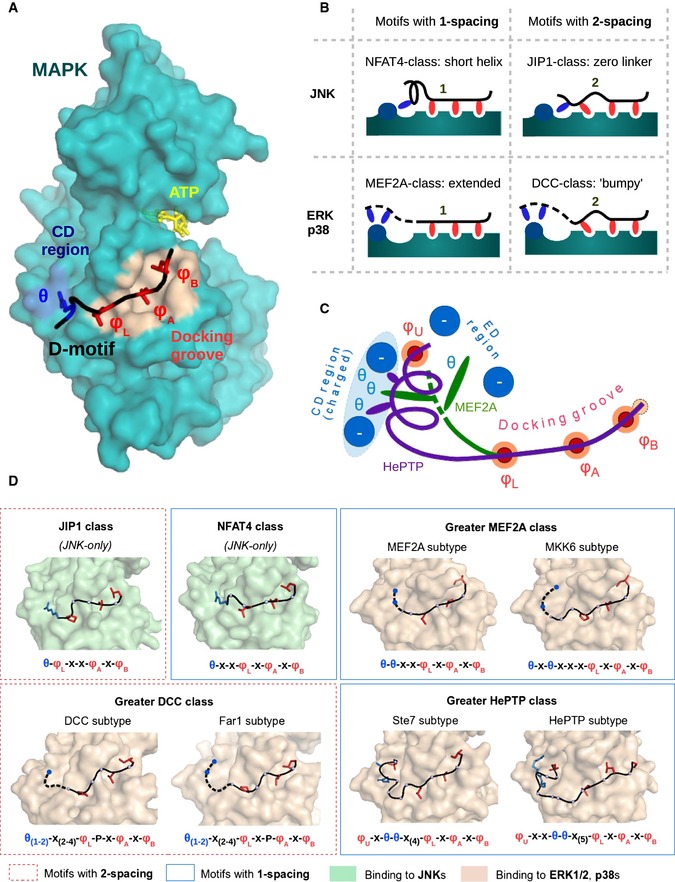
Structural classification of MAPK‐docking motifs The MAPK‐docking groove comprises two distinct regions: the common docking (CD) and the hydrophobic region. These are colored in blue and light brown, respectively, and are shown on the JNK1 surface from the JNK1‐NFAT4 protein–peptide complex crystal structure (Garai *et al*, 2012). (The CD groove is extended by the ED region, extra negatively charged residues for ERK and p38; see (C); Tanoue *et al*, 2001.)Different binding modes of D‐motifs. The hydrophobic docking groove binds three hydrophobic amino acids in a row, while admitting two different spacing schemes. At the same time, θ to φ linker length determines the MAPK specificity of a given motif. These two features can combine freely with each other, resulting in the four basic arrangements shown here.Distinct binding conformations at the CD groove. N‐termini of longer D‐motifs are variable and ERK2‐ or p38α‐binding peptides may take a variety of conformations—ranging from fully linear (e.g., MEF2A, green) to highly alpha‐helical (e.g., HePTP, magenta).Structural heterogeneity of D‐motifs. The combinations of the three variable features yield structurally well‐defined, distinct classes of D‐motifs. Many of these models also define separate types of linear motifs, but their consensus sequences are not always exclusive. JNK kinases only admit two major types of motifs, the NFAT4 class (1‐spacing, short linker) and the JIP1 class (2‐spacing, short linker). On the other hand, known ERK1/2 and p38 binder peptides may belong to the greater MEF2A class (1‐spacing, longer linker, linear end), the greater HePTP class (1‐spacing, longer linker, helical end), or the greater DCC class (2‐spacing, longer linker, linear end). A sixth class of ERK or p38 interactors is theoretically also possible (2‐spacing, longer linker, helical end), but this combination can only be observed in long reverse (revD) motifs (Garai *et al*, 2012), and no classical motif of this type is known up to date. Subtypes and other variants within a given greater class are also featured wherever applicable. These are shown based on structures of MAPK‐docking motif complexes. Dashed lines indicate N‐terminal peptide regions that are usually not visible in the crystal structures, albeit indispensable for binding. Consensus motif of each subtype is shown below, where φ_U,_ φ_L_, φ_A_, and φ_B_ letters denote positions that are filled by hydrophobic amino acids—L, A, and B refer to the lower pocket, and pockets A and B, respectively—while the θ positions are positively charged (Arg or Lys) while letter “x” denotes arbitrary amino acids.

Protein–protein interactions influence all aspects of cellular life and the most direct mechanism through which proteins can influence each other is by physical interaction. This brings them into proximity to exert control on activity or to create opportunities for posttranslational modification. Protein–protein associations often involve so‐called linear binding motifs which are short (5–20 amino acid long) protein regions lacking autonomous tertiary structure. These functional sites reside in intrinsically disordered protein regions and adopt stable conformation only upon binding. Currently, we can only guess how abundant linear motif‐based interactions are; nevertheless, it was recently estimated that there are ~100,000 linear binding motifs targeting dedicated protein surfaces in the human proteome (Tompa *et al*, 2014). As an example relevant to cellular signaling, mitogen‐activated protein kinases (MAPKs) are prototypical enzymes that depend on short segments from partner proteins and on their dedicated protein–protein interaction hot spots. They mainly recognize their substrates not with the catalytic site but with auxiliary docking surfaces on their kinase domains (Tanoue *et al*, 2000; Biondi & Nebreda, 2003). The most important of these docking sites consists of a hydrophobic docking groove and the negatively charged CD (common docking) region (Chang *et al*, 2002) (Fig 1A). Together, they can bind the so‐called D(ocking)‐motifs of the target proteins. D‐motifs are short linear motifs ranging from 7 to 18 amino acids in length and are typically found in intrinsically disordered segments—potentially far away from target phosphorylation sites (Garai *et al*, 2012). Such docking elements are not only restricted to substrates: They are also found in MAPK‐activating kinases (MAP2Ks), in MAPK‐inactivating phosphatases (MKPs), and in a variety of scaffold proteins. While extracellularly regulated kinases (ERK1‐2), c‐Jun N‐terminal kinases (JNK1‐3), and the 38‐kDa protein kinases (p38α‐δ) control diverse physiological processes, they phosphorylate most of their substrates at Ser‐Pro or Thr‐Pro (S/TP) sequence motifs. Naturally, this weak consensus provided by their catalytic site is insufficient for selective target recognition, and additional linear binding motifs provide specificity (Johnson & Lapadat, 2002; Bardwell, 2006). Therefore, the MAPK D‐motif protein–protein interaction system is an ideal test bed for linear binding motif discovery.

Several previous attempts were aimed at predicting MAPK‐binding proteins from full proteomes by using a generic consensus of D‐motifs as it had been established more than a decade ago (Sharrocks *et al*, 2000). This consensus was derived from an observation that D‐motifs almost always include at least a single positively charged residue (termed the θ position: arginine or lysine) and a series of alternating hydrophobic residues (φ positions: frequently leucine), connected by a linker of a variable length (Dinkel *et al*, 2014). But despite the use of extensive multiple alignments and sophisticated algorithms, predictions had only low success rates and large‐scale assessment of predicted hits was not performed (Whisenant *et al*, 2010; Garai *et al*, 2012; Gordon *et al*, 2013). Regarding experimental MAPK network discovery, ERK2 has been the most widely explored. For example, several different methods were utilized to identify ERK2 substrates by large‐scale phosphoproteomics (Kosako *et al*, 2009; Carlson *et al*, 2011; Courcelles *et al*, 2013). Unfortunately, pairwise overlaps between the lists of substrates are low across studies (e.g., around ~10%), with not a single overlap between five different studies that aimed to find ERK2‐phosphorylated substrates (Courcelles *et al*, 2013), suggesting great dependence on the experimental conditions used. It was noted that D‐motif‐like sequences are enriched in experimentally detected ERK2 substrates (Carlson *et al*, 2011), yet detection or verification of direct physical association was not performed. In addition, studies that used a high‐throughput approach to identify partners of JNK1 (Chen *et al*, 2014) or p38α (Bandyopadhyay *et al*, 2010) based on direct physical interaction resulted in low number of hits. Thus, it is likely that a protein–protein interaction‐based MAPK network discovery could greatly benefit from a target tailored approach, which takes into account—and possibly capitalizes on—specific biochemical and biophysical knowledge already available on known MAPK–partner protein interactions.

In recent years, the number of experimentally determined MAPK–partner protein complex structures increased considerably (Garai *et al*, 2012). This development made it possible to amend the definition of the underlying sequence motifs and it became clear that D‐motifs encompass multiple classes of similarly built, but structurally distinct linear motifs (similar to SH3‐ or PDZ‐domain‐binding sequences, for example) (Lim *et al*, 1994; Tonikian *et al*, 2008). In the current study, we show that by building a strategy that can handle this conformational diversity in binding, and using structural compatibility with specific interaction surface topography as an additional criterion for prediction, the identification of novel D‐motifs can be dramatically improved. This analysis in combination with tailored experimental techniques for the validation of low‐affinity (1–20 μM) protein–protein interactions produced unique, molecular‐level insight into physiological roles and evolution of MAPK‐based protein networks.

## Results

### Structure‐guided prediction of MAPK‐binding linear motifs

MAPK–D‐peptide complex structures revealed distinct D‐motif binding modes in the MAPK‐docking groove (Fig 1). For example, D‐motifs from the JNK‐binding scaffold protein JIP1 and from the JNK‐regulated transcription factor NFAT4 bind to the same docking surface differently (Fig 1A and B) (Heo *et al*, 2004; Garai *et al*, 2012; Laughlin *et al*, 2012). Similarly, ERK‐ and p38‐binding D‐motifs may also be structurally distinct; nonetheless, D‐motifs could be described with a common loosely defined consensus [θ_1,2_‐x_(0‐5)_‐φL‐x_(1,2)_‐φA‐x‐φB; φL, φA, and φB denote positions that are typically filled by hydrophobic amino acids—L, A, and B refer to the lower pocket, and pockets A and B, respectively—while the θ denotes positively charged (arginine or lysine) and “x” denotes any amino acid]. However, the rules are much stricter for sequences that are compatible with a given MAPK‐docking surface in a given binding mode. Interestingly, D‐motifs and their binding modes may be conserved from yeast to human as the docking surface is ancient and well conserved across all eukaryotes (Reményi *et al*, 2005; Grewal *et al*, 2006).

Because the CD region of ERK and p38 is wider compared to that of JNK, the N‐termini of motifs binding to the two former kinases have larger conformational freedom (Fig 1C) (Garai *et al*, 2012). These can be classified as MEF2A‐ and DCC‐type motifs named after the proteins in which they were first identified. Some motifs with longer intervening regions also exists (HePTP) (Zhou *et al*, 2006). Interestingly, the typical helical conformation at the N‐terminus of HePTP‐type docking motif is also characteristic to some MAPK interactors from yeast (Ste7) and peptides with such motifs are known to bind human ERK2 with high affinity (Fernandes *et al*, 2007). Therefore, we also set up a hypothetical subclass of Ste7‐type motifs, hitherto unknown in humans (Fig 1D).

**Figure 2:**
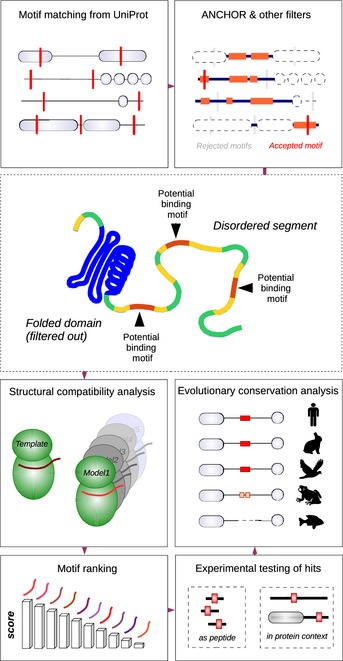
Motif finding work flow To find novel MAPK‐docking motifs, the primary motif‐matching step (on UniProt KB sequences) was followed by a series of filters. Valid motifs had to pass through an ANCHOR filter, a localization filter (combined from SignalP and Phobius) and an auxiliary Pfam filter, in order to be scored by FoldX homology models. ANCHOR (middle panel) had the most important role in selecting segments that can potentially act as linear motifs, while FoldX gave motif‐specific binding energy estimates (see Source data). Predicted motifs were subsequently tested as short fragments and (in selected cases) as full‐length proteins. Finally, an automated evolutionary analysis was performed to give information on motif conservation trends.Source data is available online for this figure.

Simple pattern matching of motifs normally produces a large number of false positives, because motif‐matching sequences may occur simply by chance. In order to drastically reduce the number of false hits, an *in silico* filtering procedure was implemented to search for putative linear motifs (Fig 2). The first step was to screen for motifs in regions with intrinsic disorder but with propensity for disorder‐to‐order transition (ANCHOR) (Dosztányi *et al*, 2009; Mészáros *et al*, 2009). This procedure was used in order to eliminate those consensus motif occurrences that would either be buried in folded domains or permanently locked in an unfavorable conformation. Importantly, it also removed initial hits with an inappropriate amino acid composition, not being able to adopt a stable structure upon binding to a protein surface. Motifs were then filtered for MAPK accessibility: Motifs that were predicted to lie in extracellular protein segments or in other, kinase‐inaccessible compartments (e.g., ER, Golgi) were discarded. In addition, an auxiliary check was performed against structured Pfam domains. This was applied to remove all spurious motifs in ordered regions which had been retained after ANCHOR filtering. Since almost all the known motifs passed these filters (45 out of 47, with enrichment ratios between 4.1 to 6.6 depending on motif type), we concluded that the dataset was of sufficient quality for further testing.

The classification of D‐motifs based on a coherent structural model enabled us to make use of structure‐based scoring. As a motif occurrence could always be unambiguously matched with its corresponding MAPK‐docking peptide structural model, we used FoldX, which had been validated on protein–peptide complexes, to estimate the change of the protein–peptide binding energy with respect to the energy of an experimentally resolved complex (Appendix Table S1) (Schymkowitz *et al*, 2005). This allowed the scoring of motifs according to their structural compatibility to the MAPK‐docking groove. FoldX‐derived binding energy estimates were also used as a guide when motifs were being chosen for later experimental screening.

### Experimental screening

**Figure 3:**
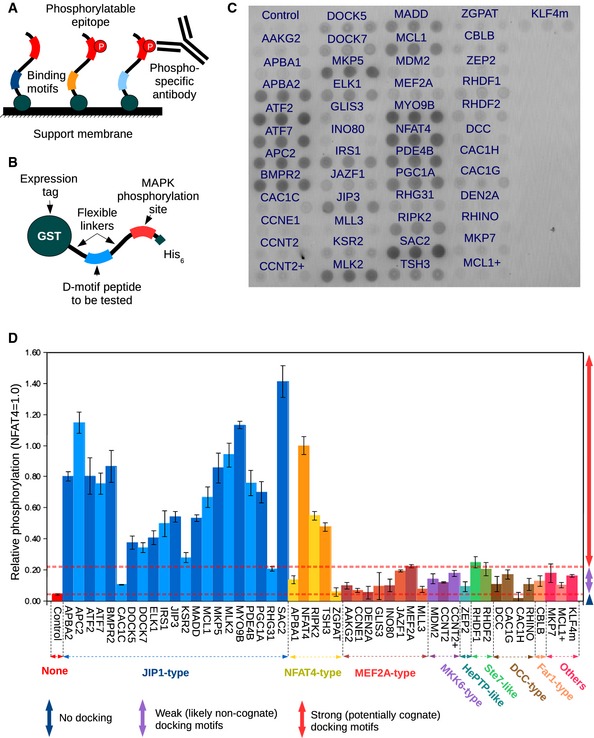
Dot‐blot arrays of D‐motifs The principle of the phosphorylation enhancement dot‐blot arrays. Protein constructs are immobilized onto a solid‐phase support where phosphorylation takes place. Afterward, the phosphorylated epitopes are detected through standard Western blot procedures using a phosphorylation‐sensitive antibody.The schematic structure of the artificial substrates utilized in the dot‐blot arrays. All constructs share the same tags, substrate sites, and linkers: Only the docking motif‐containing fragments differ.A sample dot‐blot array for detecting JNK‐binding docking motifs. This specific array contains 48 of the 70 motifs tested in total, and was incubated against activated JNK1.Quantitative analysis of the sample dot‐blot array. All intensities are relative to that of the NFAT4 motif (positive control), error bars were derived from three parallel samples on the same membrane and show standard deviation from the mean (*N* = 3). “+” denotes additional, non‐overlapping motifs tested from the same protein. “m” refers to murine (non‐human) sequence. (The corresponding ERK2 and p38α 48 motif arrays and the 70 motif arrays for all three MAPKs are shown on Appendix Fig S1 or on Fig EV1).

After completion of initial lists, we chose a number of candidate proteins from each motif type to test. Expression of full‐length human proteins of large size (> 1,000 amino acids) in recombinant systems can be a limiting step in experimental validation; therefore, first we opted for a fragment‐based approach. Former experiments showed that simple binding assays (such as pull‐downs with recombinant D‐motif‐containing proteins or immobilized solid‐phase peptide arrays) lack the robustness to reliably detect low‐affinity (1–20 μM) protein–peptide interactions. Therefore, we developed a different assay which was based on substrate phosphorylation enhancement on a solid‐phase support (Fig 3A). As the majority of known D‐motif‐containing proteins are MAPK substrates, this adequately captures the original function of these motifs. An artificial substrate was constructed containing the D‐motifs as well as the Thr71 phosphorylation site from ATF2, which is a well‐known MAPK target site (Livingstone *et al*, 1995) (Fig 3B). As linkers and substrate sites in the recombinant proteins were identical, the “docking efficiency” of the given motifs could be directly compared to each other. Phosphorylation of this reporter solely depended on the presence or absence of specific docking motifs, and phosphorylation of the target site was low without a functional D‐motif (Fig 3C and D).

**Figure EV1:**
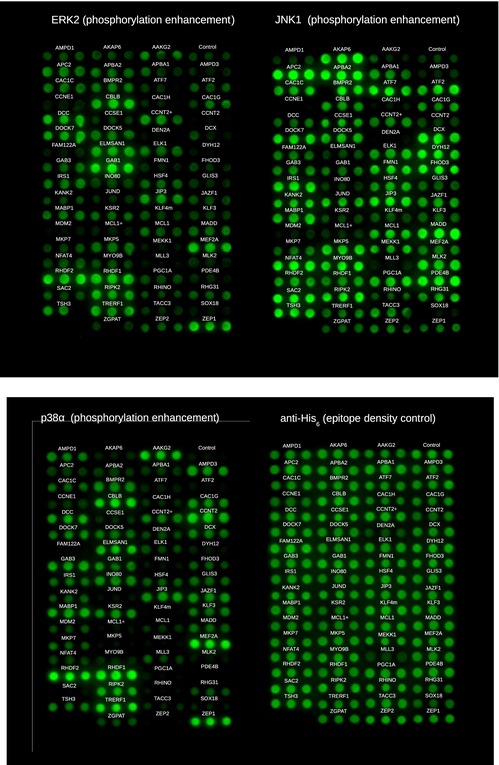
Results of dot‐blot experiments Panels show dot blots of all 71 constructs (70 D‐motif‐containing substrates + 1 negative control) against three MAPKs and the epitope density control (see alsoAppendix Fig S1).

**Figure EV2:**
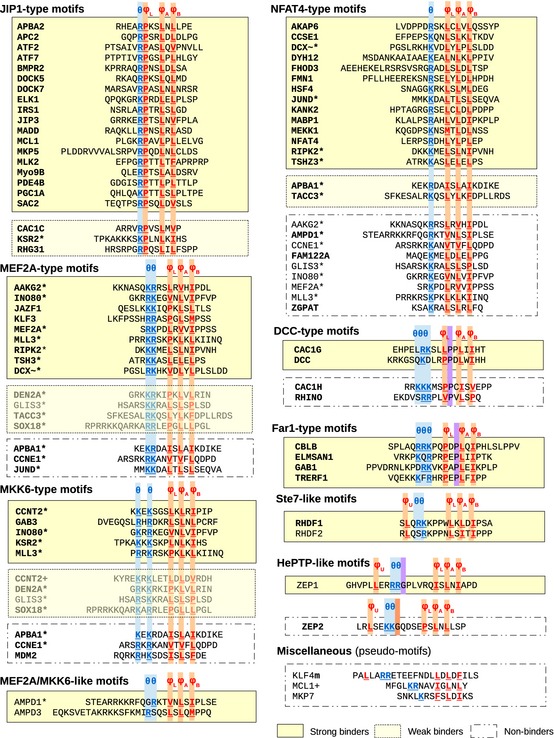
Constructs used in dot‐blot experiments and grouped according to motif class Amino acid sequences of D‐motif‐containing short segments ligated into the pAZAD vector are displayed in a tabulated format. Tested sequences are grouped according to D‐motif types. In addition, the conformity of the motif to a loose or tight motif definition (see Appendix Fig S2) is also indicated: Motifs whose sequence fits to its tight D‐motif class definition is written in bold type, while those that only correspond to a looser definition is shown in normal type. The outcome of dot‐blot experiments is also indicated: A motif is regarded as a “strong binder” (unequivocally positive) if it performed above the cutoff (non‐cognate D‐motif control) in all experiments. In case if it performed below this cutoff in at least one round of experiments, but still consistently above the level of zero control (substrate with no D‐motif), it is considered a “weak binder”. Otherwise, the motif is classified as a “non‐binder”. Only “strong binders” were considered as “positives” for the purpose of sequence and evolutionary analysis. +: These proteins had more than one, non‐overlapping MAPK‐binding elements tested (names with/without “+” refer to different sequences). *: The sequence of these constructs satisfies at least two different, overlapping consensus motifs (thus, they are featured under more than one motif class/subclass). For the sake of simplicity, not all combinations are shown. ∼: This novel isoform is found in NCBI and other databases but not in UniProt. m: This sequence refers to the murine instead of the human protein (Kim *et al*, 2012).

In the final panels, we included 70 different constructs: 63 of these were directly selected from the lists ranked by the predicted binding energy (Fig EV1 and Appendix Fig S1). We also included additional seven motifs based on sequence similarity to known motifs. This was done in order to test whether some other similar motifs not conforming to the formerly defined sequence patterns have the capacity to bind MAPKs. Out of 70, a total of 52 motifs were found to interact with at least one of the three MAPKs (ERK2, JNK1, or p38α). In particular, we were able to detect several novel interactors based on the JIP1, NFAT4, MEF2A, MKK6, and DCC models. As for our hypothetic Ste7 model, we also found a novel hit: a motif from RHDF1 that is also found in the related RHDF2 protein. Such a high number of hits suggest that D‐motifs are in fact quite widespread in the human proteome (Table 1, Fig EV2, and Appendix Fig S2).

**Table 1:** Validated sequences grouped by D‐Motif class

Greater MEF2A class (phosphorylation by p38α)			Greater DCC class (phosphorylation by ERK2)		Greater HePTP class (phosphorylation by ERK2)	
MEF2A‐type	MKK6‐type	Misc.	DCC‐type	Far1‐type	Ste7‐like	HePTP‐like
AAKG2/PRKAG2 (28–37)	CCNT2 (498–509)	AMPD1 (109–120)	**DCC (1,144–1,155)**	CBLB (489–500)	RHDF1/iRhom1 (11–24)	ZEP1/HIVEP1 (1,422–1,438)
JAZF1 (77–86)	GAB3 (363–374)	AMPD3 (79–90)	CACNA1G (1,030–1,041)	ELMSAN1 (601–612)	RHDF2/iRhom2 (18–31)	
INO80^a^ (1,318–1,327)	INO80^a^ (1,316–1,327)			TRERF1 (653–664)		
**MEF2A (268–277)**	KSR2 (330–341)			GAB1 (526–536)		
KLF3 (88–97)	KMT2C/MLL3^a^ (1,195–1,206)					
KMT2C/MLL3^a^ (1,197–1,206)						
RIPK2^a^ (326–335)						
TSHZ3^a^ (321–330)						
						
**JIP1 class (phosphorylation by JNK1)**	APBA2/MINT2 (279–285)	ATF2 (164–170)	**ATF7 (162–168)**	APC2 (962–968)	**BMPR2 (753–759)**	DOCK5 (1,762–1,768)
	DOCK7 (884–890)	DUSP10/MKP5 (18–24)	**ELK1 (314–320)**	**IRS1 (856–862)**	**JIP3 (203–209)**	M3K10/MLK2 (876–882)
	MADD (809–815)	MCL1 (136–142)	**MYO9B (1,249–1,255)**	PDE4B (72–78)	PRGC1/PPARGC1A (253–259)	SAC2/INSPP5 (1,009–1,015)
**NFAT4 class (phosphorylation by JNK1)**	**AKAP6/mAKAP (433–440)**	CCSER1 (573–580)	DYH12/DNAH12 (12–19)	FMN1 (672–679)	FHOD3 (506–513)	**JUND (46–53)**
	KANK2 (244–251)	M3K10/MEKK1 (1,077–1,084)	**MABP1 (1,292–1,299)**	**NFATC3/NFAT4 (145–152)**	RIPK2^a^ (327–334)	TSHZ3^a^ (322–329)

The table lists motifs that tested as positive (“hits”) in the dot‐blot arrays and it shows the most commonly used names of proteins (when necessary, two variants), together with the position of the core motif—based on the reference isoform featured in UniProt. Names in bold type denote previously known docking motifs, while the names in normal type indicate novel interactors.

a Denotes motifs that appear under more than one class as they satisfy multiple consensus sequences.

**Figure EV3:**
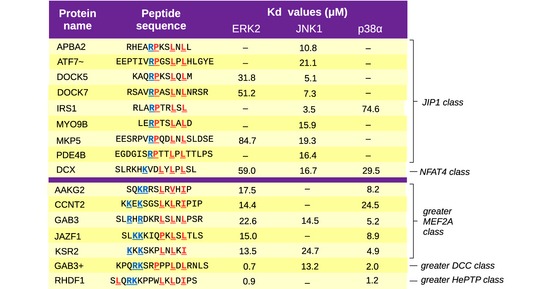
Protein–peptide binding affinity assays The results of fluorescence polarization (FP) titrations are displayed in a tabulated format, showing the sequence of synthetic peptide used together with the dissociation constants (Kd) obtained for each MAPK (see also Appendix Fig S3). Dashes indicate cases where the curves could not be fitted (the Kd is above the limit of quantitation of this assay, approximately ~100 μM). Peptides are grouped in two clusters, the first being the strong JNK1 binders (frequently with little or no ERK2/p38α‐binding ability). The second group (below the magenta line) contains peptides with strong ERK2‐ and p38α‐binding capacity (but often little or no affinity toward JNK1). Coloring of the peptide residues displays the critical charged θ residues in blue and the φ hydrophobic positions in red (according to the best fitting structural models). ~: The ATF7 peptide has non‐native flanking amino acids (several glutamic acids were added onto both its N‐ and C‐terminal ends) in order to make the peptide soluble near neutral pH. +: Motif not included in the dot‐blot screens. Gab3+ is the ortholog of a peptide from Gab1 which was tested as positive in the dot‐blot experiments. The two Gab3 motifs are related neither by structure nor by evolutionary origin.

To show that the phosphorylation enhancements were indeed due to the presence of canonical MAPK D‐motifs binding into the MAPK‐docking groove, a set of 16 chemically synthesized peptides were titrated against fluorescently labeled control peptides known to bind at the MAPK‐docking groove (Fig EV3 and Appendix Fig S3). In addition to confirming binding in the MAPK‐docking groove, the dissociation constant (Kd) of unlabeled test peptides could also be calculated.

Binding affinities obtained through this fluorescence polarization (FP) based *in vitro* assay also allowed us to examine the specificity profiles of D‐motifs. The tested peptides could be clustered into two groups based on their sequences and affinities. Similar to earlier results, these experiments confirmed the strong correlation between the ability of a given motif to bind ERK2 and p38α. Binding results also reflected the fundamental lack of correlation between ERK2/p38α and JNK1 association (Garai *et al*, 2012). These observations did agree well with phosphorylation enhancement results from dot blots. There was no positive correlation between the profiles of the JNK1 vs. p38α or the JNK1 vs. ERK2 pairs (Pearson's *r* = 0.003 and *r* = −0.280, respectively). At the same time, a modest correlation was observed between ERK2 and p38α (*r* = 0.680). This MAPK profiling confirmed our structural models. Practically, no strong JNK1‐binding motif was found from other than the JIP1‐ or NFAT4‐type classes. Most novel p38α interactors, on the other hand, belonged to the MEF2A, MKK6, or DCC types as expected.

**Figure 4:**
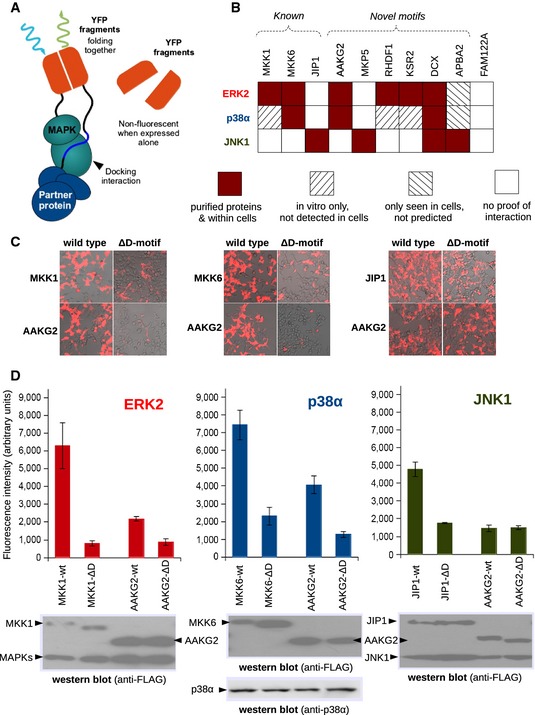
Bimolecular fragment complementation (BiFC) experiments The principle of YFP fragment complementation driven by MAPK–partner protein interaction. The weak and transient interactions between MAPKs and its binding partners still lead to well‐detectable signals.Summary of the BiFC experiments. In addition to successfully testing six novel docking motif‐dependent interactions, three positive controls (MKK1, MKK6, and JIP1) and an extra negative control (FAM122A) were also introduced into this analysis. Red squares indicate positive BiFC results (which were mostly directly predictable from fragment‐based experiments). However, some interactions suggested by dot‐blot experiments and/or FP titrations were not seen in BiFC (lined squares). These were possibly too weak or absent in the cellular context.Bright‐field image of transiently co‐transfected HEK293 cells overlaid with the fluorescence image. Although expression levels and complementation efficiency vary between cells, ablation of D‐motifs results in robust fluorescence intensity changes for known MAPK–partner protein pairs (MKK1‐ERK2, MKK6‐p38α, and JNK1‐JIP1, from left to right, upper panels) similar to a novel MAPK partner (AAKG2, lower panels).Results of fluorescence measurements on co‐transfected cell populations with positive controls and for AAKG2. (Error bars show standard deviations from the mean, *N* = 6). Similar expression levels of FLAG‐tagged proteins (wild‐type or D‐motif lacking versions of known or putative MAPK partners) or MAPKs were confirmed by Western blotting using anti‐FLAG (ERK2 and JNK1) or anti‐p38α antibodies. Further BiFC results are shown on Appendix Fig S4.

To test whether docking motifs were also functional in their native protein context, we set up a bimolecular fluorescent protein fragment complementation (BiFC)‐based cellular assay (Fig 4A). In this series of experiments, one fragment of YFP was fused to either ERK2, JNK1, or p38α. The other fragment was joined to the test protein, and fluorescence intensity was measured in live HEK293 cells. BiFC signals were always compared to the results obtained with the same construct but lacking the docking motif. Although the transgenes were overexpressed, comparison of the BiFC signal between wild‐type and D‐motif‐mutated MAPK partner proteins could be reliably used to infer D‐motif‐dependent interactions within cells.

Well‐known MAPK partners, such as MKK1, JIP1, or MKK6, displayed a pattern consistent with the specificity of their D‐motifs. Such interactions are also greatly diminished or abrogated after the loss of the docking motif, similar to novel MAPK partners (Fig 4B). For example, the MEF2A‐type motif‐bearing AMP‐activated protein kinase subunit γ2 (AAKG2) interacted with p38α (and ERK2), but not with JNK1. Interestingly, AAKG2 is known to have multiple shorter isoforms and it uses alternative initiation codons. One such variant (isoform C) is only 44 amino acids shorter. This natural deletion mutant lacking the N‐terminal (MEF2A‐type) docking motif showed a greatly reduced level of fluorescence for both partners. The differences in fluorescence were readily visible on cells under a fluorescent microscope (Fig 4C). All intensities, as well as their reduction in the mutants, were also comparable to those observed in control experiments (Fig 4D). These results are well in line with *in silico* predictions and *in vitro* fragment‐based experiments. To this end, we tested six predicted motifs (AAKG2, MKP5, RHDF1, KSR2, DCX, APBA2), and one non‐binder based on results of dot‐blot arrays (FAM122A) was also included (Fig 4B and Appendix Fig S4). Results of this cell‐based approach were consistent with the structural models as well as with the results of *in vitro* experiments.

### D‐motif‐based MAPK interactomes

**Figure 5:**
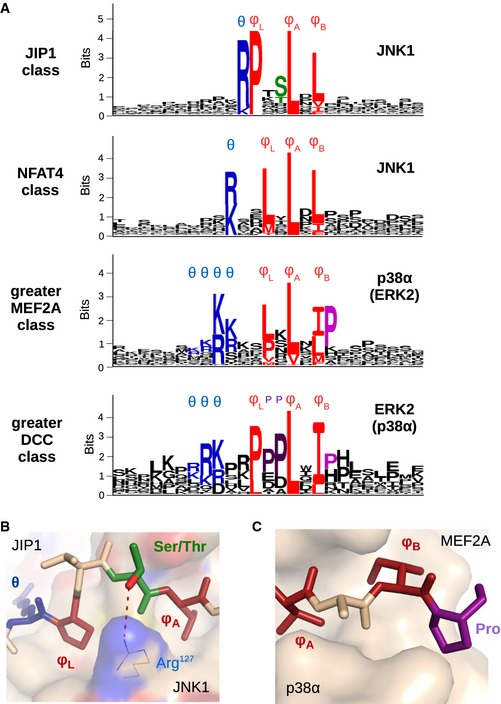
Position‐specific scoring matrix (PSSM) logos Sequence logos generated from the evolutionarily weighted PSSMs. These logos were generated for the four classes for which an adequate number of examples was available. In the core motif, the positively charged θ positions are colored blue, while the three φ hydrophobic contact points are red. The JIP1 class and the NFAT4 class were built from motifs binding to JNK1; therefore, they are directly comparable. The greater MEF2A class includes p38α‐binding motifs (with a minority also binding to ERK2). At the same time, the greater DCC class contains motifs primarily binding to ERK2 (with many of its members also associating with p38α). The logo of the greater DCC class was built based on only 6 independently evolved proteins (compared to 21 for JIP1, 15 for NFAT4, and 15 for greater MEF2A classes, respectively). As the JIP1‐type PSSM contains the highest number of independent examples, it is considered the most reliable, while the DCC is the most coarse.Positional amino acid preferences in the PSSM matrix can be explained on structural grounds even at highly variable intervening regions between core motif positions: The JIP1‐type motifs frequently have serine or threonine in the position immediately preceding φA (colored in green on the logo). From the structure of the JIP1–JNK1 complex (Heo *et al*, 2004), it is clear that this amino acid has the ability to form a hydrogen bond with the underlying arginine side chain of JNK1.The greater MEF2A—and to a lesser extent, greater DCC—motif classes show a clear preference for proline after φB (colored in light magenta). The panel on the MEF2A‐p38α complex shows that this proline can form an additional hydrophobic interaction toward the surface of p38α (Chang *et al*, 2002). Thus, the reason for this phenomenon is different from the preference for prolines in the greater DCC class (dark magenta on the logos) where the latter are required to favor a type II polyproline helix (Ma *et al*, 2010).

Next, we utilized the experimentally validated new D‐motifs to further improve our initial structural models. Evolutionary conservation analysis on motifs was also used to examine sequence conservation or diversity per each position (Fig 5A). Once the consensus sequences were improved, we set out to build a sequence‐based method to enable direct search for MAPK‐interacting proteins from the human proteome. Position‐specific scoring matrices (PSSMs) were constructed from full sets of evolutionarily related docking motifs. PSSM‐based profiles have been used in multiple databases for encoding information about sequence profiles, in the search for proteins with distant similarity, and they were recently used for detecting MAPK target phosphorylation sites (Schäffer *et al*, 2001; Hulo *et al*, 2004; Gordon *et al*, 2013).

High area under curve (AUC) values of the receiver operation characteristic (ROC) curve from a fivefold cross‐validation with validated binders and simulated non‐binders (see Materials and Methods) imply an adequate coverage of motifs in the JIP1, NFAT4, and greater MEF2A classes: 0.98, 0.94, and 0.97, respectively (Table EV1). The correlation with the original, FoldX‐based rankings was modest, but clearly present in the case of JIP1‐type (*r* = −0.62) and NFAT4‐type (*r* = −0.59) motifs (where best correlation is −1, due to the negative energy scale). It was lower for the DCC (*r* = −0.40), MEF2A (*r* = ‐0.30), and MKK6 (*r* = −0.26) models, as somewhat expected, since the structural templates of these were incomplete (as the structures of the charged N‐termini of some D‐motifs are not known). Unfortunately, the lack of sufficiently diverse hits among DCC and HePTP‐type motifs made PSSM construction impractical. A PSSM was still built for the greater DCC class, but only to compare it to the other three. This comparison showed that in several cases, positional preferences could be explained on a structural basis (Fig 5B and C).

**Figure 6:**
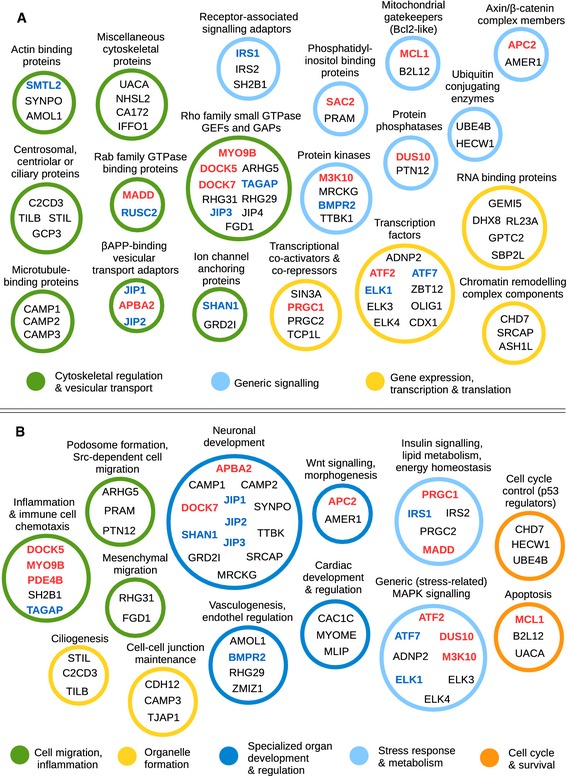
JNK interactome based on the presence of JIP1‐type motifs Low‐level functional classification of JIP1‐type motif‐bearing proteins. This analysis reveals that cytoskeletal regulation is at least as important aspect of JNK1 signaling as control of gene expression. The best 100 hits on the JIP1‐type motif list contain many proteins whose docking motif is already known (blue letters), or was validated in our dot‐blot essays (red letters). Only categories that contain more than a single protein are shown.High‐level functional classification of the best‐ranking 100 JIP1‐type motif. The analysis reveals several major functions associated with JNK kinases. The role of JNK in inflammation, neuronal development, metabolic regulation, or apoptosis is already known from cell‐based experiments. Motif search results, in addition to providing the mechanistic basis for these regulatory processes, also suggest novel functions. The full functional clustering of JIP1‐type D‐motifs along with NFAT4‐ and MEF2A‐type motifs is shown in Table EV2.

The structurally consistent PSSM‐based search method offered us a unique glimpse into the human MAPK interactome, albeit limited to D‐motif‐containing proteins. As it included a rather large number of proteins that have little or no formal Gene Ontology (GO) annotation, we decided to annotate the best 100 hits manually, based on UniProt labels, domain composition, and literature (Table EV2). Out of the three classes examined, the JIP1 type had by far the highest number of validated hits. Thus, the predictions for this class were deemed most reliable, shedding some light on the interactome of JNK1 (Fig 6). Among the less surprising categories discovered were the MAPK pathway components themselves (especially at the MAPK kinase kinase [MAP3K] level, as potential feedback elements), several transcription factors and other gene expression regulatory systems, or various ubiquitin ligases. A considerable number of experimentally tested or predicted JNK‐interacting proteins have preferentially or exclusively neuronal functions. We predict that the axons, nerve terminals, and dendrites—especially in synapses—contain a high number of specialized JNK‐interacting proteins, as do developing neuroblasts and their axonal growth cones (Appendix Fig S5A). Interestingly, the majority of JNK‐associating proteins (both experimentally validated and predicted) seem to be involved in cytoskeletal regulation. We encountered numerous actin‐binding or microtubule‐binding proteins, molecular motors as well as small G protein partners. Docking motifs were even found on proteins localized to centrosomes, basal bodies, or those involved in the formation of primary cilia. Several other high‐scoring hits suggest that JNK is intimately involved in the regulation of endo‐ and exocytosis.

The presence of insulin signaling pathway components in the lists may also explain many previous observations on the causative role of JNK in type II diabetes. This kinase is involved in pathways over‐activated by cytokines derived from adipose tissue. JNK1 knockout mice are also known to be resistant to type II diabetes induced by obesity (Hirosumi *et al*, 2002; Sabio *et al*, 2010). Proteins bearing JIP1‐type docking motifs (e.g., MADD, IRS1, PGC1A) are located in critical points of networks responsible for insulin signaling, and these are the same pathways that are also targeted by most anti‐diabetic pharmaceuticals (Lee *et al*, 2003; Finck & Kelly, 2006; Olson *et al*, 2008; Li *et al*, 2014) (Appendix Fig S5B).

Experiments with greater MEF2A‐type motif‐bearing proteins provided another interesting observation. The occurrence of such motifs in proteins (KSR2, AAKG2, and AMPD1,3) with an important regulatory role in the adenosine monophosphate‐activated protein kinase (AMPK) pathway implies that this system connects to the p38 and/or the ERK1/2 pathways on very specific points in specialized tissues (Costanzo‐Garvey *et al*, 2009; Pearce *et al*, 2013; Rybakowska *et al*, 2014) (Appendix Fig S5C). Interaction with tissue‐specific protein isoforms may thus cause highly cell‐type‐specific regulation by MAPKs.

The analysis of the best 100 hits for the NFAT4 class yielded results similar to the JIP1‐type motifs, with some differences. In contrast, members of the greater MEF2A class were markedly dissimilar from those of the JIP1 class. Here, the proportion of cytoskeletal proteins was minimal, while the fraction of transcription factors was considerably higher. Proteins involved in other functions related to gene expression, such as chromatin remodeling or histone methylation, were also present in higher numbers. When comparing distributions of protein functions, the NFAT4 class appeared to lie between the two extremes represented by the JIP1 types (mostly cytoplasmatic targets) and greater MEF2A types (emphasizing nuclear actions) (Appendix Fig S6A). The similarity of NFAT4‐type motif‐containing proteins to JIP1‐type bearing ones is easy to understand: Both primarily interact with JNK1. In certain protein families, one can discover closely related pairs in which one protein contains a JIP1‐type docking motif and the other contains a likely independently evolved NFAT4‐type docking motif (Appendix Fig S6B). On the other hand, the NFAT4‐type motifs are structurally compatible with MEF2A types (unlike JIP1 types); thus, some of the predicted best binders are shared between the latter two lists. Our dot‐blot experiments indeed corroborated that the overlapping motif definitions result in a naturally overlapping set of interactors for JNK1 and p38α (Appendix Fig S6C).

### Evolutionary analysis of D‐motifs

**Figure 7:**
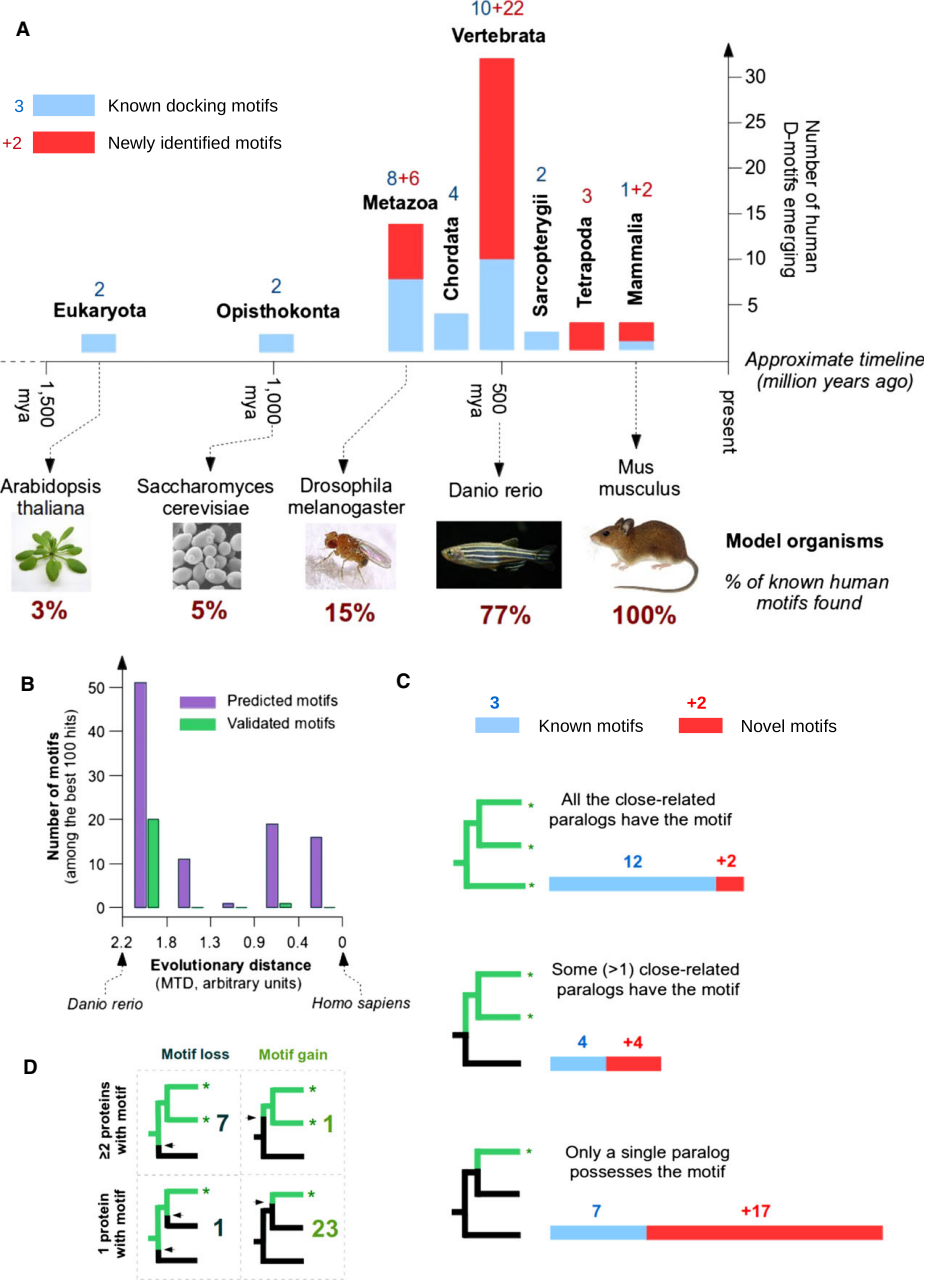
Evolutionary analysis of D‐motifs Analysis of MAPK‐docking motif emergence (based on p‐BLAST searches) paints a dynamic picture of MAPK pathway evolution. The panel was made based on 62 independent MAPK D‐motif occurrences (see Table EV3). The histogram counts the number of known D‐motifs (blue bars and blue numbers) and those newly identified in our experiments (red bars and red numbers). An approximate timeline is also added to give a realistic scale of the time dimension. The percentage of mammalian motifs found in selected model organisms is also indicated. Among model organisms, mice and zebrafish are relatively similar to human based on their MAPK interactomes. But fruit flies or yeast are rather poor models due to the low number of docking motifs being conserved across species.Distribution of the 100 best JIP1‐type motifs (predicted by the PSSM) versus their eggNOG‐derived maximum traceable distance. Motifs validated experimentally as binders are represented under the green columns, while the total number of predicted motifs is shown in magenta. The analysis suggests that the most recently emerged motifs are still under‐explored. While a reasonable percentage of D‐motifs shared between humans and zebrafish were successfully validated in experiments, there also appears to be an intriguing number of (predicted) motifs restricted to mammals only. Note that this distance metric is different from the one used in (A) and extends to bony fishes only.The branching pattern of closely related human proteins with MAPK‐docking motifs points at rapid evolution. Most of the already‐established MAPK partner proteins (blue bars) are members of families where more than one paralog carries the same motif. However, the more recently identified docking motifs (red bars) show a rather different picture. Proteins with stand‐alone docking motifs thus appear to be much more common than previously expected.Comparison of vertebrate and invertebrate genomes suggests that most novel D‐motifs may provide paralog‐specific regulation. The panel traces the emergence of D‐motifs within protein families. Where multiple vertebrate paralogs carry the same motif, the docking elements are overwhelmingly pre‐vertebrate inventions (often subject to motif loss, upper rows). However, where only a single paralog has the motif, the trend is exactly the opposite: Most motifs have typically newly evolved and have no counterpart in invertebrates (lower rows).Data information: * denotes human paralogs containing validated D‐motifs in (C) and (D).

MAPK pathways are found in almost every eukaryotic organism, and the three‐tiered kinase cascade architecture of the MAPK module core is well conserved from yeast to human. Therefore, one would naturally expect the downstream targets of these pathways to be conserved as well. However, our results do not support this and in fact suggest the opposite. Although evolutionary conservation is considered to be a predictive feature of a functional linear motif, this did hold through for D‐motifs. There was no correlation between FoldX (predicted binding energy) and any of the evolutionary conservation scores. The maximum traceable distance (MTD) of a motif in evolutionarily related species could be calculated from the eggNOG alignments. Here, we also noted that most of the motifs were traceable to vertebrates only. A more thorough search, using p‐Blast searches in the UniProt database, revealed that some motifs are actually more ancient than what eggNOG data would suggest. Still, a high number of experimentally validated motifs were found to be relatively recent evolutionary inventions. After mapping the most distant organisms in which the motif in question is already present, we were able to compile an evolutionary histogram of MAPK‐docking motif emergence (Fig 7A and Table EV3). Despite the fact that MAPK pathways are an eukaryotic common heritage, very few human docking motifs had an ancestry among unicellular organisms. This latter was only true for the MAPK kinases (MAP2Ks) or MAPK‐activated kinases (MAPKAPKs) and a few truly ancient substrates, like MEF2/MADS‐box proteins. Only in multicellular animals (Metazoa) did docking motifs become detectable in a variety of phosphatases and MAP3Ks as well as in the core set of mammalian substrates (ELKs, ATFs, JUNs, etc.). However, some of these motifs were difficult to find as they were subsequently lost in several lineages, especially in arthropods. The diversification of docking motifs continued in chordates, but it was in early vertebrates where a major re‐wiring and expansion of MAPK partnerships occurred. Over 50% of the motifs identified in our experiments evolved at this period. After the development of bony fishes, motif emergence events became less common, but did not stop completely: New motifs appeared in lobe‐finned fishes (Sarcopterygii), in terrestrial vertebrates (Tetrapoda), and even in mammals. Comparison of the known and predicted motifs from the best 100 hits for JIP1‐type motifs suggests that there are many more recently evolved motifs in mammals (Fig 7B). These findings are well in line with recent results on yeast calcineurin interactomes: Yeast phosphatase‐docking motifs were found to evolve fast, and their distribution was highly divergent between related species (Goldman *et al*, 2014).

A further proof for the late evolution of MAPK partnerships is found when comparing paralogs (Fig 7C). These latter are closely related copies of the same ancestral gene that often preserved linear motifs from before their split. Most vertebrate proteins come in groups of 2, 3, or 4 closely related paralogs due to twin genome duplications—and subsequent gene loss—at the dawn of vertebrate evolution (Ohno, 1993; Durand, 2003). Interestingly, most of the better‐known MAPK target proteins possess a D‐motif in more than one vertebrate paralogs. However, the same is not true for the majority of novel partner proteins. Comparison of vertebrate proteins with those from earlier‐branching genomes also helped us to determine whether a motif developed after the gene duplications or before (Fig 7D). Our analysis suggests that the presence of a motif in more than one paralog is predictive for ancient motif emergence. (Over 50% of such protein families have non‐vertebrate members with the motif already in place.) In this case, motif loss appeared to be the dominant mechanism to create differences between vertebrate paralogs. Only a very few new motifs emerged in‐between the two whole‐genome duplication events, suggesting that this evolutionary stage was short‐lived (Kuraku *et al*, 2009)⁠. On the other hand, where only a single paralog contained the motif, this motif was predominantly a new invention after the twin duplications—and not a result of an ancient motif being lost. This was the most common scenario for newly found D‐motifs. Many of these novel MAPK‐recruiting motifs are suspected to provide a paralog (or even isoform)‐specific regulation, thereby offering unique roles to otherwise highly similar human proteins.

**Figure 8:**
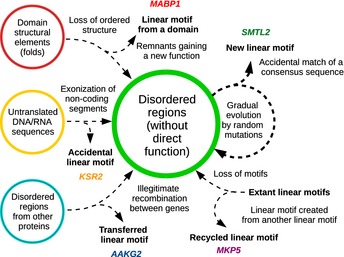
Mechanisms of binding motif emergence D‐motifs have diverse evolutionary origins. They most commonly originate from disordered protein segments via gradual accumulation of point mutations (e.g., SMTL2). However, they may also be created from folded domains (e.g., MABP1), or being introduced entirely *de novo*, from a previously untranslated genomic segment (e.g., KSR2). D‐motifs can also be born from existing linear motifs, through gene fusion or recombination (e.g., AAKG2). But more commonly, this involves transmutation of a (previous) linear motif into a new one (example: MKP5). For more details, see Table EV3 and Appendix Fig S7.

Having obtained a sufficient number of experimentally verified examples, we could also test some theories on the evolutionary processes creating the linear motifs. The motifs we validated (at least at a fragment level) could be classified based on their predicted origins (Fig 8 and Table EV3). Not surprisingly, the most common way of motif emergence appeared to be random mutations in an already‐existing disordered segment. This can be illustrated by a known interactor, the Smoothelin‐like protein 2 (SMTL2) (Gordon *et al*, 2013). Here, gradual sequence changes in terrestrial vertebrates led to the creation of the motif, which is restricted to placental mammals (Eutheria) (Appendix Fig S7A). There were also several examples for creation from scratch (i.e., from non‐coding DNA). This could mean either translational start shift (translating an earlier non‐translated 5′ UTR) or splicing site shift (leading to exonization of intronic sequences). While the N‐terminal expansion of the protein is seen in MCL1 (as the motif‐bearing segment has no counterpart in Bcl2 or in any other related protein), another newly identified partner, KSR2, serves as an example for splicing site rearrangements (Appendix Fig S7B). Here, the paralog KSR1 retains the ancestral intron–exon boundaries, which appear to have shifted in KSR2 (Appendix Fig S7C). We could even find examples for proteins where this mechanism may still be active: The motif can (in an isoform‐specific way) be included or excluded due to alternative splicing or initiation. This is the case with the PDE4 genes, where most paralogs (PDE4A, PDE4B, PDE4D) still retain an ancestral, alternative exon containing a JIP1‐type motif (Appendix Fig S7D).

Interestingly, linear motifs can also transmute into each other: Some examples in the dataset show potential switching between different MAPK‐docking motif classes. As a result, distant organisms may show different motif types at the same location: That is, the JIP1‐type motif that we identified in MKP5 corresponds to an NFAT4‐type motif in distant organisms (as in arthropods). In contrast, CCSER1 has an NFAT4‐type motif in humans, but a JIP1‐type one in zebrafish (Appendix Fig S7E). The motif in ELK1 is of the JIP1 type in humans, but Far1 type in protostomes. Also, the motif of TAB 1 is DCC‐like in most non‐vertebrate organisms, unlike the human which is MEF2A‐like (Appendix Fig S7F). In some cases, a “horizontal motif transfer” (i.e., recombination between unrelated genes) may have complemented *de novo* emergence of motifs. This was the likely case for AAKG2, and the N‐terminal region of AAKG2 (which is unique and sets it apart from AAKG1) showed a surprising similarity to the C‐termini of MEF2A or MEF2C. In addition to the core motif, the disordered segments flanking the motifs also aligned well, and this cannot be explained by convergent evolution alone as the latter regions are not subject to the same selection (Appendix Fig S7G). The creation of a new linear motif from the unfolded remnants of earlier structured domains was yet another intriguing possibility. For the WDR62/MABP1 family, the duplication of WD40 repeats and their subsequent degeneration were the most likely source of the NFAT4‐like D‐motif (Appendix Fig S7H).

### Functional aspects of docking motif evolution

**Figure EV4:**
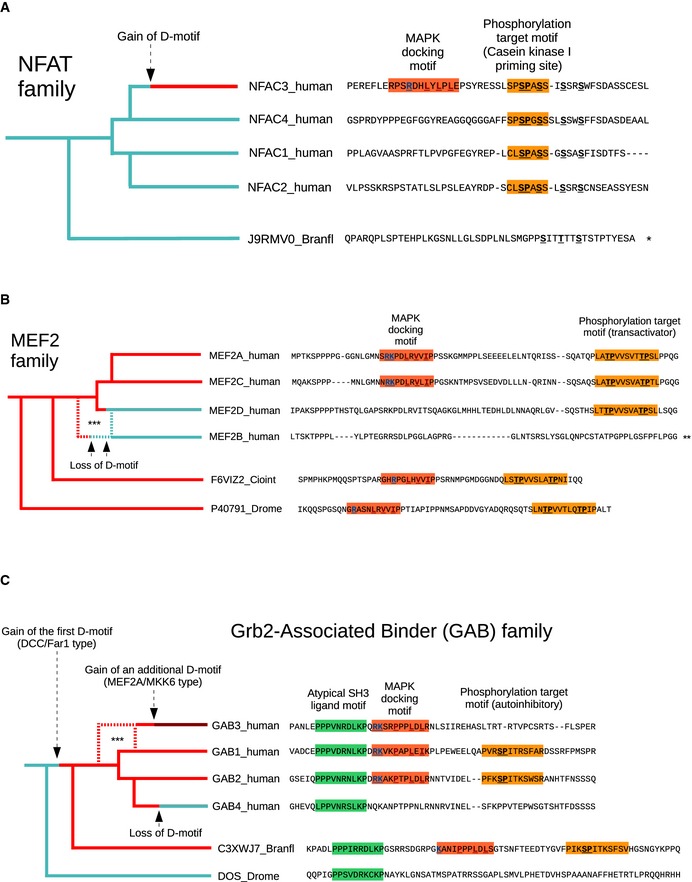
Coevolution of MAPK‐docking motifs and phosphorylation target sites In the case of the NFAT family of transcription factors, phosphorylation of an N‐terminal regulatory motif (the so‐called serine‐rich region 1/SRR1) serves to retain the protein in the cytoplasm, denying nuclear entry. While processive phosphorylation of the SRR1 motif is performed by casein kinase I (CK1) in all four vertebrate paralogs, priming of phosphorylation can be provided by diverse other protein kinases. In the case of NFAT4 (also known as NFATC3), a JNK‐specific docking motif ensures that CK1 priming can be driven by JNK kinases (Chow *et al*, 1997). Comparison with other NFAT paralogs suggests that this mechanism in NFAT4 is fairly novel, adding on to a previously evolved regulatory system. (Some elements of the SRR1 motif can already be recognized in non‐vertebrate orthologs).The docking motif encountered in the human myocyte enhancement factor 2 A (MEF2A) protein is ancient: Transcription factors belonging to the MEF2/MADS‐box family carry docking motifs in almost all multicellular animals (and even in some unicellular eukaryotes, fungi, or plants) (Jung *et al*, 2002; Yang *et al*, 1999). The D‐motif controls phosphorylation of a conserved motif indispensable for transactivation. However, this ancestral module is preserved in two of the four vertebrate paralogs only (MEF2A and MEF2C). Other vertebrate paralogs show either a partial loss of the docking motif (MEF2D) or a complete loss of the docking motif together with the transactivator motif (MEF2B). The latter is probably an evidence of neofunctionalization, with the divergent MEF2B no longer being involved in generic mesoderm and myocyte development, taking up other specialized roles (such as lymphocyte regulation) instead (Ying *et al*, 2013; Estrella *et al*, 2015).The Far1‐type MAPK‐docking motif identified by our experiments in GAB1 has homologs in 3 out of the 4 human GAB proteins. It is located in the vicinity of a well‐known ERK2 phosphorylation site that controls an intramolecular interaction. Residues surrounding this C‐terminal site bind to the N‐terminal plecstrin homology (PH) domain preventing membrane recruitment of GAB proteins (Eulenfeld & Schaper, 2009). Phosphorylation by ERK2 disrupts the intramolecular interaction allowing GAB1 translocation (Wolf *et al*, 2015). Although testing this hypothesis requires further experiments, it is probable that the ERK2‐docking motif and the ERK2 phosphorylation site form a single functional module. The evolutionary analysis suggests that both sites emerged prior to the development of vertebrates, at roughly the same time (the entire module is found in a single *Branchiostioma floridae* protein, but not in other invertebrates). While GAB1 and GAB2 retained the ancestral architecture, GAB4 lost both the docking motif and the phosphorylation target site. GAB3 did lose the phosphorylation target site, yet the D‐motif was retained. However, emergence of an additional, MEF2A‐type motif in GAB3 further away from the original one suggests that these motifs might now control different target site(s) in GAB3. Data information: *, Match between the regions is insecure due to poor conservation; **, the sequence is so divergent from the other vertebrate paralogs that the remnants of the original MAPK‐regulated region could only be matched to the rest with the help of intron–exon architecture (MEF2B); ***, The exact branching pattern is uncertain, due to high divergence of certain paralogs (MEF2B or GAB3).

The typical purpose of docking motifs is to enable phosphorylation of recruited substrates (Reményi *et al*, 2006). Indeed, when the newly identified D‐motifs from AAKG2 and DCX were mutated, their phosphorylation by their cognate MAPK was greatly reduced (Appendix Fig S8). Due to the lack of strict spatial constraints between D‐motifs and phosphorylation sites, such roles can only be interpreted in the broader context of a protein. Unfortunately, most phosphorylation target sites controlled by the novel docking motifs remain elusive. Yet in some cases, such sites either have been discovered beforehand or could be inferred based on spatial proximity and/or coevolution with D‐motifs (Gordon *et al*, 2013). Examples for the latter give insight into how docking motifs emerged in relation to their target sites. In particular, both motif loss and gain are expected to have a profound effect on target sites: potentially endowing the protein with a new regulation. This is well illustrated by three cases: the nuclear factor of activated T cells (NFAT) family, with a *de novo* motif created in NFAT4 (adding on to a preexisting target site, Fig EV4A); the myocyte enhancer factor 2 (MEF2) family, displaying motif loss to a varying degree (with the concomitant loss of target sites, Fig EV4B); and the Grb2‐associated binder (GAB) family, in which both events took place (Fig EV4C) (Chow *et al*, 1997; Yang *et al*, 1999; Wolf *et al*, 2015). To examine the evolutionary relationship between MAPK‐binding D‐motifs and phosphorylation target sites, the presence of validated D‐motifs was analyzed in parallel with experimentally validated S/TP phosphorylation sites (Table EV4). This analysis was carried out in 50 proteins and compared the evolutionary conservation depth of the human motifs among vertebrate homologs (Appendix Fig S9). We found that there is a correlation in conservation of D‐motifs and the most conserved putative phosphorylation sites, suggesting that D‐motifs and putative target sites may have coevolved.

Our studies also support the notion that there are a number of proteins with multiple MAPK‐docking elements. Apart from the case where these elements interact with different MAPKs, such as in the case of MKP5, where a rhodanese domain can bind p38 and a JIP1‐type linear motif interacts with JNK, or GAB3, with separate motifs to recruit ERK1/2 or p38, the purpose of multiple D‐motifs is unclear, especially because they tend to bind to the same surface and thus compete with each other for MAPK binding. Such domains or motifs are often not simple duplicates of each other and emerged independently during evolution. This happened in the case of BMPR2, where the first JIP1‐type motif is found in almost all multicellular animals, but the second one is restricted to vertebrates (Podkowa *et al*, 2010) (Appendix Fig S10A). In addition, the ATF2/CREB family of transcription factors has an N‐terminal motif next to a Zn‐finger serving as the primary docking element in all metazoans, but several vertebrate paralogs have an additional JIP1‐type motif with an unclear role (Appendix Fig S10B). As one MAPK molecule can only accommodate one motif at a time, it is probable that multiple docking motifs would allow several, mutually largely exclusive complexes—each with unique spatial orientation. As in the case of MKK7 which activates JNK1, the precise orientation of the MAPK versus the partner protein might have important implications on phosphorylating specific target sites (Ho *et al*, 2006; Kragelj *et al*, 2015).

D‐motifs in proteins are known to facilitate phosphorylation of MAPK targets. However, the functional consequences of MAPK target site phosphorylation are unfortunately often not well understood. Phosphorylation may have diverse impact on protein function, and its relevance may only be revealed in the context of a signaling cascade. To this end, we have characterized the role of one of the newly found D‐motif (527‐RKVKPAPLEI‐536) in the GAB1 signaling adapter protein (see Table 1 or Fig EV2, Far1‐type) in HEK293 cell‐based assays. GAB1 belongs to the insulin receptor substrate 1 (IRS1) family of adapter/scaffolding molecules playing a role in multiple signaling pathways (Holgado‐Madruga *et al*, 1996). Recently, it has been shown that cell membrane recruitment of GAB1 via its PH domain is controlled by an intramolecular switch (Wolf *et al*, 2015). The PIP3‐binding surface of the GAB1 PH domain is masked by intramolecular interactions and phosphorylation at Ser551 unmasks this membrane binding surface and promotes recruitment of GAB1 to the cell membrane (Appendix Fig S11A). Interestingly, Ser551 in human GAB1 is a known MAPK S/TP target site, and we tested whether the newly found D‐motif in this scaffold protein is indeed important for GAB1 membrane recruitment (Appendix Fig S11B), and whether it influences EGF/Ras→ERK2 signaling (Appendix Fig S11C). We found that docking motif versions of GAB1 had diminished capacity to translocate to the cell membrane and that these mutants were more sensitive to EGF stimulation regarding ERK2 activation. These results are fully consistent with GAB1's role as a complex regulator of EGF‐mediated signaling: It exerts negative feedback control on the EGF/Ras→ERK2 pathway, presumably by relying on its ERK2‐binding D‐motif.

## Discussion

Protein kinases often use dedicated domains for substrate recognition. Known examples include the Src‐family tyrosine kinases (SH2 and SH3 domains) (Alexandropoulos & Baltimore, 1996; Pellicena *et al*, 1998), SPAK/OSR kinases (unique domain) (Vitari *et al*, 2006) and Polo‐like kinases (Polo‐box domains) (Lee *et al*, 2014). In other cases, recruitment is provided by the catalytic domain itself, but by a distinct surface which is noncontiguous with the catalytic site. This appears to be common among relatives of MAPKs, the so‐called CMGC (cyclin‐dependent kinase/MAPK/glycogen synthase kinase 3/CDK‐like) kinase group. However, each kinase family uses a different surface, with strikingly different recognition modes. Thus, motifs recognized by GSK3 or SRPK kinases (Dajani *et al*, 2003; Ngo *et al*, 2005) are unrelated to D‐motifs or FxFP‐motifs of MAPKs, or to CDK‐docking motifs recognized by the cyclin subunit, for example (Lowe *et al*, 2002). Based on our results on MAPK‐binding D‐motifs, it may be anticipated that insights into other recruitment motif‐based systems will greatly contribute to a system‐level understanding of protein kinase‐based intracellular signaling networks.

In the current study, it was demonstrated that canonical, D‐motif‐dependent partners of MAPKs are in fact quite common. However, a number of partners with atypical or “naturally defective” docking motifs do exist (e.g., MKK3, MKK7, TAB 1), and these are difficult to predict (Chang *et al*, 2002; De Nicola *et al*, 2013). Often such defective motifs act in a non‐autonomous way: These weak elements may be complemented by additional protein stretches, motifs, or domains (Glatz *et al*, 2013). Besides, not all MAPK‐binding elements are linear motifs. Folded domains such as the rhodanese domain of dual‐specificity phosphatases may bind to the same site as intrinsically disordered docking motifs (Zhang *et al*, 2011). It should be noted that motifs other than the canonical D‐motifs (e.g., the so‐called FxFP‐motifs) also exist (Jacobs *et al*, 1999; Fantz *et al*, 2001; Zhang *et al*, 2003). A considerable number of interactions might also be indirect, mediated by a third partner. Nevertheless, directly interacting with a MAPK solely through short linear motifs appears to be a major and widespread phenomenon in mammals. Although experimental testing of all putative MAPK D‐motifs could not be performed, we suggest that the fraction of the human proteome that harbors high‐scoring D‐motifs may be representative of the full interactomes for three distinct MAPKs, which may be best captured for JNK1 by the procedure presented in this study.

**Figure EV5:**
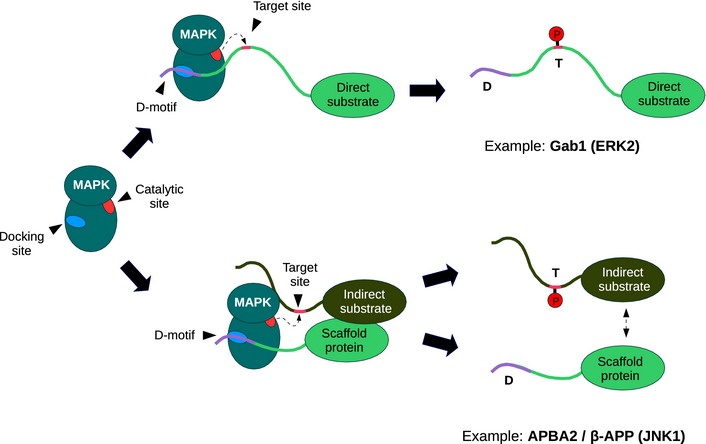
Illustration of direct and indirect MAPK substrates Most of the D‐motif‐containing proteins likely fall into two categories. In proteins that are direct MAPK substrates (upper diagram), the docking motif might serve to enhance phosphorylation of regulatory sites. This is exemplified by a known substrate, GAB1 (with the target motif being only recently identified) (Wolf *et al*, 2015). Other proteins can act as scaffolds to aid phosphorylation of substrates without docking motifs (here termed indirect MAPK substrates, lower diagram). For example, the requirement for APBA2 to mediate JNK‐dependent phosphorylation of beta‐amyloid precursor protein (β‐APP) has already been described and the minimally required fragment of APBA2 contains the JIP1‐type docking motif‐containing region as well as a PTB domain (Taru & Suzuki, 2004).

Some of the newly identified partners directly fit into the core of MAPK pathways. These include specific phosphatases as well as MAPK kinase kinases (MAP3Ks). While there can be little doubt that docking motifs of phosphatases would be required for MAPK dephosphorylation, the presence of docking motifs in MAP3Ks is a more intriguing observation. It is probable that phosphorylation of proteins acting on the MAP3K level (like on MEKK1, MLK1/2, or KSR2) would allow direct feedback control of MAPK pathways (Flotho *et al*, 2004). However, the majority of novel hits appear to lie outside the core MAPK pathway module, and these are probably simple downstream elements (i.e., substrates). Most of the novel proteins are expected to be either direct MAPK substrates or scaffold proteins (i.e., enabling phosphorylation of indirect MAPK substrates through protein complexes) (Fig EV5).

The wide distribution of D‐motifs in a functionally diverse set of proteins explains how MAPKs can regulate such a broad spectrum of physiological processes. Previously, their specific regulatory roles were often attributed to single target proteins. For example, the role of JNK in axonal growth was attributed to the JNK–JIP1 interaction, and the association of JNK with diabetes was attempted to be explained by the JNK1–IRS1 interaction alone (Lee *et al*, 2003; Dajas‐Bailador *et al*, 2008). In contrast, our results imply that these interactions may only be two examples of a substantially more complex protein network and JNK (as all MAPKs) connects to its targeted physiological systems by a large number of direct interactions. While individual connections might not be stable (especially in an evolutionary sense), multiple specific linkages could provide the key mechanism for a robust and adaptable physiological regulation.

Surprisingly, many of the newly implied MAPK partners have a restricted expression pattern enabling fine‐tuned regulation in specialized tissues. Because of the latter phenomenon, a great deal of these interactions is unlikely to be discovered by large‐scale protein–protein interaction screens. Easy‐to‐handle cell lines and mass‐spectrometry‐based analyses provide a powerful tool, but not for proteins that are only expressed in special, differentiated tissues (e.g., AAKG2, which is only abundant in cardiomyocytes) or restricted to certain embryonic developmental stages (e.g., DCX is almost exclusively expressed in developing neuroblasts) (Lang *et al*, 2000; Brown *et al*, 2003). Here, a modeling‐driven interactome search is the most suitable tool to fill in the gaps in our knowledge. In addition, a reliable sequence‐based prediction procedure sets the stage for easy examination on how MAPK signaling partners changed over time during evolution.

A comparative analysis suggests that rapid changes happened to MAPK pathways during the early evolution of vertebrates. Target proteins could have been brought under tight MAPK control by simply introducing docking motifs; however, this also necessitated target sites where phosphorylation could elicit functional effects. Similarly, existing links could have been thrown away by the loss of docking mechanisms. These processes were apparently the fastest when early vertebrates diverged from other chordates. Since then, the rate of motif emergence seemingly slowed down, but it has not completely stopped (some novel human motifs could be traced back only to mammals).

The current study explored MAPK–partner protein interactions in the human proteome. The identified D‐motifs have the capacity to bind specific MAPKs *in vitro* and likely also in cells and in full protein context. Their functional relevance, however, remains largely unexplored. Undoubtedly, further studies will be required to address the relationship between these physical recruitment sites and MAPK phosphorylation target sites to understand how MAPK‐mediated phosphorylation could elicit specific regulation. Nevertheless, this study suggests a rich and dramatically fast‐evolving landscape for short recruitment sites and helps to explain how MAPKs could have become such widespread regulators of cellular physiology.

## Materials and Methods

### Motif scan and filtering by ANCHOR

Putative MAPK‐binding D‐motif instances were collected from the human proteome. Protein sequences were downloaded from the reviewed section of the UniProt database. The resulting Human Proteome Database (HPD) contains 20,248 sequences. The HPD was scanned for motif hits with basic pattern matching using the regular expressions of seven different D‐motif classes/types (see Fig 1 and Appendix Fig S2), yielding 87,857 hits (JIP1‐class, NFAT4‐class, MEF2A‐class‐MEF2A‐type, MEF2A‐class‐MKK6‐type, DCC‐class‐generic, HePTP‐class‐Ste7‐type, HePTP‐class‐HePTP‐type). These hits were filtered using specific bioinformatics predictors aiming at selecting for biologically relevant instances. The filtering procedure followed a stepwise fashion as outlined below. Step 1: Filtering for the tendency to be part of disordered region that can undergo a disorder‐to‐order transition. The estimation of the interaction potential of the selected protein regions was done with the ANCHOR algorithm, a method trained to recognize binding regions in disordered protein segments (Mészáros *et al*, 2009). As described earlier, in linear motif selection, a more permissive version of ANCHOR can be used; therefore, the default 0.5 cutoff value was lowered in an adaptive way so that at least 90% of the known 47 formerly known D‐motifs are retained (Mészáros *et al*, 2012). Motif hits were kept only if they overlapped with either an ANCHOR region predicted by using a 0.4 cutoff, or an ANCHOR region predicted by using a 0.3 cutoff, but in this case at least one of the 20 residue flanking regions of the motif hit had to have a sufficiently high average disorder value (above 0.45) predicted with IUPred (Dosztányi *et al*, 2005). As a result, the number of hits was reduced to 21,201. Step 2: Filtering for intracellular accessibility. Motif hits were discarded if they resided in proteins that were predicted to have a signal peptide by SignalP, and if they were also predicted not to have a transmembrane region predicted by Phobius (Käll *et al*, 2007; Petersen *et al*, 2011). These motif hits were predicted to be localized outside of the cell, which is incompatible with MAPK binding. Phobius alone was also allowed to predict signal peptides alone, if SignalP score were not too low (above 0.3). If a motif instance resided in a protein that was predicted to have a signal peptide but it also had at least one transmembrane region, the localization of the motif region was further checked. If it was entirely intracellular, it was kept, otherwise discarded. This filtering step reduced the number of motif hits to 18,952. Step 3: Filtering for correct localization. All hits that were predicted by WoLF PSORT to be extracellular (with score ≥ 25), membrane protein (with score ≥ 25), localized to the E.R. (with score ≥ 15) or the Golgi (with score ≥ 9) were filtered out, unless they harbored transmembrane regions, and the region containing the motif was predicted to be localized in the intracellular space (Horton *et al*, 2007). There were 18,637 hits remaining after this step. Step 4: Filtering for structural accessibility. Motif hits that were determined to reside in Pfam domain regions were discarded (Finn *et al*, 2014). Some hits were also discarded in a manual curation process if they were located in Pfam Family/Repeat/Motif regions likely to have a stable structure in isolation. Furthermore, motif occurrences that overlapped with coiled‐coil regions predicted by COILS were removed as well (Lupas *et al*, 1991). Finally, there were 14,062 motifs remaining for further analysis including more than 90% of the known positives. Motifs passing all filters together with known positive hits are listed in Table EV1.

Compared to classical D‐motifs, a “mirror image” like orientation for certain MAPK‐binding motifs has also been described. In these “reverse docking” (revD) motifs, the hydrophobic stretch is located N‐terminally to the charged residues. Apart from a short revD motif in PEA‐15, all other mammalian examples are from a single group of MAPK interactors, the RSK/MAPKAPK family of kinases (Mace *et al*, 2013; Alexa *et al*, 2015). The low number of known reverse docking motifs, however, precluded their analysis in any systematic way.

### Scoring for structural compatibility by FoldX

Complexes of peptides with a MAPK were modeled using FoldX, similarly as it was previously described for SH2‐binding peptides (Schymkowitz *et al*, 2005; Sánchez *et al*, 2008). For some motif classes, a reliable crystallographic model was available because the JNK1‐pepJIP1 and JNK‐pepNFAT4 complex crystal structures contained full consensus motifs (Heo *et al*, 2004; Garai *et al*, 2012). For p38α‐pepMEF2A, p38α‐pepMKK6, and ERK2‐pepDCC crystal structures, the N‐terminal positively charged amino acids binding in the CD groove were not located in the electron density, so for these cases the modeling was done only for the hydrophobic portion of the motif (Chang *et al*, 2002; Ma *et al*, 2010; Garai *et al*, 2012). The ERK2‐pepHePTP complex contained artificial disulfide bond at the Φ_B_ position that facilitated crystallization of this protein–peptide complex; however, it also distorted the conformation of the docking peptide at the C‐terminal region (Zhou *et al*, 2006). This was corrected by remodeling this part of the peptide by FlexPepDock. The model for the hypothetical Ste7 model was constructed from the yeast Fus3‐pepSte7 and Fus3‐pepMsg5 structures, superimposed on human ERK2, and then optimized with FlexPepDock (Reményi *et al*, 2005; London *et al*, 2011). List of the PDB files used to generate the various MAPK‐docking motif structural templates are listed in Appendix Table S1. Each model contained the core motif with one extra alanine on both sides. The final FoldX score took into account the estimated energy of the complex as well as peptide stability in solution.

### Automated evolutionary sequence conservation analysis

All motifs were checked for evolutionary conservation. Sequence conservation, relative motif sequence conservation with respect to its flanking regions, and the maximum traceable evolutionary distance of the motif (maximum distance between species bearing the same motif in closely related proteins) were all calculated from databases with pre‐computed alignments. This was necessary to be able to compare the conservation of novel hits versus known motifs. For each protein with a potential motif, a cluster of orthologous proteins was extracted from the eggNOG database, using all vertebrates as the reference set of species (Jensen *et al*, 2008). Additionally, homologs from the inParanoid database were considered (Berglund *et al*, 2008). Here, the reference set of species consisted, in addition to human, of *Pan troglodytes*,* Mus musculus*,* Gallus gallus*,* Xenopus tropicalis*,* Danio rerio*,* Ciona intestinalis,* and *Brachiostoma floridae*. For each extracted cluster, only those sequences were retained that contained a motif instance within 10 or 50 amino acids compared to the human motif occurrence in full‐length alignments in the eggNOG and inParanoid clusters, respectively. The regions containing the motif with its 10 amino acid flanks on either sides were retained and realigned using MUSCLE (Edgar, 2004).

### PSSM building, sequence logos, and final scoring

Position‐specific scoring matrices for JIP1, NFAT4, greater MEF2A, and greater DCC classes were built including formerly known and newly found, validated human motif instances as well as all their identifiable vertebrate orthologs. To increase the sequence space coverage, we included more than just (known or novel) human MAPK‐docking motifs. A method was devised to use evolutionarily weighted sequences for each independently evolved (or sufficiently unique) motif and to collect all known vertebrate orthologs. For this purpose, alignments were built from vertebrate proteins obtained by BLAST searches. Based on the refined consensus, motifs were classified as either potentially functional or non‐functional. The motifs deemed potentially functional were realigned (with no gaps allowed) to the original sequence. In the end, the sequences were weighted by their evolutionary distance (based on the phylogenetic distances of the corresponding vertebrate organisms) and the final frequencies were obtained by summing up all independent groups with equal weights. In PSSM, each row represents one of 20 possible residues, and each column represents a position in a motif. Thus, the score for residue *X* at position *i* is defined in the following way:

$$X_{i}=\frac{\sum_{s}\left(w_{s}\times I\left(s_{i}=X\right)\right)+p\times X_{b}}{\sum_{s}w_{s}+p},$$

where *s* is a peptide sequence, *w*_*s*_ is the weight of that sequence based on the species from which it stems, *I* is the indicator function which is 1 when its argument is true and 0 otherwise, *p* is the pseudo‐count defined as square root of total number of training peptides from the class and it is used to account for residues that do not appear at position *i*, and *X*_*b*_ is the background frequency of the residue (based on UniProtKB/Swiss‐Prot Release 2013.05). For computational efficiency and to account for background frequencies of residues, log‐odds scores of *X*_*i*_ were used in the form *X*_*i*_ *= *log (*X*_*i*_ */X*_*b*_). The final score was calculated as the sum of the log‐odds scores of individual positions.

The distance between species A and B was the direct sum of branch lengths leading from A endpoint to B endpoint along the phylogenetic tree. These distances were the same that appear in PhyloWidget visualization of Ensembl data (Jordan & Piel, 2008). In case of species not included there, evolutionary distances were extracted from trees published in literature and numerically re‐calculated to fit the scaling of Ensembl‐derived distances wherever possible (Barley *et al*, 2010; Agnarsson *et al*, 2011; Betancur‐R *et al*, 2013). In the remaining few species where the tree topology was available without exact distance metrics, a numeric interpolation with equal weights was used.

Sequence logos were constructed using Seq2Logo‐2.0 (Thomsen & Nielsen, 2012). Height of residue *X* at position *i* is directly proportional to its PSSM score *X*_*i*_ (with *p = 0*) and *R*_*i*_, information content of position *i*, defined as:

$$R_{i}=\mathrm{lo}g_{2}\left(20\right)-\left(H_{i}+e_{n}\right),$$

where *e*_*n*_ is small sample correction parameter, expressed as 19/(2 * ln(2) * number of peptides), and *H*_*i*_ is uncertainty at position *i*, defined as:

$$H_{i}=-\sum X_{i}\times \mathrm{lo}g_{2}\left(X_{i}\right).$$

Receiver operating characteristic (ROC) curve was constructed by adding simulated negative cases: Peptides in the human proteome conforming to the respective D‐motif consensus but lying in a Pfam A‐structured region were scored together with validated true‐positive motifs, and at every value of the true‐positive rate, the corresponding value of the false‐positive rate was calculated and plotted (Finn *et al*, 2014). The area under the ROC (AUC) curve was calculated to assess the quality of the prediction: the closer it is to 1, the better is the predictor, and 0.9 indicates a very good predictor. For each motifs class, AUC calculation was performed in fivefold cross‐validation setting with 100 samples, selecting 4 folds of D‐motifs for training and 1 remaining fold, together with the D‐motifs from Pfam A domains, for testing, while not allowing motifs from the same vertebrate orthology set to be used for both training and testing.

### Analysis on D‐motif and phosphorylation site conservation

Consensus MAPK phosphorylation sites from proteins, which contain an experimentally verified D‐motif (Table EV3), were collected from PhosphoSitePlus database (Li *et al*, 2002). Phosphorylation sites was selected if it was more than 10 amino acids away from the core D‐motif and if there was site specific or mass‐spectrometry experimental evidence of phosphorylation at that site in human. Normalized average traceable evolutionary distance for these sites from eggNOG vertebrate alignments has been estimated as the sum of the weights of the organisms where the phosphorylation site was present in the homologous protein divided by the sum of all the weights from the organisms in the alignment (Table EV4). If multiple candidate MAPK phosphorylation sites were present in the protein, the site with maximum normalized average traceable evolutionary distance was selected.

### Protein expression and purification

Activated ERK2, p38α, and JNK1 were produced by co‐expressing the MAPKs with constitutively active forms of GST‐tagged MAPK kinases (MKK1, MKK6, and MKK7, respectively) in *E. coli* (Rosetta pLysS). Full‐length human MAPKs and their corresponding activator kinases were encoded in bicistronic vectors allowing the expression of hexa‐histidine‐tagged, phosphorylated MAPKs. Proteins were purified similar to dephosphorylated MAPKs as described earlier, using a Ni‐column affinity and an ion‐exchange step (Garai *et al*, 2012). The double‐phosphorylated state of activated MAPK samples was confirmed by using anti‐phospho MAPK antibodies with Western blots. Fragments corresponding to the D‐motifs of various proteins (with appropriate flanks) were reverse‐translated to synthetic DNA fragments endowed with sticky BamHI‐ and NotI‐compatible ends. These were annealed, treated with PNK (polynucleotide kinase), and ligated into a customized pET‐17 vector (pAZAD) containing flexible linkers (known to be intrinsically disordered) derived from ATF2 (Nagadoi *et al*, 1999). The linkers were incorporated directly between the GST‐tag and the D‐motif cassette as well as between the docking cassette and the target site. GST fusion constructs contained an ATF2‐derived phosphorylatable epitope, lacking the T69 site, with only the T71 site present (ADQAPTPTRFL). All constructs were checked by DNA sequencing. The resulting GST‐tagged artificial substrates with a C‐terminal hexa‐histidine‐tag were expressed in *E. coli,* purified first on Ni‐Sepharose and on glutathione‐Sepharose by affinity chromatography. To prevent degradation of unstructured protein segments, lysates were treated with protease inhibitor cocktails (cOmplete EDTA‐free inhibitor cocktail tablet, Roche), phenylmethanesuphonyl fluoride (PMSF) and benzamidine. All buffers following Ni‐affinity purification contained PMSF (0.4 mM), benzamidine (2 mM), and EDTA (1 mM). Since detergents might influence immobilization efficiency, care was taken to use the same detergent, BOG (Octyl‐β‐D‐glucopyranozide), in all final solutions (including dilutions) at the same concentration (0.02%).

### *In vitro* dot‐blot phosphorylation assay

Stocks containing the purified artificial substrates were diluted to equal concentrations (~1 mg/ml), and printed (1 μl) as triplicates on nitrocellulose membrane using a pipetting robot (Hamilton STARlet). Membranes were dried at room temperature for at least 1 h. Prior to phosphorylation, membranes were blocked in Tris‐buffered saline and Tween‐20 buffer (TBS‐T) containing 3% bovine serum albumin (BSA) for 30 min and washed three times with TBS‐T. Phosphorylation was performed in kinase buffer containing 50 mM TRIS–HCl (pH = 7.5), 10 mM MgCl_2_, 2 mM DTT, 0.1% BSA, and 2 mM ATP. Activated MAPKs were applied in 100–300 nM concentration. The phosphorylation solution was pre‐mixed before applying onto the membrane. The reaction took place at room temperature on a rocker, it was stopped after 10 min by the addition of EDTA (at 25 mM end concentration), and membranes were then washed three times with TBS‐T. Thereafter, membranes were blocked again by 3% BSA in TBS‐T and developed by standard Western blot techniques using an anti‐phospho‐T71 ATF2 antibody (Cell Signaling Technology, #9221S) at 1:1,000 dilution and a secondary anti‐rabbit antibody (Cell Signaling Technology, #7074S) at 1:2,000 dilution. After development with the Immobilon ECL kit (Millipore), phosphorylation signal was read either by luminescence (Alpha Innotech gel documentation system) or by fluorescence (Typhoon Trio+ scanner, GE). Non‐phosphorylated membranes were also checked for protein immobilization efficiency. The C‐terminal hexa‐histidine epitopes of GST phosphorylation reporter constructs were detected by a standard anti‐His6 Western blot. Dot‐blot experiments were performed for each construct at least twice, using different protein stocks. Only those constructs that consistently performed in all experiments above the non‐cognate control were regarded as positive.

### *In vitro* protein–peptide binding assays

For fluorescence polarization (FP)‐based binding affinity measurements, known MAPK‐docking groove‐binding peptides were N‐terminally labeled with carboxyfluorescein or carboxytetramethylrhodamine (for ERK2, pepRSK1: PQLKPIESSILAQRRVRKLPSTTL; for p38α, pepMK2: IKIKKIEDASNPLLLKRRKK or pepMEF2A: SRKPDLRVVIPPS; for JNK1, pep JIP3: RKERPTSLNFPL). 50 nM labeled reporter peptide was mixed with MAPKs in a concentration to achieve ~60–80% complex formation. Subsequently, increasing amounts of chemically synthesized test peptides were added, and the FP signal was measured with a Synergy H4 (BioTek Instruments) plate reader in 384‐well plates. The Ki for each unlabeled peptide was determined by fitting the data to a competition binding equation. Titration experiments were carried out in triplicates, and the average FP signal was used for fitting the data with OriginPro7.

### Cell‐based protein–protein interaction assay

The full‐length cDNA of yellow fluorescent protein (YFP) was split at position 159, and fragments (F1 and F2) were pasted into pcDNA 3.1 vectors (Invitrogen). ERK2 was expressed as C‐terminal and p38α and JNK1 as N‐terminal F2 fusions. To facilitate expression, JNK1 and p38α had kinase‐inactivating mutations (K55R and K53R, respectively), while ERK2 was wild type. The ERK2 and JNK1 constructs contained a FLAG‐tag, but similarly tagged p38α constructs could not be expressed to a comparable extent. Therefore, expression levels of F2‐p38α could only be monitored by an anti‐p38α antibody. MAPK partners were expressed as N‐terminal and C‐terminal F1 fusions with FLAG‐tags. F1 and F2 fusion pairs that gave the highest BiFc signal with wild‐type MAPK partners were chosen to analyze the impact of docking motif truncations or mutations. These were introduced into full‐length MAPK partner constructs by PCR or by the QuickChange method. N‐terminal truncations were made in proteins with N‐terminal docking motifs: MKK1(14‐393), MKK6(18‐334), RHDF1(26‐855), MKP5(28‐482), and FAM122A (14‐287) and a 20‐amino‐acid‐long C‐terminal truncation was used to generate the DCX(1‐343) construct. Mutation of multiple residues was utilized for proteins with internal motifs: JIP1(R160A,P161A,L164A,L166A) and APBA2(R280A,P281A,L284A,L286A), and K331, K332, K333, and L337 were mutated to alanines in the short KSR2(325‐399) construct All sequences were verified by DNA sequencing. HEK293T cells were cultured in Dulbecco's modified Eagle's medium (DMEM, Lonza) containing 10% fetal bovine serum and 1% penicillin/streptomycin at 37°C in an atmosphere of 5% CO_2_ in 25‐cm^2^ tissue culture flasks (Orange Scientific). Cells were seeded onto 96‐well plate (tissue culture test plate 96F, TPP) at 60–70% confluence 24 h prior to transfection. The medium was changed to serum‐free OPTI‐MEM (Gibco). Transient transfections with Lipofectamine 2000 reagent (Invitrogen) were carried out according to the manufacturer's instruction. Cells were assayed 2 days post‐transfection. For BiFc signal intensity measurements, cells were washed and suspended in 100 μl PBS. Twenty microliters of this cell suspension (~20,000 cells) was aliquoted into a 384‐well black‐sided plate. Fluorescence intensity per well was measured using a Synergy H4 (BioTek Instruments) fluorescence plate reader (excitation/emission wavelength was 515/535 nm). Cells from 50 μl of the PBS suspension were collected, and samples were subjected to Western blots using anti‐FLAG‐tag antibody (Sigma, F1804). For imaging, transfected cells were examined with an Olympus IX81 microscope using an Olympus FluoView 500 confocal laser scanning microscope system (Hamburg, Germany). YFP fluorescence was imaged using 514‐nm excitation and a 535‐ to 560‐nm emission filter.

### Cell‐based assays for EGF stimulation and monitoring GAB1 localization

GAB1 constructs were subcloned into modified pCerulean‐C1 vector with N‐terminal Cerulean fluorescent protein and C‐terminal FLAG fusion tags. HEK293T cells were cultured and transfected similarly as described above. Cells were transfected with 0.2 μg GAB1 DNA constructs and were serum‐starved for 24 h. The media were removed after 40 h from DNA transfection and 100 μl PBS was added to wells. ERK pathway stimulation was started by the addition of epidermal growth factor (EGF, Sigma, E9644) at 20 ng/ml concentration to each well, and stimulation was terminated at different time points by adding 35 μl of 4× SDS loading buffer to wells. Cells were lysed and 10 μl of each sample was subjected to SDS–PAGE and transferred to PVDF membrane (Sigma, P2563). Western blotting for monitoring total ERK1/2 and GAB1 was done by using anti‐p42/44 MAPK (Cell Signaling, #4695) and anti‐FLAG antibody (Sigma, F1804), respectively. After stripping the membrane, phospho‐p44/42 MAPK (ERK1/2) (Thr202/Tyr204) antibody (Cell Signaling, #9101) was used to detect ppERK1/2 protein levels. For secondary antibody, anti‐rabbit HRP‐linked antibody (Cell Signaling, #7074) and anti‐mouse HRP‐linked antibody (Calbiochem, #401215) were used. The ECL signal was quantified with an Alpha Innotech Fluorchem FC2 instrument.

For visualizing GAB1 localization analysis, transfected cells were examined by Zeiss LSM 710 confocal laser scanning microscope using 405‐nm laser for excitation in chambered borosilicate cover glass (Lab‐Tek, #155411). Evaluation of CFP‐GAB1 localization was done using ImageJ. Cells were treated by 100 ng/ml epidermal growth factor (EGF) for 10 min.

### *In vitro* kinase assays

Activated JNK and p38α were produced by co‐expressing the MAPKs with constitutively active forms of GST‐tagged MAPK kinases (MKK7 and MKK6, respectively) in *E. coli* with bicistronic modified pET vectors. DCX and AAKG2 proteins and their mutants were produced in *E. coli* using modified pET vectors with C‐terminal His‐tag and N‐terminal MBP‐tag or GST‐tag, respectively. Proteins were purified with double‐affinity chromatography using Ni and MPB or GST column steps.

Twenty nM activated MAPK was incubated with 400 nM DCX or AAKG2 proteins at room temperature in the presence of 1 mg/ml BSA. Kinase reactions were carried out in 50 mM HEPES pH 7.5, 100 mM NaCl, 5 mM MgCl_2_, 0.05% IGEPAL, 5% glycerol, 2 mM DTT in the presence of 250 μM ATP, and ~5 μCi of ATP(γ32P). Reactions were stopped with protein loading sample buffer complemented with 100 mM EDTA, boiled, and then subjected to SDS–PAGE. The dried gel was analyzed by phosphorimaging on a Typhoon Trio+ scanner (GE Healthcare). Competitor docking motif peptides were chemically synthesized and used in 10 μM concentration (pepMK2, specific to p38: IKIKKIEADASNPLLLKRRKK; and pepJIP1, specific to JNK: DTYRPKRPTTLNLFP).

## Author contributions

AZ and AR designed the study. AZ, AA, ÁG, and KK performed experimental work. TB, BM, and OVK performed *in silico* analysis. AZ, OVK, ZD, and AR analyzed data. AZ and AR wrote the paper. All authors read and approved the manuscript.

## Conflict of interest

The authors declare that they have no conflict of interest.

## Supporting information



Appendix

Expanded View Figures PDF

Table EV1

Table EV2

Table EV3

Table EV4

Review Process File

Source Data for Figure 2

## References

[msb156269-bib-0001] Agnarsson I , Zambrana‐Torrelio CM , Flores‐Saldana NP , May‐Collado LJ (2011) A time‐calibrated species‐level phylogeny of bats (Chiroptera, Mammalia). PLoS Curr 3: RRN1212 21327164 10.1371/currents.RRN1212 PMC3038382

[msb156269-bib-0002] Alexa A , Gógl G , Glatz G , Garai Á , Zeke A , Varga J , Dudás E , Jeszenői N , Bodor A , Hetényi C , Reményi A (2015) Structural assembly of the signaling competent ERK2–RSK1 heterodimeric protein kinase complex. Proc Natl Acad Sci 112: 201417571 10.1073/pnas.1417571112 PMC4352816 25730857

[msb156269-bib-0003] Alexandropoulos K , Baltimore D (1996) Coordinate activation of c‐Src by SH3‐ and SH2‐binding sites on a novel p130Cas‐related protein. Sin Genes Dev 10: 1341–1355 8647432 10.1101/gad.10.11.1341

[msb156269-bib-0004] Bandyopadhyay S , Chiang C , Srivastava J , Gersten M , White S , Bell R , Kurschner C , Martin CH , Smoot M , Sahasrabudhe S , Barber DL , Chanda SK , Ideker T (2010) A human MAP kinase interactome. Nat Methods 7: 801–805 20936779 10.1038/nmeth.1506 PMC2967489

[msb156269-bib-0005] Bardwell L (2006) Mechanisms of MAPK signalling specificity. Biochem Soc Trans 34: 837–841 17052210 10.1042/BST0340837 PMC3017501

[msb156269-bib-0006] Barley AJ , Spinks PQ , Thomson RC , Shaffer HB (2010) Fourteen nuclear genes provide phylogenetic resolution for difficult nodes in the turtle tree of life. Mol Phylogenet Evol 55: 1189–1194 19913628 10.1016/j.ympev.2009.11.005

[msb156269-bib-0008] Berglund A‐C , Sjölund E , Ostlund G , Sonnhammer ELL (2008) InParanoid 6: eukaryotic ortholog clusters with inparalogs. Nucleic Acids Res 36: D263–D266 18055500 10.1093/nar/gkm1020 PMC2238924

[msb156269-bib-0009] Betancur‐R R , Broughton RE , Wiley EO , Carpenter K , López JA , Li C , Holcroft NI , Arcila D , Sanciangco M , Cureton IJ , Zhang F , Buser T , Campbell MA , Ballesteros JA , Roa‐Varon A , Willis S , Borden WC , Rowley T , Reneau PC , Hough DJ *et al* (2013) The tree of life and a new classification of bony fishes. PLoS Curr 5: 1–33 10.1371/currents.tol.53ba26640df0ccaee75bb165c8c26288 PMC3644299 23653398

[msb156269-bib-0010] Biondi RM , Nebreda AR (2003) Signalling specificity of Ser/Thr protein kinases through docking‐site‐mediated interactions. Biochem J 372: 1–13 12600273 10.1042/BJ20021641 PMC1223382

[msb156269-bib-0012] Brown JP , Couillard‐Després S , Cooper‐Kuhn CM , Winkler J , Aigner L , Kuhn HG (2003) Transient expression of doublecortin during adult neurogenesis. J Comp Neurol 467: 1–10 14574675 10.1002/cne.10874

[msb156269-bib-0014] Carlson SM , Chouinard CR , Labadorf A , Lam CJ , Schmelzle K , Fraenkel E , White FM (2011) Large‐scale discovery of ERK2 substrates identifies ERK‐mediated transcriptional regulation by ETV3. Sci Signal 4: rs11 22028470 10.1126/scisignal.2002010 PMC3779841

[msb156269-bib-0015] Chang CI , Xu B , Akella R , Cobb MH , Goldsmith EJ (2002) Crystal structures of MAP kinase p38 complexed to the docking sites on its nuclear substrate MEF2A and activator MKK3b. Mol Cell 9: 1241–1249 12086621 10.1016/s1097-2765(02)00525-7

[msb156269-bib-0016] Chen W‐K , Yeap YYC , Bogoyevitch MA (2014) The JNK1/JNK3 interactome ‐ Contributions by the JNK3 unique N‐terminus and JNK common docking site residues. Biochem Biophys Res Commun 453: 576–581 25301550 10.1016/j.bbrc.2014.09.122

[msb156269-bib-0017] Chow CW , Rincón M , Cavanagh J , Dickens M , Davis RJ (1997) Nuclear accumulation of NFAT4 opposed by the JNK signal transduction pathway. Science 278: 1638–1641 9374467 10.1126/science.278.5343.1638

[msb156269-bib-0019] Costanzo‐Garvey DL , Pfluger PT , Dougherty MK , Stock JL , Boehm M , Chaika O , Fernandez MR , Fisher K , Kortum RL , Hong E‐G , Jun JY , Ko HJ , Schreiner A , Volle DJ , Treece T , Swift AL , Winer M , Chen D , Wu M , Leon LR *et al* (2009) KSR2 is an essential regulator of AMP kinase, energy expenditure, and insulin sensitivity. Cell Metab 10: 366–378 19883615 10.1016/j.cmet.2009.09.010 PMC2773684

[msb156269-bib-0020] Courcelles M , Frémin C , Voisin L , Lemieux S , Meloche S , Thibault P (2013) Phosphoproteome dynamics reveal novel ERK1/2 MAP kinase substrates with broad spectrum of functions. Mol Syst Biol 9: 669 23712012 10.1038/msb.2013.25 PMC4188273

[msb156269-bib-0021] Dajani R , Fraser E , Roe SM , Yeo M , Good VM , Thompson V , Dale TC , Pearl LH (2003) Structural basis for recruitment of glycogen synthase kinase 3beta to the axin‐APC scaffold complex. EMBO J 22: 494–501 12554650 10.1093/emboj/cdg068 PMC140752

[msb156269-bib-0022] Dajas‐Bailador F , Jones EV , Whitmarsh AJ (2008) The JIP1 scaffold protein regulates axonal development in cortical neurons. Curr Biol 18: 221–226 18261906 10.1016/j.cub.2008.01.025 PMC2258314

[msb156269-bib-0023] De Nicola GF , Martin ED , Chaikuad A , Bassi R , Clark J , Martino L , Verma S , Sicard P , Tata R , Atkinson RA , Knapp S , Conte MR , Marber MS (2013) Mechanism and consequence of the autoactivation of p38α mitogen‐activated protein kinase promoted by TAB 1. Nat Struct Mol Biol 20: 1182–1190 24037507 10.1038/nsmb.2668 PMC3822283

[msb156269-bib-0025] Dinkel H , Van Roey K , Michael S , Davey NE , Weatheritt RJ , Born D , Speck T , Krüger D , Grebnev G , Kuban M , Strumillo M , Uyar B , Budd A , Altenberg B , Seiler M , Chemes LB , Glavina J , Sánchez IE , Diella F , Gibson TJ (2014) The eukaryotic linear motif resource ELM: 10 years and counting. Nucleic Acids Res 42: D259–D266 24214962 10.1093/nar/gkt1047 PMC3964949

[msb156269-bib-0026] Dosztányi Z , Csizmók V , Tompa P , Simon I (2005) The pairwise energy content estimated from amino acid composition discriminates between folded and intrinsically unstructured proteins. J Mol Biol 347: 827–839 15769473 10.1016/j.jmb.2005.01.071

[msb156269-bib-0027] Dosztányi Z , Mészáros B , Simon I (2009) ANCHOR: web server for predicting protein binding regions in disordered proteins. Bioinformatics 25: 2745–2746 19717576 10.1093/bioinformatics/btp518 PMC2759549

[msb156269-bib-0028] Durand D (2003) Vertebrate evolution: doubling and shuffling with a full deck. Trends Genet 19: 2–5 12493240 10.1016/s0168-9525(02)00008-2

[msb156269-bib-0029] Edgar RC (2004) MUSCLE: multiple sequence alignment with high accuracy and high throughput. Nucleic Acids Res 32: 1792–1797 15034147 10.1093/nar/gkh340 PMC390337

[msb156269-bib-0030] Estrella NL , Desjardins CA , Nocco SE , Clark AL , Maksimenko Y , Naya FJ (2015) MEF2 Transcription Factors Regulate Distinct Gene Programs in Mammalian Skeletal Muscle Differentiation. J Biol Chem 290: 1256–1268 25416778 10.1074/jbc.M114.589838 PMC4294490

[msb156269-bib-0031] Eulenfeld R , Schaper F (2009) A new mechanism for the regulation of Gab1 recruitment to the plasma membrane. J Cell Sci 122: 55–64 19050043 10.1242/jcs.037226

[msb156269-bib-0032] Fantz DA , Jacobs D , Glossip D , Kornfeld K (2001) Docking sites on substrate proteins direct extracellular signal‐regulated kinase to phosphorylate specific residues. J Biol Chem 276: 27256–27265 11371562 10.1074/jbc.M102512200

[msb156269-bib-0033] Fernandes N , Bailey DE , VanVranken DL , Allbritton NL (2007) Use of Docking Peptides to Design Modular Substrates with High Efficiency for Mitogen‐Activated Protein Kinase Extracellular Signal‐Regulated Kinase. ACS Chem Biol 2: 665–673 17918909 10.1021/cb700158q PMC2597387

[msb156269-bib-0034] Finck BN , Kelly DP (2006) PGC‐1 coactivators: inducible regulators of energy metabolism in health and disease. J Clin Invest 116: 615–622 16511594 10.1172/JCI27794 PMC1386111

[msb156269-bib-0035] Finn RD , Bateman A , Clements J , Coggill P , Eberhardt RY , Eddy SR , Heger A , Hetherington K , Holm L , Mistry J , Sonnhammer ELL , Tate J , Punta M (2014) Pfam: the protein families database. Nucleic Acids Res 42: D222–D230 24288371 10.1093/nar/gkt1223 PMC3965110

[msb156269-bib-0036] Flotho A , Simpson DM , Qi M , Elion EA (2004) Localized feedback phosphorylation of Ste5p scaffold by associated MAPK cascade. J Biol Chem 279: 47391–47401 15322134 10.1074/jbc.M405681200

[msb156269-bib-0037] Garai Á , Zeke A , Gógl G , Törő I , Fördős F , Blankenburg H , Bárkai T , Varga J , Alexa A , Emig D , Albrecht M , Reményi A (2012) Specificity of linear motifs that bind to a common mitogen‐activated protein kinase docking groove. Sci Signal 5: ra74 23047924 10.1126/scisignal.2003004 PMC3500698

[msb156269-bib-0039] Glatz G , Gogl G , Alexa A , Remenyi A (2013) Structural Mechanism for the Specific Assembly and Activation of the Extracellular Signal Regulated Kinase 5 (ERK5) Module. J Biol Chem 288: 8596–8609 23382384 10.1074/jbc.M113.452235 PMC3605678

[msb156269-bib-0040] Goldman A , Roy J , Bodenmiller B , Wanka S , Landry CR , Aebersold R , Cyert MS (2014) The calcineurin signaling network evolves via conserved kinase‐phosphatase modules that transcend substrate identity. Mol Cell 55: 422–435 24930733 10.1016/j.molcel.2014.05.012 PMC4127121

[msb156269-bib-0041] Gordon EA , Whisenant TC , Zeller M , Kaake RM , Gordon WM , Krotee P , Patel V , Huang L , Baldi P , Bardwell L (2013) Combining docking site and phosphosite predictions to find new substrates: identification of smoothelin‐like‐2 (SMTNL2) as a c‐Jun N‐terminal kinase (JNK) substrate. Cell Signal 25: 2518–2529 23981301 10.1016/j.cellsig.2013.08.004 PMC4132694

[msb156269-bib-0042] Grewal S , Molina DM , Bardwell L (2006) Mitogen‐activated protein kinase (MAPK)‐docking sites in MAPK kinases function as tethers that are crucial for MAPK regulation in vivo. Cell Signal 18: 123–134 15979847 10.1016/j.cellsig.2005.04.001 PMC3017502

[msb156269-bib-0043] Heo Y‐S , Kim S‐K , Il SC , Kim YK , Sung B‐J , Lee HS , Il LJ , Park S‐Y , Kim JH , Hwang KY , Hyun Y‐L , Jeon YH , Ro S , Cho JM , Lee TG , Yang C‐H (2004) Structural basis for the selective inhibition of JNK1 by the scaffolding protein JIP1 and SP600125. EMBO J 23: 2185–2195 15141161 10.1038/sj.emboj.7600212 PMC419904

[msb156269-bib-0045] Hirosumi J , Tuncman G , Chang L , Görgün CZ , Uysal KT , Maeda K , Karin M , Hotamisligil GS (2002) A central role for JNK in obesity and insulin resistance. Nature 420: 333–336 12447443 10.1038/nature01137

[msb156269-bib-0046] Ho DT , Bardwell AJ , Grewal S , Iverson C , Bardwell L (2006) Interacting JNK‐docking sites in MKK7 promote binding and activation of JNK mitogen‐activated protein kinases. J Biol Chem 281: 13169–13179 16533805 10.1074/jbc.M601010200 PMC3017509

[msb156269-bib-0047] Holgado‐Madruga M , Emlet DR , Moscatello DK , Godwin AK , Wong AJ (1996) A Grb2‐associated docking protein in EGF‐ and insulin‐receptor signalling. Nature 379: 560–564 8596638 10.1038/379560a0

[msb156269-bib-0048] Horton P , Park K‐J , Obayashi T , Fujita N , Harada H , Adams‐Collier CJ , Nakai K (2007) WoLF PSORT: protein localization predictor. Nucleic Acids Res 35: W585–W587 17517783 10.1093/nar/gkm259 PMC1933216

[msb156269-bib-0049] Hulo N , Sigrist CJA , Le Saux V , Langendijk‐Genevaux PS , Bordoli L , Gattiker A , De Castro E , Bucher P , Bairoch A (2004) Recent improvements to the PROSITE database. Nucleic Acids Res 32: D134–D137 14681377 10.1093/nar/gkh044 PMC308778

[msb156269-bib-0050] Jacobs D , Glossip D , Xing H , Muslin AJ , Kornfeld K (1999) Multiple docking sites on substrate proteins form a modular system that mediates recognition by ERK MAP kinase. Genes Dev 13: 163–175 9925641 PMC316390

[msb156269-bib-0051] Jensen LJ , Julien P , Kuhn M , von Mering C , Muller J , Doerks T , Bork P (2008) eggNOG: automated construction and annotation of orthologous groups of genes. Nucleic Acids Res 36: D250–D254 17942413 10.1093/nar/gkm796 PMC2238944

[msb156269-bib-0052] Johnson GL , Lapadat R (2002) Mitogen‐activated protein kinase pathways mediated by ERK, JNK, and p38 protein kinases. Science 298: 1911–1912 12471242 10.1126/science.1072682

[msb156269-bib-0053] Jordan GE , Piel WH (2008) PhyloWidget: web‐based visualizations for the tree of life. Bioinformatics 24: 1641–1642 18487241 10.1093/bioinformatics/btn235

[msb156269-bib-0054] Jung US , Sobering AK , Romeo MJ , Levin DE (2002) Regulation of the yeast Rlm1 transcription factor by the Mpk1 cell wall integrity MAP kinase. Mol Microbiol 46: 781–789 12410835 10.1046/j.1365-2958.2002.03198.x

[msb156269-bib-0055] Käll L , Krogh A , Sonnhammer ELL (2007) Advantages of combined transmembrane topology and signal peptide prediction–the Phobius web server. Nucleic Acids Res 35: W429–W432 17483518 10.1093/nar/gkm256 PMC1933244

[msb156269-bib-0058] Kosako H , Yamaguchi N , Aranami C , Ushiyama M , Kose S , Imamoto N , Taniguchi H , Nishida E , Hattori S (2009) Phosphoproteomics reveals new ERK MAP kinase targets and links ERK to nucleoporin‐mediated nuclear transport. Nat Struct Mol Biol 16: 1026–1035 19767751 10.1038/nsmb.1656

[msb156269-bib-0060] Kragelj J , Palencia A , Nanao MH , Maurin D , Bouvignies G , Blackledge M , Jensen MR (2015) Structure and dynamics of the MKK7‐JNK signaling complex. Proc Natl Acad Sci USA 112: 3409–3414 25737554 10.1073/pnas.1419528112 PMC4371970

[msb156269-bib-0061] Kuraku S , Meyer A , Kuratani S (2009) Timing of genome duplications relative to the origin of the vertebrates: did cyclostomes diverge before or after? Mol Biol Evol 26: 47–59 18842688 10.1093/molbev/msn222

[msb156269-bib-0063] Lang T , Yu L , Tu Q , Jiang J , Chen Z , Xin Y , Liu G , Zhao S (2000) Molecular Cloning, Genomic Organization, and Mapping of PRKAG2, a Heart Abundant γ2 Subunit of 5′‐AMP‐Activated Protein Kinase, to Human Chromosome 7q36. Genomics 70: 258–263 11112354 10.1006/geno.2000.6376

[msb156269-bib-0064] Laughlin JD , Nwachukwu JC , Figuera‐Losada M , Cherry L , Nettles KW , LoGrasso PV (2012) Structural mechanisms of allostery and autoinhibition in JNK family kinases. Structure 20: 2174–2184 23142346 10.1016/j.str.2012.09.021 PMC3589125

[msb156269-bib-0065] Lee YH , Giraud J , Davis RJ , White MF (2003) c‐Jun N‐terminal kinase (JNK) mediates feedback inhibition of the insulin signaling cascade. J Biol Chem 278: 2896–2902 12417588 10.1074/jbc.M208359200

[msb156269-bib-0066] Lee KS , Park J‐E , Kang YH , Kim T‐S , Bang JK (2014) Mechanisms underlying Plk1 polo‐box domain‐mediated biological processes and their physiological significance. Mol Cells 37: 286–294 24722413 10.14348/molcells.2014.0002 PMC4012076

[msb156269-bib-0068] Li J , Ning Y , Hedley W , Saunders B , Chen Y , Tindill N , Hannay T , Subramaniam S (2002) The Molecule Pages database. Nature 420: 716–717 12478304 10.1038/nature01307

[msb156269-bib-0069] Li L , Wang Y , Carr R , Haddad CS , Li Z , Qian L , Oberholzer J , Maker AV , Wang Q , Prabhakar BS (2014) IG20/MADD plays a critical role in glucose‐induced insulin secretion. Diabetes 63: 1612–1623 24379354 10.2337/db13-0707 PMC3994957

[msb156269-bib-0070] Lim WA , Richards FM , Fox RO (1994) Structural determinants of peptide‐binding orientation and of sequence specificity in SH3 domains. Nature 372: 375–379 7802869 10.1038/372375a0

[msb156269-bib-0071] Livingstone C , Patel G , Jones N (1995) ATF‐2 contains a phosphorylation‐dependent transcriptional activation domain. EMBO J 14: 1785–1797 7737129 10.1002/j.1460-2075.1995.tb07167.x PMC398272

[msb156269-bib-0072] London N , Raveh B , Cohen E , Fathi G , Schueler‐Furman O (2011) Rosetta FlexPepDock web server–high resolution modeling of peptide‐protein interactions. Nucleic Acids Res 39: W249–W253 21622962 10.1093/nar/gkr431 PMC3125795

[msb156269-bib-0073] Lowe ED , Tews I , Cheng KY , Brown NR , Gul S , Noble MEM , Gamblin SJ , Johnson LN (2002) Specificity determinants of recruitment peptides bound to phospho‐CDK2/cyclin A. Biochemistry 41: 15625–15634 12501191 10.1021/bi0268910

[msb156269-bib-0075] Lupas A , Van Dyke M , Stock J (1991) Predicting coiled coils from protein sequences. Science 252: 1162–1164 2031185 10.1126/science.252.5009.1162

[msb156269-bib-0076] Ma W , Shang Y , Wei Z , Wen W , Wang W , Zhang M (2010) Phosphorylation of DCC by ERK2 is facilitated by direct docking of the receptor P1 domain to the kinase. Structure 18: 1502–1511 21070949 10.1016/j.str.2010.08.011

[msb156269-bib-0077] Mace PD , Wallez Y , Egger MF , Dobaczewska MK , Robinson H , Pasquale EB , Riedl SJ (2013) Structure of ERK2 bound to PEA‐15 reveals a mechanism for rapid release of activated MAPK. Nat Commun 4: 1681 23575685 10.1038/ncomms2687 PMC3640864

[msb156269-bib-0078] Mészáros B , Simon I , Dosztányi Z (2009) Prediction of protein binding regions in disordered proteins. PLoS Comput Biol 5: e1000376 19412530 10.1371/journal.pcbi.1000376 PMC2671142

[msb156269-bib-0079] Mészáros B , Dosztányi Z , Simon I (2012) Disordered binding regions and linear motifs–bridging the gap between two models of molecular recognition. PLoS One 7: e46829 23056474 10.1371/journal.pone.0046829 PMC3463566

[msb156269-bib-0082] Nagadoi A , Nakazawa K , Uda H , Okuno K , Maekawa T , Ishii S , Nishimura Y (1999) Solution structure of the transactivation domain of ATF‐2 comprising a zinc finger‐like subdomain and a flexible subdomain. J Mol Biol 287: 593–607 10092462 10.1006/jmbi.1999.2620

[msb156269-bib-0083] Ngo JCK , Chakrabarti S , Ding J‐H , Velazquez‐Dones A , Nolen B , Aubol BE , Adams JA , Fu X‐D , Ghosh G (2005) Interplay between SRPK and Clk/Sty kinases in phosphorylation of the splicing factor ASF/SF2 is regulated by a docking motif in ASF/SF2. Mol Cell 20: 77–89 16209947 10.1016/j.molcel.2005.08.025

[msb156269-bib-0084] Ohno S (1993) Patterns in genome evolution. Curr Opin Genet Dev 3: 911–914 8118217 10.1016/0959-437x(93)90013-f

[msb156269-bib-0086] Olson BL , Hock MB , Ekholm‐Reed S , Wohlschlegel JA , Dev KK , Kralli A , Reed SI (2008) SCFCdc4 acts antagonistically to the PGC‐1alpha transcriptional coactivator by targeting it for ubiquitin‐mediated proteolysis. Genes Dev 22: 252–264 18198341 10.1101/gad.1624208 PMC2192758

[msb156269-bib-0087] Pearce LR , Atanassova N , Banton MC , Bottomley B , van der Klaauw AA , Revelli J‐P , Hendricks A , Keogh JM , Henning E , Doree D , Jeter‐Jones S , Garg S , Bochukova EG , Bounds R , Ashford S , Gayton E , Hindmarsh PC , Shield JPH , Crowne E , Barford D *et al* (2013) KSR2 mutations are associated with obesity, insulin resistance, and impaired cellular fuel oxidation. Cell 155: 765–777 24209692 10.1016/j.cell.2013.09.058 PMC3898740

[msb156269-bib-0088] Pellicena P , Stowell KR , Miller WT (1998) Enhanced phosphorylation of Src family kinase substrates containing SH2 domain binding sites. J Biol Chem 273: 15325–15328 9624111 10.1074/jbc.273.25.15325

[msb156269-bib-0089] Petersen TN , Brunak S , von Heijne G , Nielsen H (2011) SignalP 4.0: discriminating signal peptides from transmembrane regions. Nat Methods 8: 785–786 21959131 10.1038/nmeth.1701

[msb156269-bib-0090] Podkowa M , Zhao X , Chow C‐W , Coffey ET , Davis RJ , Attisano L (2010) Microtubule stabilization by bone morphogenetic protein receptor‐mediated scaffolding of c‐Jun N‐terminal kinase promotes dendrite formation. Mol Cell Biol 30: 2241–2250 20176805 10.1128/MCB.01166-09 PMC2863594

[msb156269-bib-0091] Reményi A , Good MC , Bhattacharyya RP , Lim WA (2005) The role of docking interactions in mediating signaling input, output, and discrimination in the yeast MAPK network. Mol Cell 20: 951–962 16364919 10.1016/j.molcel.2005.10.030

[msb156269-bib-0092] Reményi A , Good MC , Lim WA (2006) Docking interactions in protein kinase and phosphatase networks. Curr Opin Struct Biol 16: 676–685 17079133 10.1016/j.sbi.2006.10.008

[msb156269-bib-0093] Rybakowska I , Romaszko P , Zabielska M , Turyn J , Kaletha K , Barton PJ , Slominska EM , Smolenski RT (2014) Effect of AMP‐deaminase 3 knock‐out in mice on enzyme activity in heart and other organs. Nucleosides, Nucleotides Nucleic Acids 33: 319–322 24940686 10.1080/15257770.2014.880481

[msb156269-bib-0094] Sabio G , Kennedy NJ , Cavanagh‐Kyros J , Jung DY , Ko HJ , Ong H , Barrett T , Kim JK , Davis RJ (2010) Role of muscle c‐Jun NH2‐terminal kinase 1 in obesity‐induced insulin resistance. Mol Cell Biol 30: 106–115 19841069 10.1128/MCB.01162-09 PMC2798290

[msb156269-bib-0095] Sánchez IE , Beltrao P , Stricher F , Schymkowitz J , Ferkinghoff‐Borg J , Rousseau F , Serrano L (2008) Genome‐wide prediction of SH2 domain targets using structural information and the FoldX algorithm. PLoS Comput Biol 4: e1000052 18389064 10.1371/journal.pcbi.1000052 PMC2271153

[msb156269-bib-0096] Schäffer AA , Aravind L , Madden TL , Shavirin S , Spouge JL , Wolf YI , Koonin EV , Altschul SF (2001) Improving the accuracy of PSI‐BLAST protein database searches with composition‐based statistics and other refinements. Nucleic Acids Res 29: 2994–3005 11452024 10.1093/nar/29.14.2994 PMC55814

[msb156269-bib-0097] Schymkowitz J , Borg J , Stricher F , Nys R , Rousseau F , Serrano L (2005) The FoldX web server: an online force field. Nucleic Acids Res 33: W382–W388 15980494 10.1093/nar/gki387 PMC1160148

[msb156269-bib-0098] Sharrocks AD , Yang SH , Galanis A (2000) Docking domains and substrate‐specificity determination for MAP kinases. Trends Biochem Sci 25: 448–453 10973059 10.1016/s0968-0004(00)01627-3

[msb156269-bib-0102] Tanoue T , Adachi M , Moriguchi T , Nishida E (2000) A conserved docking motif in MAP kinases common to substrates, activators and regulators. Nat Cell Biol 2: 110–116 10655591 10.1038/35000065

[msb156269-bib-0103] Tanoue T , Maeda R , Adachi M , Nishida E (2001) Identification of a docking groove on ERK and p38 MAP kinases that regulates the specificity of docking interactions. EMBO J 20: 466–479 11157753 10.1093/emboj/20.3.466 PMC133461

[msb156269-bib-0104] Taru H , Suzuki T (2004) Facilitation of stress‐induced phosphorylation of beta‐amyloid precursor protein family members by X11‐like/Mint2 protein. J Biol Chem 279: 21628–21636 14970211 10.1074/jbc.M312007200

[msb156269-bib-0105] Thomsen MCF , Nielsen M (2012) Seq2Logo: a method for construction and visualization of amino acid binding motifs and sequence profiles including sequence weighting, pseudo counts and two‐sided representation of amino acid enrichment and depletion. Nucleic Acids Res 40: W281–W287 22638583 10.1093/nar/gks469 PMC3394285

[msb156269-bib-0106] Tompa P , Davey NE , Gibson TJ , Babu MM (2014) A million peptide motifs for the molecular biologist. Mol Cell 55: 161–169 25038412 10.1016/j.molcel.2014.05.032

[msb156269-bib-0107] Tonikian R , Zhang Y , Sazinsky SL , Currell B , Yeh J‐H , Reva B , Held HA , Appleton BA , Evangelista M , Wu Y , Xin X , Chan AC , Seshagiri S , Lasky LA , Sander C , Boone C , Bader GD , Sidhu SS (2008) A specificity map for the PDZ domain family. PLoS Biol 6: e239 18828675 10.1371/journal.pbio.0060239 PMC2553845

[msb156269-bib-0108] Vitari AC , Thastrup J , Rafiqi FH , Deak M , Morrice NA , Karlsson HKR , Alessi DR (2006) Functional interactions of the SPAK/OSR1 kinases with their upstream activator WNK1 and downstream substrate NKCC1. Biochem J 397: 223–231 16669787 10.1042/BJ20060220 PMC1479760

[msb156269-bib-0110] Whisenant TC , Ho DT , Benz RW , Rogers JS , Kaake RM , Gordon EA , Huang L , Baldi P , Bardwell L (2010) Computational prediction and experimental verification of new MAP kinase docking sites and substrates including Gli transcription factors. PLoS Comput Biol 6: e1000908 20865152 10.1371/journal.pcbi.1000908 PMC2928751

[msb156269-bib-0111] Wolf A , Eulenfeld R , Bongartz H , Hessenkemper W , Simister PC , Lievens S , Tavernier J , Feller SM , Schaper F (2015) MAPK‐induced Gab1 translocation to the plasma membrane depends on a regulated intramolecular switch. Cell Signal 27: 340–352 25460044 10.1016/j.cellsig.2014.11.017

[msb156269-bib-0112] Yang SH , Galanis A , Sharrocks AD (1999) Targeting of p38 mitogen‐activated protein kinases to MEF2 transcription factors. Mol Cell Biol 19: 4028–4038 10330143 10.1128/mcb.19.6.4028 PMC104362

[msb156269-bib-0114] Ying CY , Dominguez‐Sola D , Fabi M , Lorenz IC , Hussein S , Bansal M , Califano A , Pasqualucci L , Basso K , Dalla‐Favera R (2013) MEF2B mutations lead to deregulated expression of the oncogene BCL6 in diffuse large B cell lymphoma. Nat Immunol 14: 1084–1092 23974956 10.1038/ni.2688 PMC3954820

[msb156269-bib-0115] Zhang J , Zhou B , Zheng C , Zhang Z (2003) A bipartite mechanism for ERK2 recognition by its cognate regulators and substrates. J Biol Chem 278: 29901–29912 12754209 10.1074/jbc.M303909200

[msb156269-bib-0116] Zhang YY , Wu JW , Wang ZX (2011) A Distinct Interaction Mode Revealed by the Crystal Structure of the Kinase p38α with the MAPK Binding Domain of the Phosphatase MKP5. Sci Signal 4: ra88 22375048 10.1126/scisignal.2002241

[msb156269-bib-0117] Zhou T , Sun L , Humphreys J , Goldsmith EJ (2006) Docking interactions induce exposure of activation loop in the MAP kinase ERK2. Structure 14: 1011–1019 16765894 10.1016/j.str.2006.04.006

