# Supplementary material for: Systematic discovery of linear binding motifs targeting an ancient protein interaction surface on MAP kinases
Source: Mol Syst Biol. 2015 Nov 4;11(11):837. doi: 10.15252/msb.20156269 (PMC4670726; doi:10.15252/msb.20156269)
Supplement: Supplementary file 1 — Appendix [file MSB-11-837-s001.pdf]

Appendix to *Systematic discovery of linear binding motifs targeting an ancient protein interaction surface on MAP kinases*

András Zeke, Tomas Bastys, Anita Alexa, Ágnes Garai, Bálint Mészáros, Klára Kirsch, Zsuzsanna Dosztányi, Olga V. Kalinina and Attila Reményi

1 Appendix Figures S1-11

Figure S1. Additional dot-blot experiments (not shown on Figure 3)

Figure S2. Summary on the definitions of different D-motif classes

Figure S3. In vitro protein-peptide binding affinity assays

Figure S4. Further bimolecular fragment complementation (BiFC) experiments

Figure S5. Major functions of JNK1 and other MAPKs based on docking motif distribution

Figure S6. Comparisons of best 100 hits for JIP1, NFAT4 and greater MEF2A type motifs

Figure S7. Mechanisms of docking motif emergence

Figure S8. In vitro kinase assays on AAKG2 and DCX

Figure S9. Evolutionary sequence conservation of D-motifs and phosphorylation target motifs

Figure S10. Independent emergence of multiple D-motifs in a single protein

Figure S11. Role of the D-motif in GAB1 in the context of the EGF/Ras→ERK signaling cascade

2 Appendix Table S1. FoldX templates used per motif subtypes

3 Appendix References

A

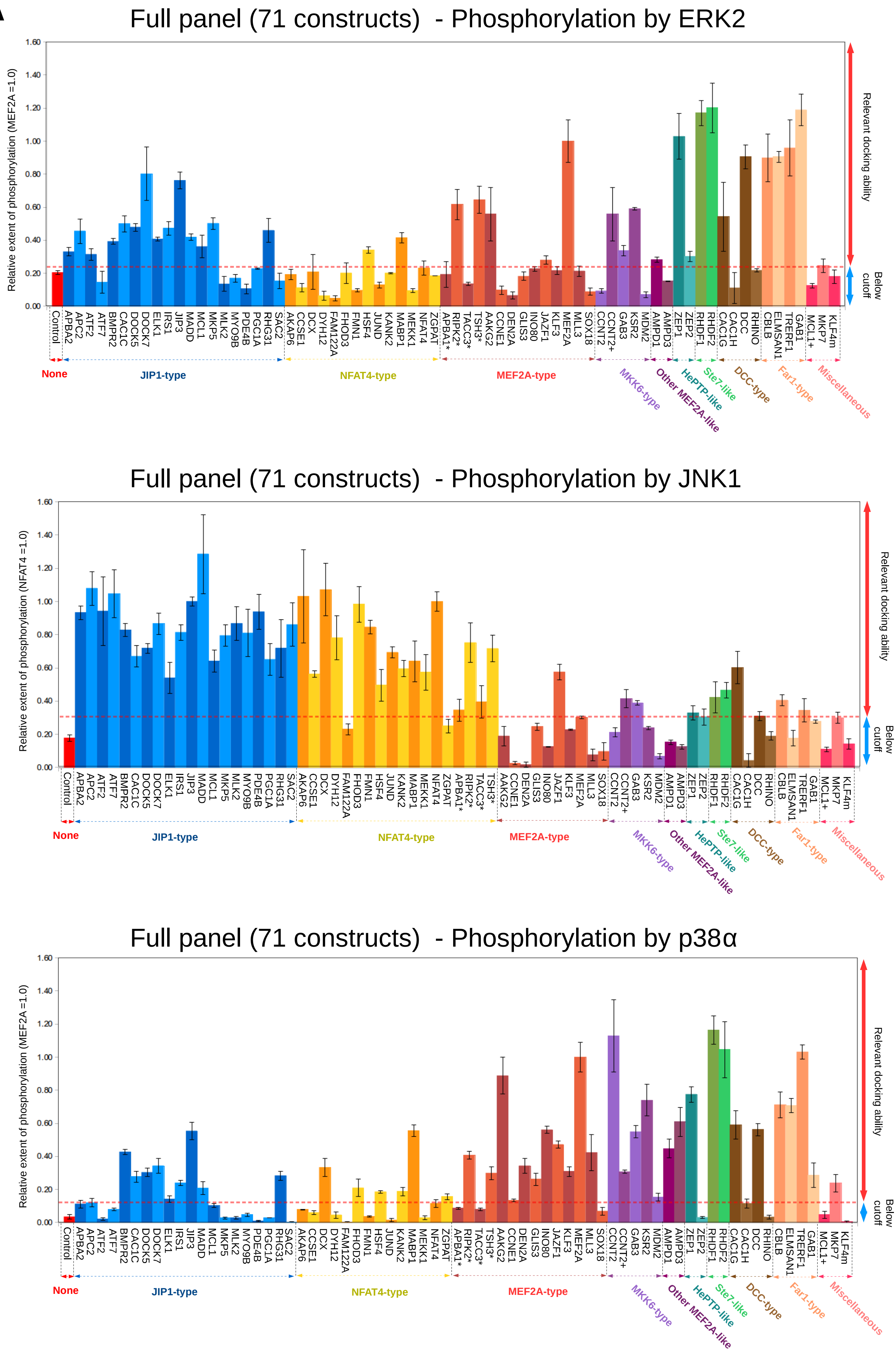
Full panel (71 constructs) - Phosphorylation by ERK2
Relative extent of phosphorylation (MEF2A =1.0)
Relevant docking ability
Below cutoff
Control APBA2 APC2 ATF2 ATF7 BMPR2 CAC1C DOCK5 DOCK7 ELK1 IRS1 JIP3 MADD MCL1 MKP5 MLK2 MYO9B PDE4B PGC1A RHG31 SAC2 AKAP6 CCSE1 DCX DYH12 FAM122A FHOD3 FMN1 HSF4 JUND KANK2 MABP1 MEKK1 NFAT4 ZGPAT APBA1* RIPK2* TACC3* TSH3* AAKG2 CCNE1 DEN2A GLIS3 INO80 JAZF1 KLF3 MEF2A MLL3 SOX18 CCNT2 CCNT2+ GAB3 KSR2 MDM2 AMPD1 AMPD3 ZEP1 ZEP2 RHDF1 RHDF2 CAC1G CAC1H DCC RHINO CBLB ELMSAN1 TRERF1 GAB1 MCL1+ MKP7 KLF4m
None
JIP1-type
NFAT4-type
MEF2A-type
MKK6-type
Other MEF2A-like
HePTP-like
Ste7-like
DCC-type
Far1-type
Miscellaneous
Full panel (71 constructs) - Phosphorylation by JNK1
Relative extent of phosphorylation (NFAT4 =1.0)
Relevant docking ability
Below cutoff
Full panel (71 constructs) - Phosphorylation by p38α
Relative extent of phosphorylation (MEF2A =1.0)
Relevant docking ability
Below cutoff

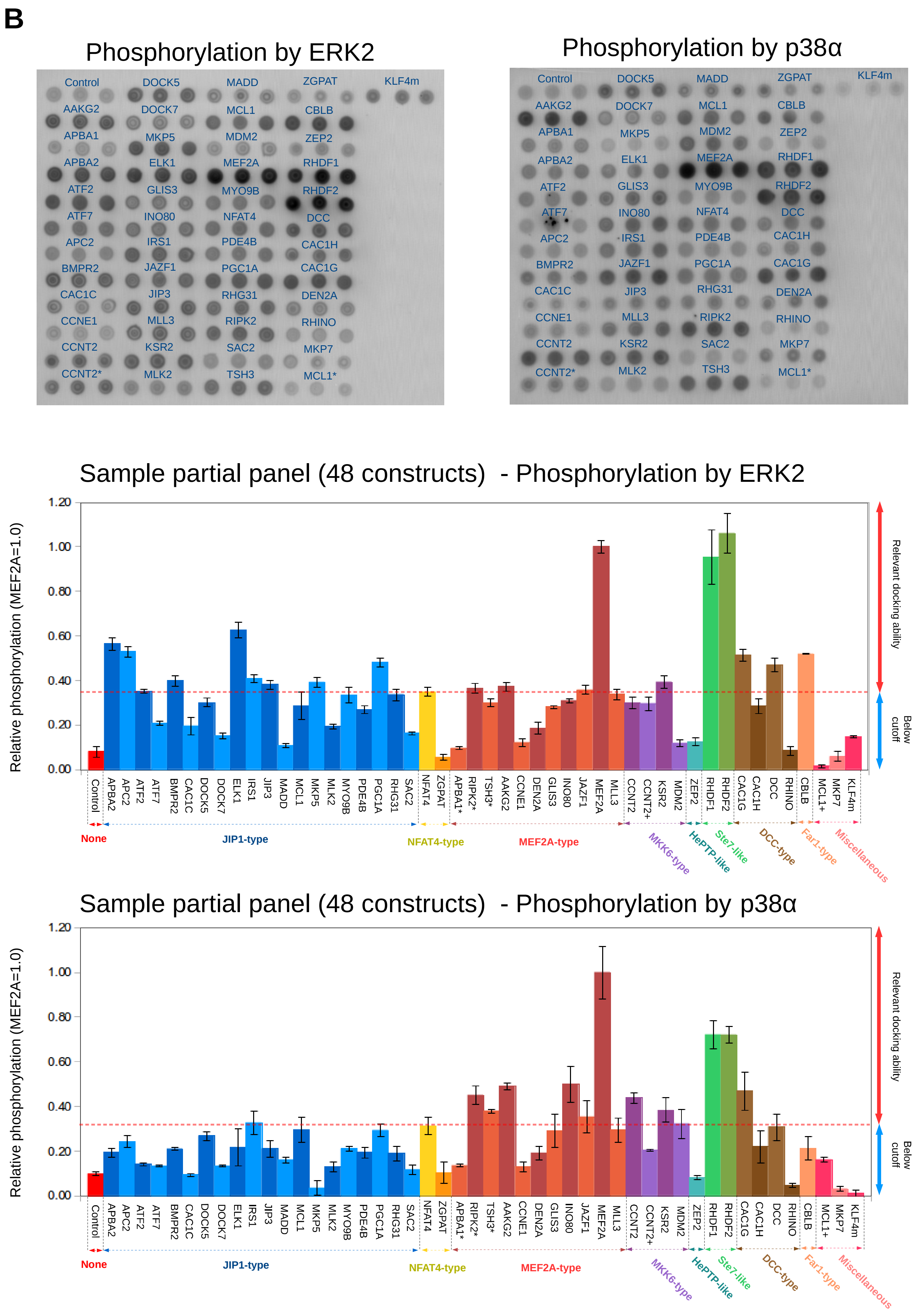
B
Phosphorylation by ERK2
Phosphorylation by p38α
Control
DOCK5
MADD
ZGPAT
KLF4m
AAKG2
DOCK7
MCL1
CBLB
APBA1
MKP5
MDM2
ZEP2
APBA2
ELK1
MEF2A
RHDF1
ATF2
GLIS3
MYO9B
RHDF2
ATF7
INO80
NFAT4
DCC
APC2
IRS1
PDE4B
CAC1H
BMPR2
JAZF1
PGC1A
CAC1G
CAC1C
JIP3
RHG31
DEN2A
CCNE1
MLL3
RIPK2
RHINO
CCNT2
KSR2
SAC2
MKP7
CCNT2*
MLK2
TSH3
MCL1*
Sample partial panel (48 constructs) - Phosphorylation by ERK2
Sample partial panel (48 constructs) - Phosphorylation by p38α
Relative phosphorylation (MEF2A=1.0)
Relevant docking ability
Below cutoff
None
JIP1-type
NFAT4-type
MEF2A-type
MKK6-type
HePTP-like
Ste7-like
DCC-type
Far1-type
Miscellaneous

Figure S1. Additional dot-blot experiments (not shown on Figure 3)

(**A**) Results of dot-blot experiments (71 constructs). The cutoff (red dashed line) is set to the level of well-established non-cognate controls (NFAT4 in case of JNK1, and MEF2A in the case of ERK2 and p38α). (**B**) Summary of the 48 construct experiment for ERK2 and p38α, which complements the 48 construct experiment with JNK1 shown on Figure 3. Data shown on Panel A,B or on C was generated by using different stocks of proteins and different western-blot signal detection techniques (chemiluminescence or fluorescence). *: dual class motifs (e.g. NFAT4 and MEF2A); +: second motif in protein (non-overlapping with the first); m: murine (non-human) motif sequence. Error bars refer to SD based on triplicates.

JIP1-type (JIP1 class)

Initial (**loose**): [RK]-P-[^P]-[^P]-[LIV]-X-[LIVMFP]

Refined (**tight**): [RK]-P-[^P]-[^P]-L-X-[LIVMF]

NFAT4-type (NFAT4 class)

Initial (**loose**): [RK]-X-[^P]-[LIVM]-X-[LIV]-X-[LIVMFP]

Refined (**tight**): [RK]-[^P]-[^P]-[LIM]-X-L-X-[LIVMF]

MEF2A-type (greater MEF2A class)

Loose motif: [RK]-[RK]-X-X-[LIVMP]-X-[LIV]-X-[LIVMFP]

Tight motif: [RK]-[RK]-X-X-[LIVMP]-X-[LIV]-X-[LIVMF]

MKK6-type (greater MEF2A class)

Loose motif: [RK]-X-[RK]-X-X-X-[LIVMP]-X-[LIV]-X-[LIVMFP]

Tight motif: [RK]-X-[RK]-X-X-X-[LIVMP]-X-[LIV]-X-[LIVMF]

MEF2A/MKK6-like (greater MEF2A class)

Loose motif: [RK]-$(X)_{2-4}$-[LIVMP]-X-[LIV]-X-[LIVMFP]

All greater MEF2A class

Refined consensus (common / all types): [RK]-X(2-4)-[LIVMP]-X-[LIV]-X-[LIVMF]

All greater DCC class

Initial consensus (all subtypes): [RK]-X(2-4)-[LIVP]-X-X-[LIV]-X-[LIVMFP]

DCC-type (greater DCC class)

Tight motif: [RK]-X(2-4)-[LIVP]-P-X-[LIV]-X-[LIVMF]

Far1-type (greater DCC class)

Tight motif: [RK]-X(2-4)-[LIVP]-X-P-[LIV]-X-[LIVMF]

Miscellaneous (loose pseudo-motifs)

[RK]-X(variable)-[hydrophobic]-X(1-2)-[hydrophobic]-X-[hydrophobic]

Ste7-type/tight (Greater HePTP class)

[LIV]-X-[RK]-[RK]-X(4)-[LIVMP]-X-[LIV]-X-[LIVMFP]

HePTP-type/tight (Greater HePTP class)

[LIV]-[^P]-[^P]-[RK]-[RK]-G-X(4)-[LIVMP]-X-[LIV]-X-[LIVMFP]

Figure S2. Summary on the definitions of different D-motif classes

Positively charged and hydrophobic core amino acids are colored blue and red, respectively, while special amino acids at intervening (x) positions are colored in purple (i.e. proline or glycine in the DCC or in the HePTP classes, respectively).
+: These proteins had more than one, non-overlapping MAPK-binding elements tested (names with/without "+" refer to different sequences).
*: The sequence of these constructs satisfies at least two different, overlapping consensus motifs (thus they are featured under more than one motif class/subclass). For the sake of simplicity, not all combinations are shown.
~ : This novel isoform is found in NCBI and other databases but not in UniProt.
m: This sequence refers to the murine instead of the human protein. MAPK docking was suggested in mouse Klf4 earlier but this was not confirmed here (Kim *et al*, 2012).

ERK2 **JNK1** **p38α**

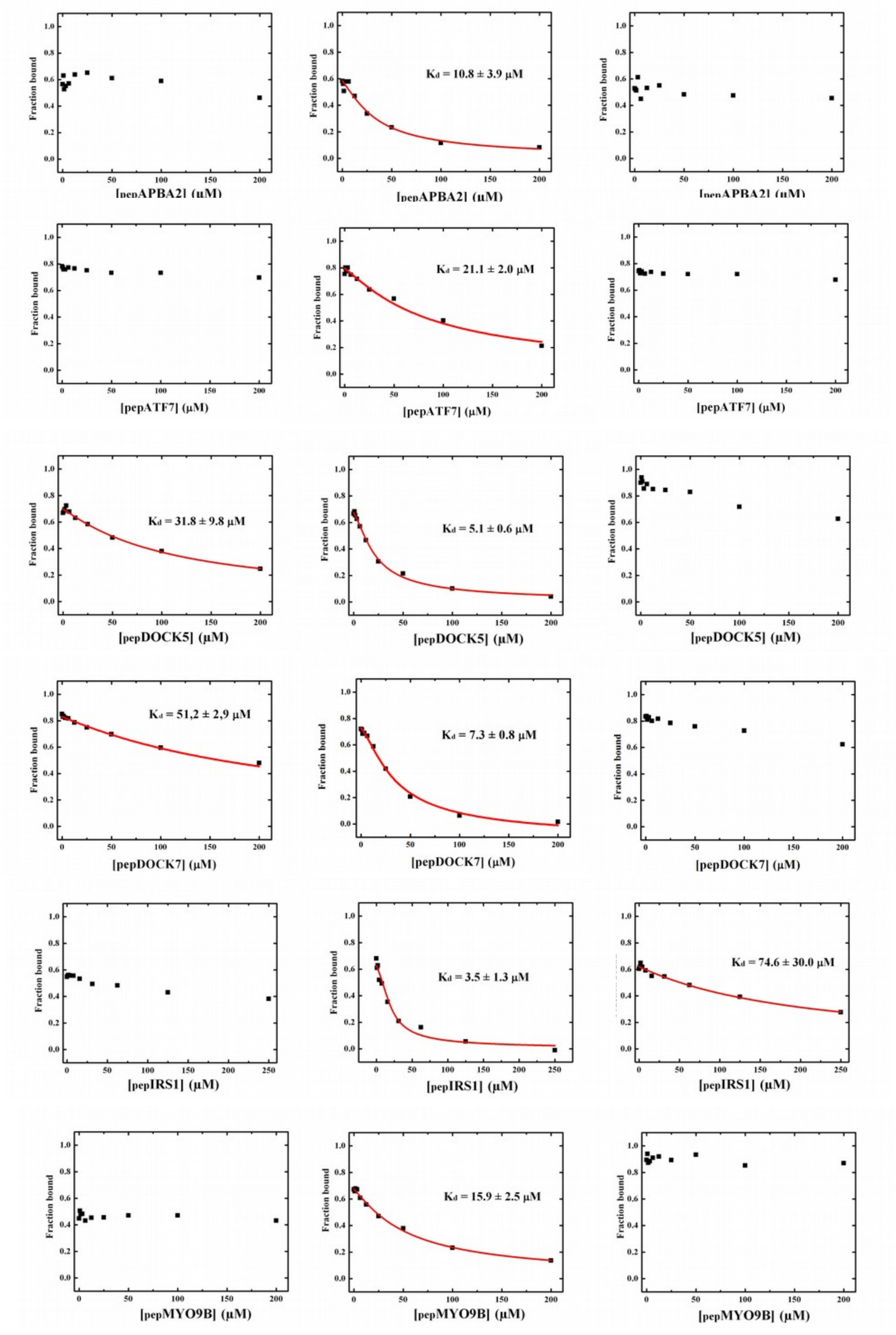

ERK2 **JNK1** **p38α**

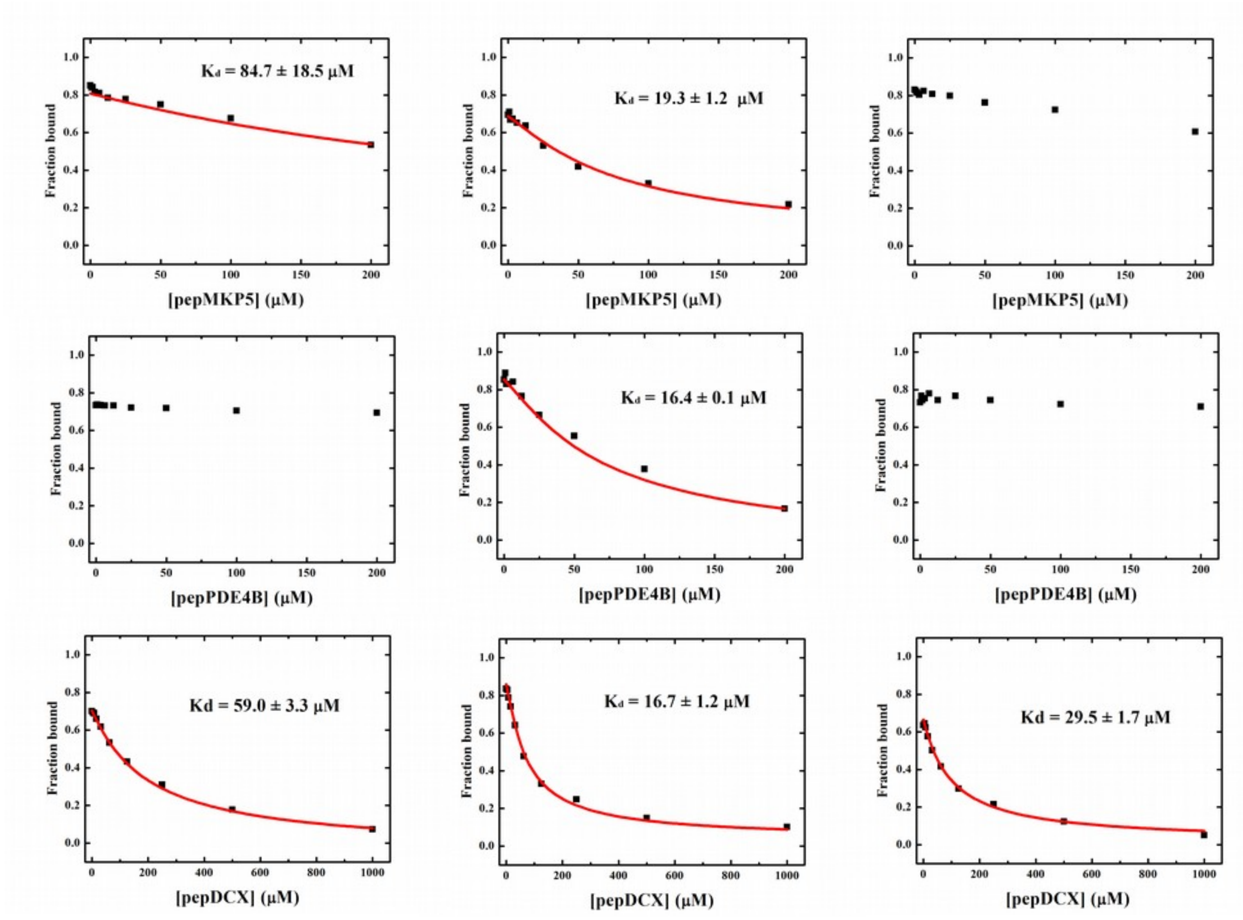

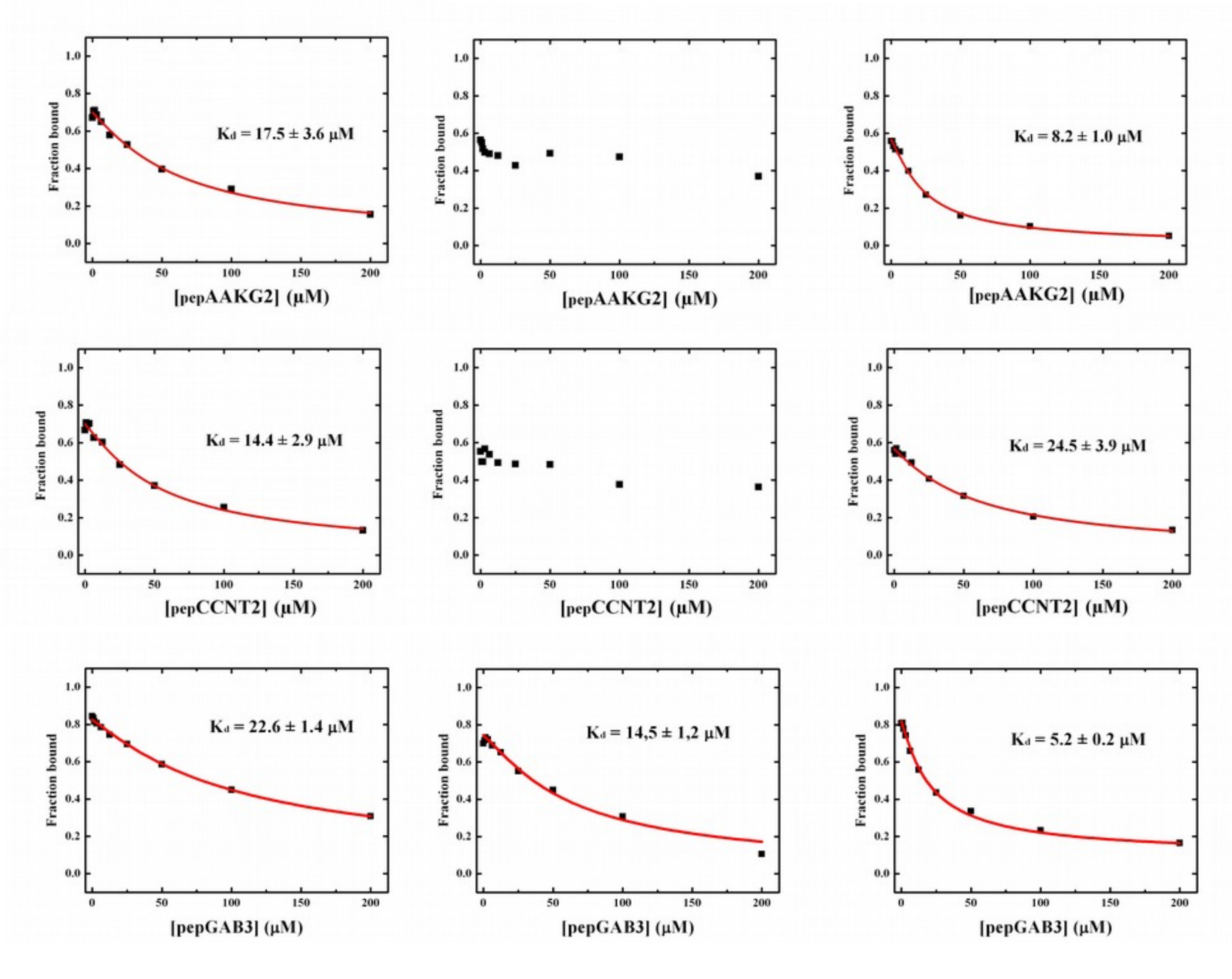

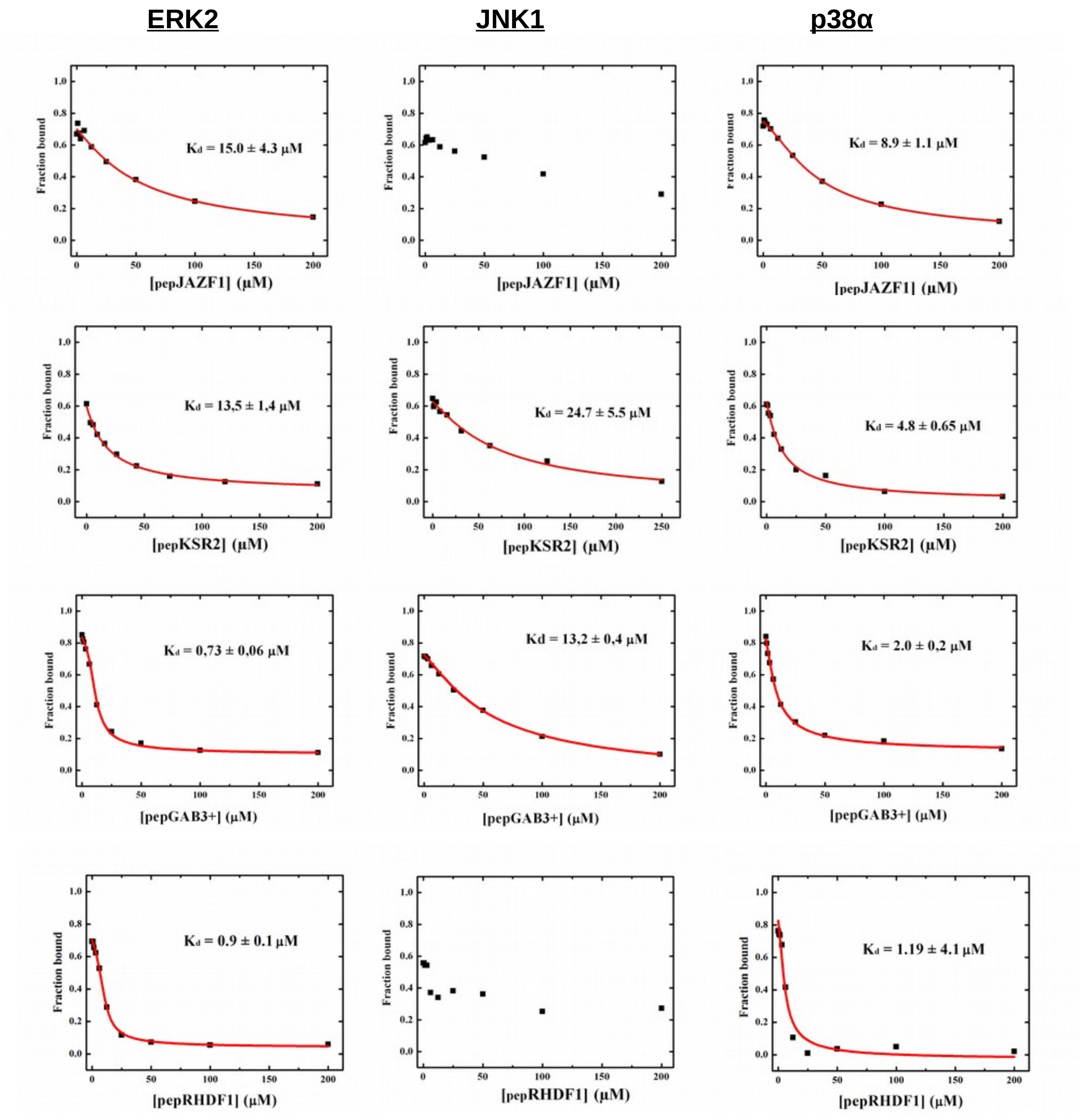

Figure S3. Protein-peptide binding affinity assays

Titration curves for each competitive titration against fluorescently labeled known positive control peptides.

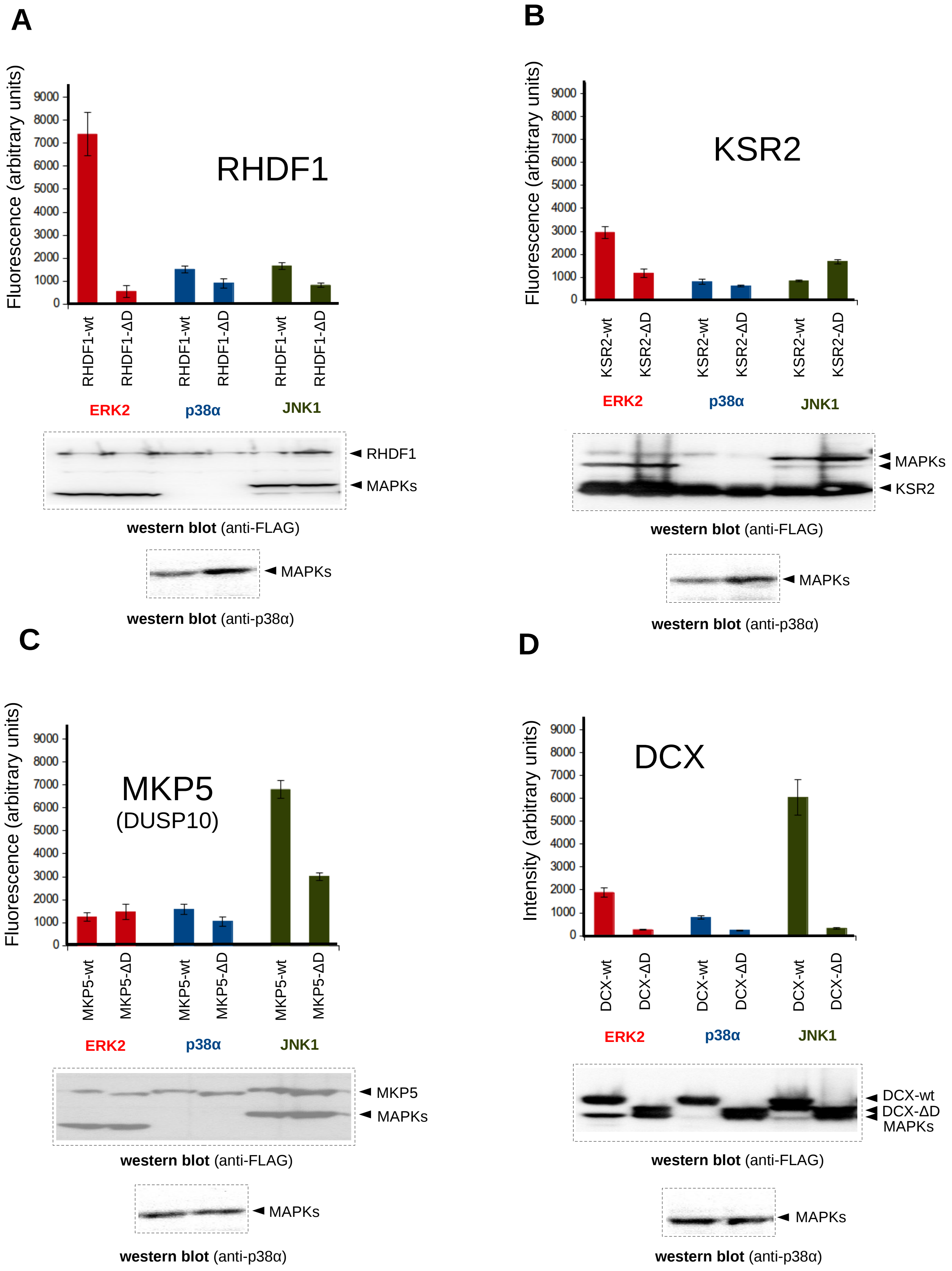
A
Fluorescence (arbitrary units)
9000
8000
7000
6000
5000
4000
3000
2000
1000
0
RHDF1
RHDF1-wt
RHDF1-ΔD
RHDF1-wt
RHDF1-ΔD
RHDF1-wt
RHDF1-ΔD
ERK2
p38α
JNK1
RHDF1
MAPKs
western blot (anti-FLAG)
MAPKs
western blot (anti-p38α)
B
Fluorescence (arbitrary units)
9000
8000
7000
6000
5000
4000
3000
2000
1000
0
KSR2
KSR2-wt
KSR2-ΔD
KSR2-wt
KSR2-ΔD
KSR2-wt
KSR2-ΔD
ERK2
p38α
JNK1
MAPKs
KSR2
western blot (anti-FLAG)
MAPKs
western blot (anti-p38α)
C
Fluorescence (arbitrary units)
9000
8000
7000
6000
5000
4000
3000
2000
1000
0
MKP5
(DUSP10)
MKP5-wt
MKP5-ΔD
MKP5-wt
MKP5-ΔD
MKP5-wt
MKP5-ΔD
ERK2
p38α
JNK1
MKP5
MAPKs
western blot (anti-FLAG)
MAPKs
western blot (anti-p38α)
D
Intensity (arbitrary units)
9000
8000
7000
6000
5000
4000
3000
2000
1000
0
DCX
DCX-wt
DCX-ΔD
DCX-wt
DCX-ΔD
DCX-wt
DCX-ΔD
ERK2
p38α
JNK1
DCX-wt
DCX-ΔD
MAPKs
western blot (anti-FLAG)
MAPKs
western blot (anti-p38α)

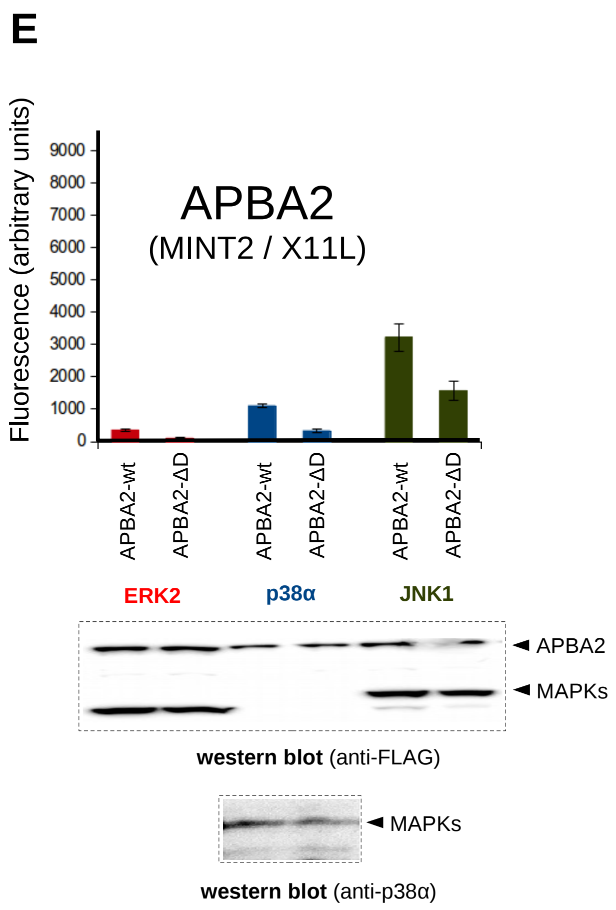

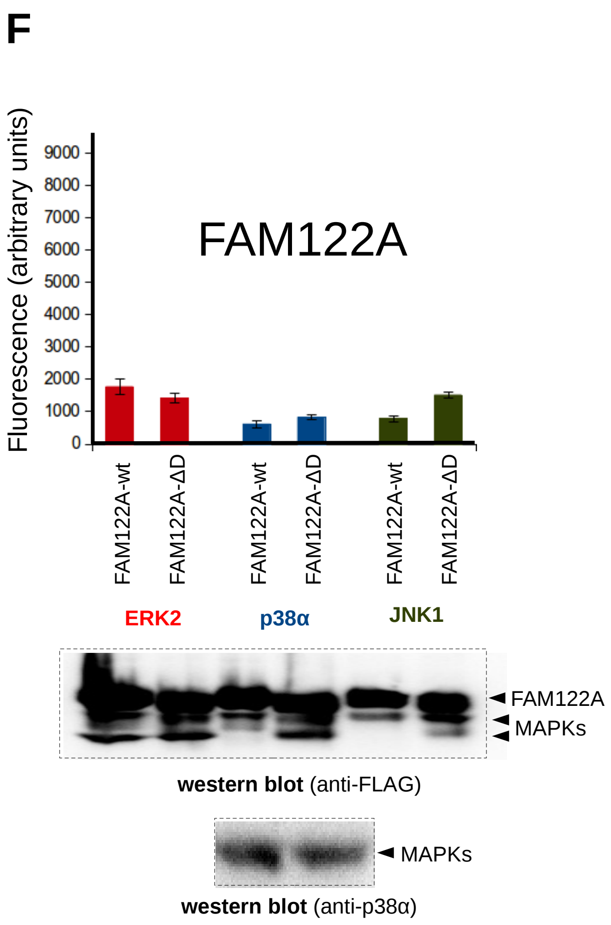

Figure S4. Further bimolecular fragment complementation (BiFC) experiments

Results of BiFC experiments (in pairs of wild-type and D-motif mutant proteins) are shown for RHDF1 (iRhom1), MKP5 (DUSP10), KSR2, DCX, APBA2 (Mint2) and FAM122A proteins. Each construct was tested against all three MAPKs. Histograms refer to fluorescence readings on a plate-reader from 4 to 6 independent wells. To prove that interacting protein as well as MAPK concentrations were comparable for each wild type/mutant pair, expression levels were monitored with western blots on cell lysates. Interacting proteins, ERK2 and JNK1 levels were tested with anti-FLAG blots, while p38α was detected with anti-p38α. A reduction in fluorescence shows if the mutated or deleted motif plays a role in MAPK recruitment or not. We only considered intensity changes where the fluorescence obtained for the wild-type construct was well above the baseline so that to separate relevant effects from the background. All fluorescence differences (versus the wild-type protein) indicated on Figure 4B were found to be significant ($p<0.05$) with a two-tailed Student's test (not indicated separately on the histograms). In the case of KSR2, the full-length protein could not be expressed to detectable levels, therefore experiments were performed on the "KSR2-unique" insert only (the sequence is indicated in Appendix Fig S7C). In the case of DCX, all experiments were performed on a specific splice isoform of the human protein (corresponding to the main DCX isoform in mice) that contains a canonical NFAT4-type motif.

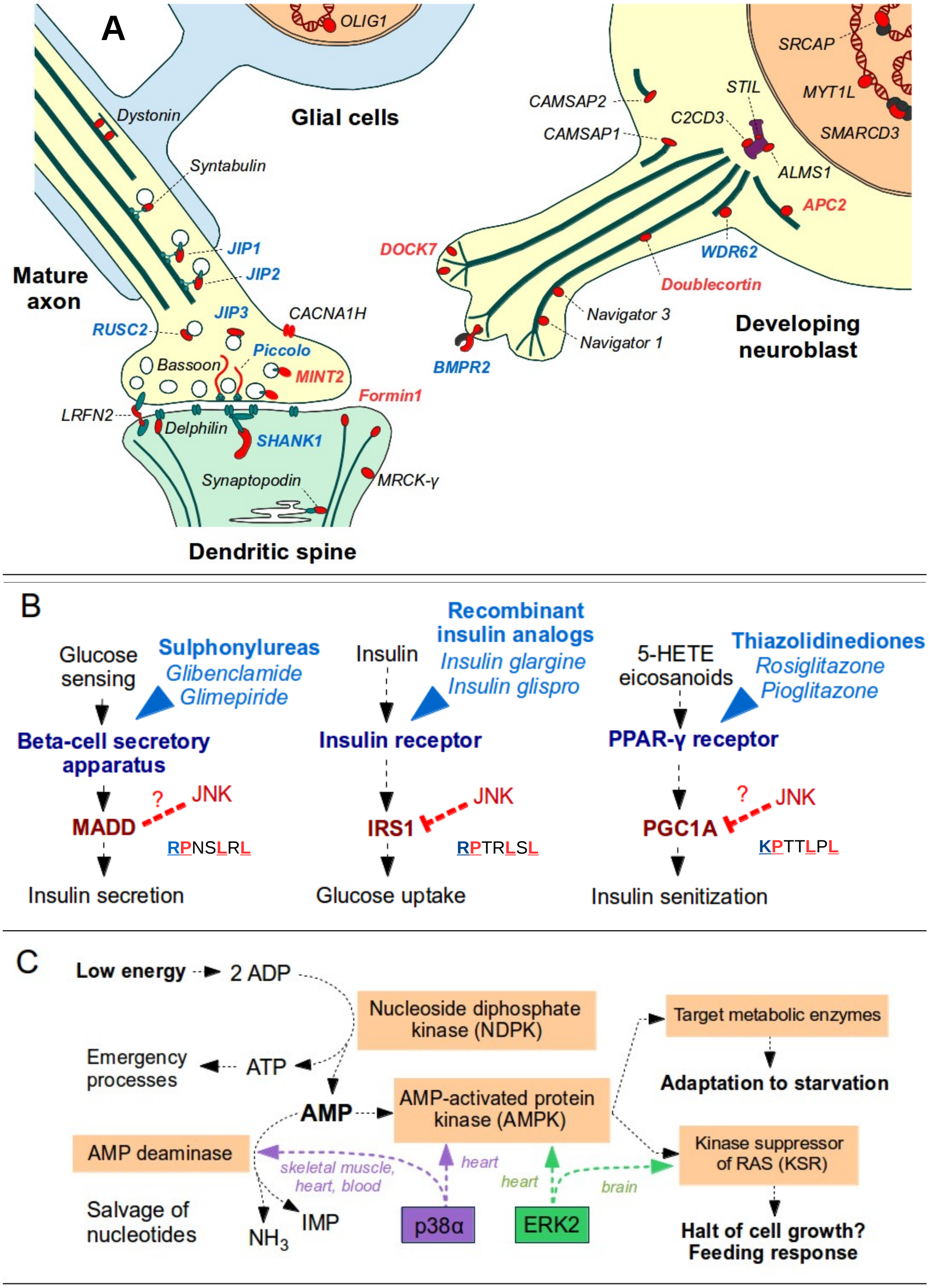
A
OLIG1
Glial cells
Dystonin
Syntabulin
JIP1
JIP2
JIP3
Mature axon
RUSC2
CACNA1H
Piccolo
Bassoon
MINT2
Formin1
LRFN2
Delphilin
SHANK1
Synaptopodin
MRCK-γ
Dendritic spine
SRCAP
MYT1L
SMARCD3
STIL
C2CD3
ALMS1
CAMSAP2
CAMSAP1
APC2
WDR62
DOCK7
Doublecortin
Navigator 3
Navigator 1
BMPR2
Developing neuroblast
B
Glucose sensing
Sulphonylureas
Glibenclamide
Glimepiride
Beta-cell secretory apparatus
JNK
MADD
RPNSLRL
Insulin secretion
Insulin
Recombinant insulin analogs
Insulin glargine
Insulin glispro
Insulin receptor
JNK
IRS1
RPTRLSL
Glucose uptake
5-HETE eicosanoids
Thiazolidinediones
Rosiglitazone
Pioglitazone
PPAR-γ receptor
JNK
PGC1A
KPTTLPL
Insulin senitization
C
Low energy
2 ADP
Nucleoside diphosphate kinase (NDPK)
Emergency processes
ATP
AMP
AMP-activated protein kinase (AMPK)
Target metabolic enzymes
Adaptation to starvation
AMP deaminase
skeletal muscle, heart, blood
heart
heart
brain
Kinase suppressor of RAS (KSR)
Salvage of nucleotides
NH3
IMP
p38α
ERK2
Halt of cell growth?
Feeding response

Figure S5. Major functions of JNK1 and other MAPKs based on docking motif distribution

(A) Dedicated JNK-interacting proteins are frequently found in neurons of the central nervous system. Many of these proteins play critical roles in the the differentiation of neuroblasts (right) as well as in the maintenance of mature synapses (left) (Yau *et al*, 2014; Heydet *et al*, 2013; Lu *et al*, 2001; Lamba *et al*, 2008; Kim *et al*, 1997; Bogoyevitch *et al*, 2012; Gdalyahu *et al*, 2004; Watabe-Uchida *et al*, 2006; Simon-Areces *et al*, 2011; Deller *et al*, 2003; Dajas-Bailador *et al*, 2008; Okamoto & Südhof, 1997; Whisenant *et al*, 2010; Chen *et al*, 2014; Podkowa *et al*, 2010; Cai *et al*, 2007; Sun *et al*, 2013; Bayer *et al*, 2005). Blue letters denote the already-known JNK docking partners, while those containing newly-identified D-motifs are written in red. Names in black letters refer to predictions only - based on the presence of high-ranking JIP1 or NFAT4-type docking motifs in them.
(B) JNK plays a pathological role in type II diabetes. The three insets show three critical steps in insulin action - all targets of important anti-diabetic drugs. Each of these regulatory pathways signal through key proteins containing JIP1-type docking motifs. These critical points are either known or predicted to be negatively regulated by JNK itself. This phenomenon is true to the regulation of insulin secretion (left) controlled by MADD (also known as IG20, a susceptibility gene for type II diabetes), the core insulin signaling pathway (middle) containing IRS1 (a well-known JNK substrate) and the pathway regulating insulin sensitivity (right), passing through PGC1A (PPAR-γ receptor coactivator 1α, known to be regulated by MAPK phosphorylation-dependent ubiquinylation) (Li *et al*, 2014; Lee *et al*, 2003; Finck & Kelly, 2006; Olson *et al*, 2008).
(C) The starvation-activated AMPK pathway connects to p38 and/or ERK1/2 signaling in specialized tissues. The regulatory and enzymatic processes elicited by AMP release are shown on the pathway diagram (dotted lines). The key enzyme, AMP-activated protein kinase (AMPK) is phosphorylating a variety of metabolic enzymes and transcription factors (Mihaylova & Shaw, 2011). Kinase suppressor of RAS (KSR) proteins are also substrates of AMPK. The broadly expressed KSR1 is responsible for the control of growth factor pathways (Shen *et al*, 2013). The other paralog, KSR2, is expressed predominantly in the brain and ample evidence suggests that it controls energy expenditure and feeding behavior (Costanzo-Garvey *et al*, 2009). Lastly, excess AMP is partly eliminated by various adenosine monophosphate deaminase (AMPD) enzymes, depending on the tissue studied (Rybakowska *et al*, 2014). p38α (magenta) was found to interact with the cardiac-specific AMPK subunit γ2 (AAKG2) and with docking motifs discovered in AMP deaminase 1 and 3 enzymes. ERK2 (green) also interacts with AAKG2 but was also found to bind to KSR2. Since most of these proteins have a restricted expression pattern, the interactions likely do not exist in most cells, only in a few dedicated tissue types (written on the lines).

A

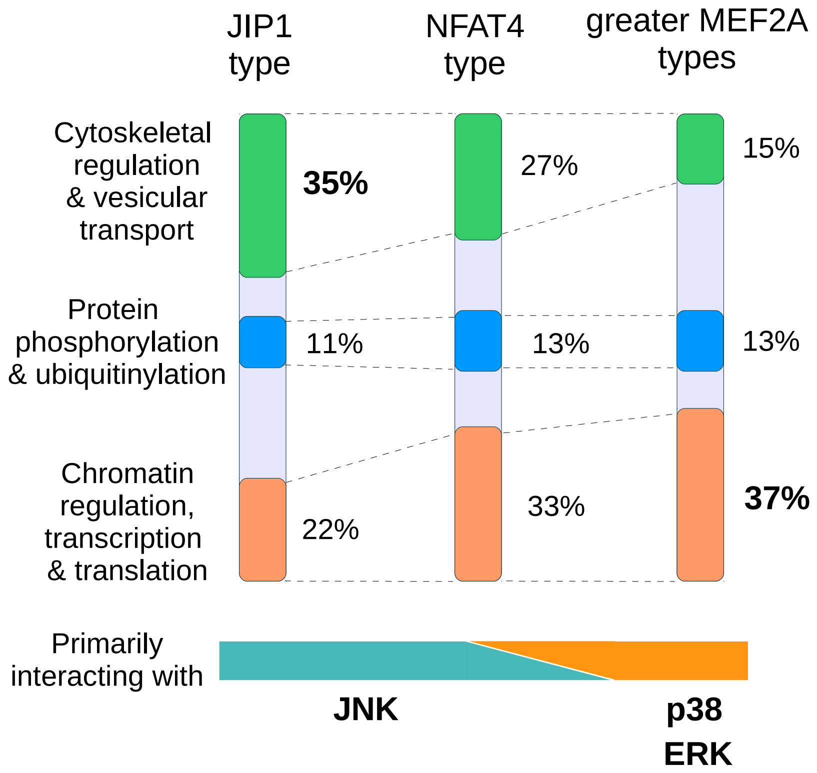

B

Family	Function	Protein with JIP1-like element	Protein with NFAT4-like element
Formin	Actin filament assembly	**FMNL1**	**FMN1**<br>**FHOD3**
Smoothelin	Actin binding	**SMTL2**	**SMTN**
HECT-WW	E3 ubiquitin ligase	**HECW1**	**HECW2**
ATF/CREB	Transcription factor	**ATF7**	**ATF7**

C

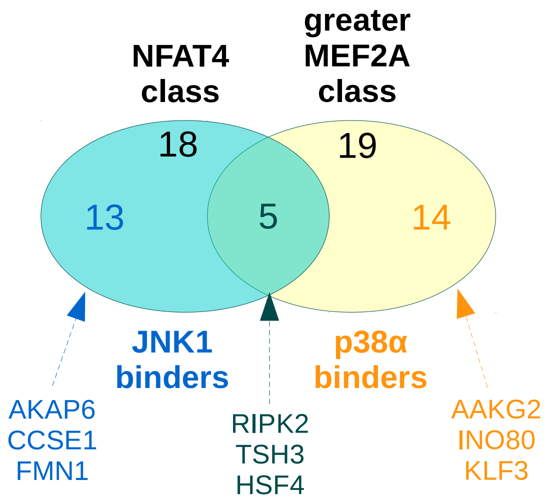

Figure S6. Comparisons of best 100 hits for JIP1, NFAT4 and greater MEF2A type motifs

(A) Comparison of functional super-grouping of hits among the best JIP1, NFAT4 or greater MEF2A type motifs. Within the JIP1-type motif bearing proteins (JNK1 partners), cytoskeletal and associated functions dominate, while those with a greater MEF2A-type motif (p38α and ERK2 interactors) mostly tend to be implicated in the regulation of gene expression. Those with an NFAT4-type motif (mostly JNK1 interactors) show a distribution in-between the former two. Note that the number of proteins involved in protein phosphorylation or ubiquitinylation are similar across all three classes. The percentages refer to the number of different proteins among the best 100 motif hits.
(B) A few selected examples of protein families in which one member carries a JIP1-type motif, but another closely related member has an NFAT4-type motif instead. (The latter ones are typically found in a different position than the JIP1 motif, thus likely evolved independently in all cases.) We only considered examples in which both motifs are either high-ranking predictions or were experimentally validated at least on a fragment level.
(C) Experimental proof for the suggested overlap between JNK1 and p38α partners. The definition of NFAT4 (JNK-binding) and greater MEF2A (primarily p38α-binding) are similar, resulting in a sizable set of "dual-class" motifs. Dot-blot experiments show that these dual-class peptides often bind both MAPKs with comparable affinities. The upper numbers in this diagram represent motifs (of either NFAT4 or greater MEF2A types) that were tested as positive in the dot-blot arrays. The lower row of numbers - on the other hand - shows the exact numbers that selectively interacted with JNK1 (blue), p38α (yellow) or with both (green). For each case, a few characteristic examples are also written below.

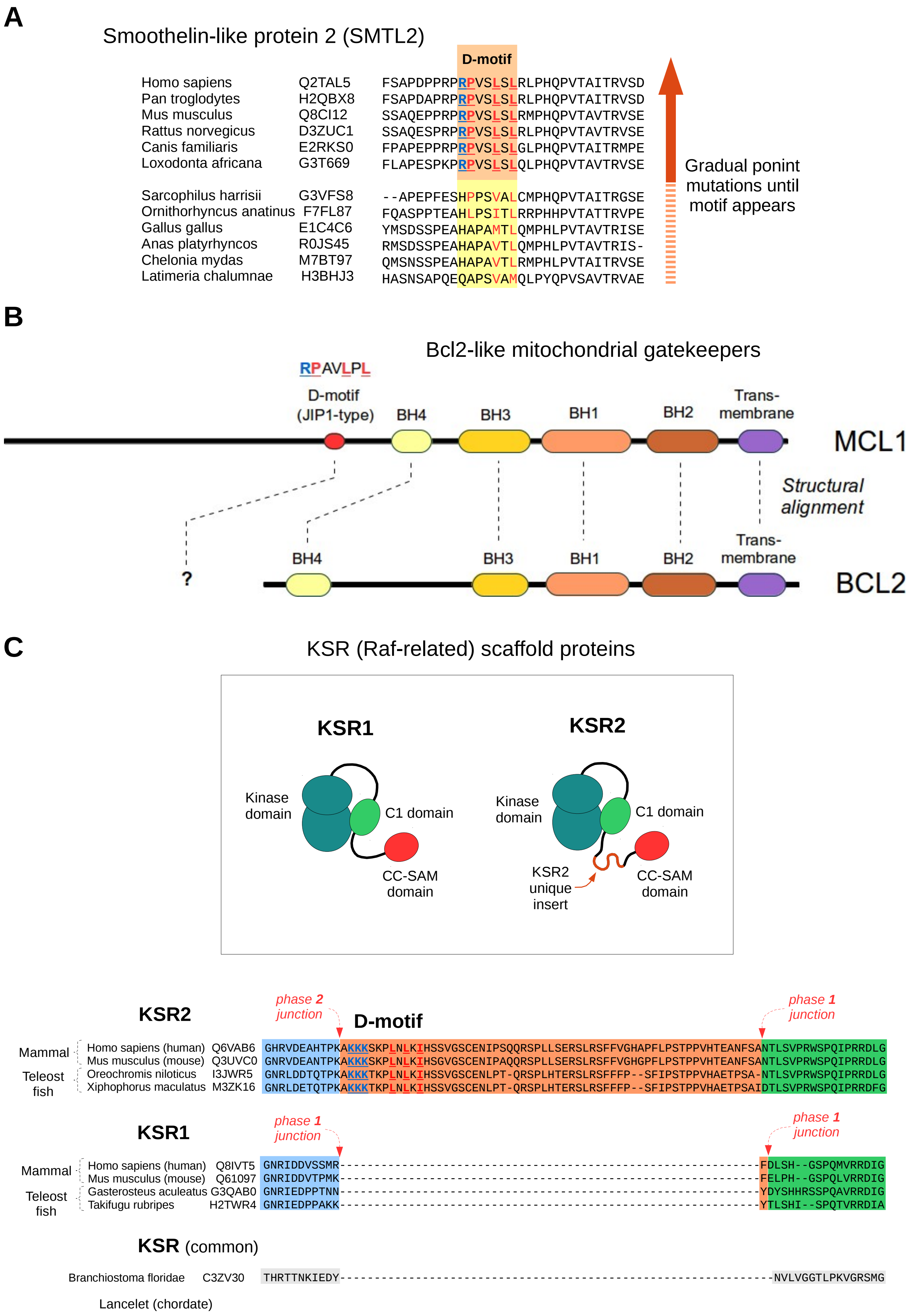
A
Smoothelin-like protein 2 (SMTL2)
D-motif
Homo sapiens Q2TAL5 FSAPDPPRPRPVSLSLRLPHQPVTAITRVSD
Pan troglodytes H2QBX8 FSAPDAPRPRPVSLSLRLPHQPVTAITRVSD
Mus musculus Q8CI12 SSAQEPPRPRPVSLSLRMPHQPVTAVTRVSE
Rattus norvegicus D3ZUC1 SSAQESPRPRPVSLSLRLPHQPVTAVTRVSE
Canis familiaris E2RKS0 FPAPEPPRPRPVSLSLGLPHQPVTAITRMPE
Loxodonta africana G3T669 FLAPESPKPRPVSLSLQLPHQPVTAVTRVSE
Sarcophilus harrisii G3VFS8 --APEPFESHPPSVALCMPHQPVTAITRGSE
Ornithorhyncus anatinus F7FL87 FQASPPTEAHLPSITLRRPHHPVTATTRVPE
Gallus gallus E1C4C6 YMSDSSPEAHAPAMTLQMPHLPVTAVTRISE
Anas platyrhyncos R0JS45 RMSDSSPEAHAPAVTLQMPHLPVTAVTRIS-
Chelonia mydas M7BT97 QMSNSSPEAHAPAVTLRMPHLPVTAITRVSE
Latimeria chalumnae H3BHJ3 HASNSAPQEQAPSVAMQLPYQPVSAVTRVAE
Gradual ponint mutations until motif appears
B
Bcl2-like mitochondrial gatekeepers
RPAVLPL
D-motif (JIP1-type)
BH4
BH3
BH1
BH2
Trans-membrane
MCL1
Structural alignment
?
BH4
BH3
BH1
BH2
Trans-membrane
BCL2
C
KSR (Raf-related) scaffold proteins
KSR1
Kinase domain
C1 domain
CC-SAM domain
KSR2
Kinase domain
C1 domain
KSR2 unique insert
CC-SAM domain
KSR2
phase 2 junction
D-motif
phase 1 junction
Mammal
Homo sapiens (human) Q6VAB6 GHRVDEAHTPKAKKKSKPLNLKIHSSVGSCENIPSQQRSPLLSERSLRSFFVGHAPFLPSTPPVHTEANFSANTLSVPRWSPQIPRRDLG
Mus musculus (mouse) Q3UVC0 GNRVDEANTPKAKKKSKPLNLKIHSGVGSCENIPAQQRSPLLSERSLRSFFVGHGPFLPSTPPVHTEANFSANTLSVPRWSPQIPRRDLG
Teleost fish
Oreochromis niloticus I3JWR5 GNRLDDTQTPKAKKKTKPLNLKIHSSVGSCENLPT-QRSPLHTERSLRSFFFP--SFIPSTPPVHAETPSA-NTLSVPRWSPQIPRRDLG
Xiphophorus maculatus M3ZK16 GNRLDETQTPKAKKKTKPLNLKIHSSVGSCENLPT-QRSPLHTERSLRSFFFP--SFIPSTPPVHAETPSAIDTLSVPRWSPQIPRRDFG
KSR1
phase 1 junction
phase 1 junction
Mammal
Homo sapiens (human) Q8IVT5 GNRIDDVSSMR
FDLSH--GSPQMVRRDIG
Mus musculus (mouse) Q61097 GNRIDDVTPMK
FELPH--GSPQLVRRDIG
Teleost fish
Gasterosteus aculeatus G3QAB0 GNRIEDPPTNN
YDYSHHRSSPQAVRRDIG
Takifugu rubripes H2TWR4 GNRIEDPPAKK
YTLSHI--SPQTVRRDIA
KSR (common)
Branchiostoma floridae C3ZV30 THRTTNKIEDY
NVLVGGTLPKVGRSMG
Lancelet (chordate)

D

PDE4 family of cAMP phosphodiestherases

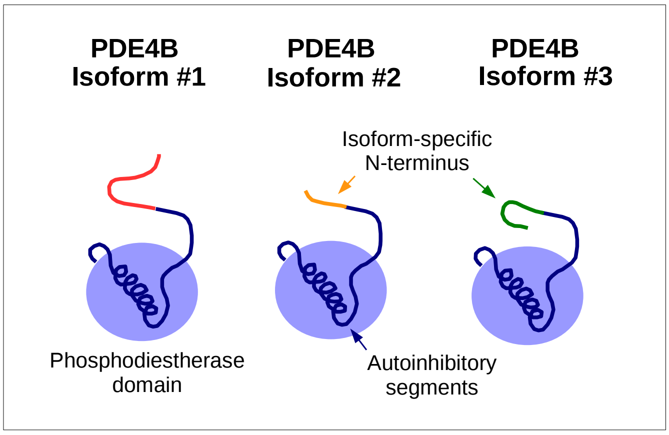


```
                                              D-motif
H. sapiens
PDE4A  isoform  #7    53   EPS--DPGVPLPPRPTTLPLLIPPRISITRAENDSFEAENGPTPSPGRSPLDSQASPG-- 108
PDE4B  isoform  #1    61   ERAR-TPEGDGISRPTTLPLTTLPSIAITTVSQECFDVENGP--SPGRSPLDPQASSSAG 117
PDE4D  isoform #11   57   EQADLKSESENIQRPTSLPLKILPLIAITSAESSGFDVDNGT--SAGRSPLDPMTSPGSG 114
                           *  :         ***:***   * *:** ....  *:.:**   * ******  :*  .

                                              D-motif
D. rerio
PDE4B   E7FH06       109  ----RSPDDEFMARPTTLPFITPPRIDITPVDTECFDVENGP--SASCSPLDPQASPGSG 166
PDE4D   F1Q5E8        58  YCERRPETETVQPRPTSLPLRVPPLIAITSADTSSFDVDNGT--SSGRSPLDPMASPGSG 115
                              *    : .  ***:**: .** * ** .**..***:**   *:. ***** ******
```

E

MKP5 (DUSP10) phosphatase

```
                                                       D-motif
Homo sapiens (human)                Q9Y6W6   RVVVALSRPVRPQDLNLCLDSSYLGSA   } JIP1-type
Danio rerio (zebrafish)             F1QIT6   RIVVALPRPIRPQELRLRLDTSYLETT   }

Crassostrea gigas (pacific oyster)  K1QUW9   TLTTDILPCRRRLGLRLDFGADDYTCV   }
Tribolium castaneum (flour beetle)  D6W7T6   -MSVCLESSRPRDGLKLPLNRSFSEPG   } NFAT4-type
Strigonium maritimum (centipede)    T1ITQ4   ---MLSVSESRRESLKLPLNRSLSEPG   }
```

CCSER1 microtubule-binding protein

```
                                                       D-motif
Homo sapiens (human)           Q9C0I3   KREEPEFPEPSKQNLSLKLTKDVDQEARCS   } NFAT4-type
Mus musculus (mouse)           Q8C0C4   MLEEPKCPEPSKQNLSLRITKDTDQEARCS   }

Danio rerio (zebrafish)        B0UYT1   GRDPREPFFREQRPMSLCLSRDDEAVPGLE   } JIP1-type
Ictalurus punctatus (catfish)  W5UFS8   TAKECRDNNREPRPVSLCVSCDDELFSGLE   }
```

F

ELK transcription factors

```
Homo sapiens ELK1      P19419   SPEISQPQKGRKPRDLELPLSPSLLGGP   }
Homo sapiens ELK3      P41970   TKSPSLPPKAKKPKGLEISAPPLVLSGT   }
Homo sapiens ELK4      P28324   KDKVNNSSRSKKPKGLELAPTLVITSSD   } JIP1-type
Strongylocentrotus purpuratus                                  }
  (sea urchin)   Q95UJ1         LVTGSSLGNKPKPLQLSVPSTIKGSESS   }

Crassostrea gigas (pacific oyster)       K1R3J9   HKMSSSSTTRPKPTPLSLPIHDPSPLPT   } Far1-type
Caenorhabditis briggase (roundworm)      A8XJ60   LSAPPPSKKGMKPNPLNLTGTTDFTLAP   }
```

TAB1 signalling adaptor

```
Homo sapiens (human)     Q15750   GRVYPVSVPYSSAQSTSKTSVTLSLVMPSQGQMVNGA   } MEF2A-like (atypical,
Danio rerio (zebrafish)  Q6NUU7   GRIYPVSVPYSNHQSTSKTSVTLSLVMPAQGTLTNGS   } N-terminally extended)

Branchiostoma floridae (lancelet)   C3Z725   TQGGVYNPVSVPYRPHKNEGPPPKLIMPPKQPVPTNL   } DCC-like  (atypical,
Crassostrea gigas (pacific oyster)  K1PQD5   PTAGGAYHPVSVPFYSPRPNLPPTVTIPTLSTAERVE   } N-terminally extended)
```

G

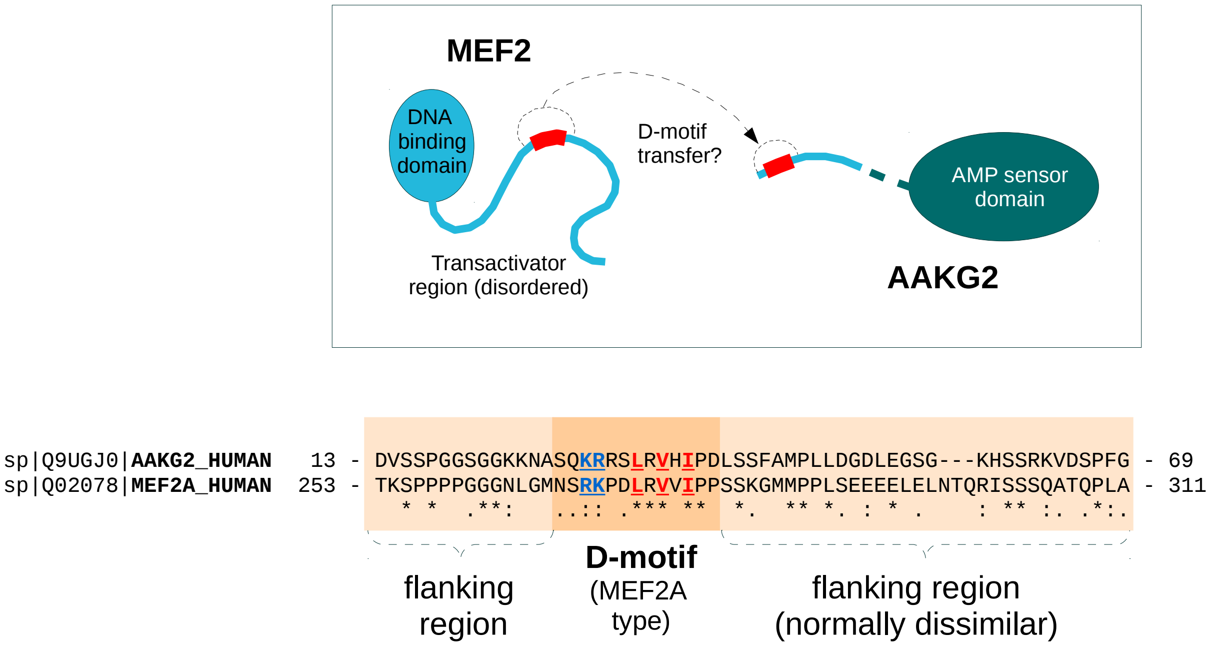

MEF2
DNA binding domain
D-motif transfer?
AMP sensor domain
Transactivator region (disordered)
AAKG2
sp|Q9UGJ0|AAKG2_HUMAN 13 - DVSSPGGSGGKKNASQKRRSLRVHIPDLSSFAMPLLDGDLEGSG---KHSSRKVDSPFG - 69
sp|Q02078|MEF2A_HUMAN 253 - TKSPPPPGGGNLGMNSRKPDLRVVIPPSSKGMMPPLSEEEELELNTQRISSSQATQPLA - 311
flanking region
D-motif
(MEF2A type)
flanking region (normally dissimilar)

H

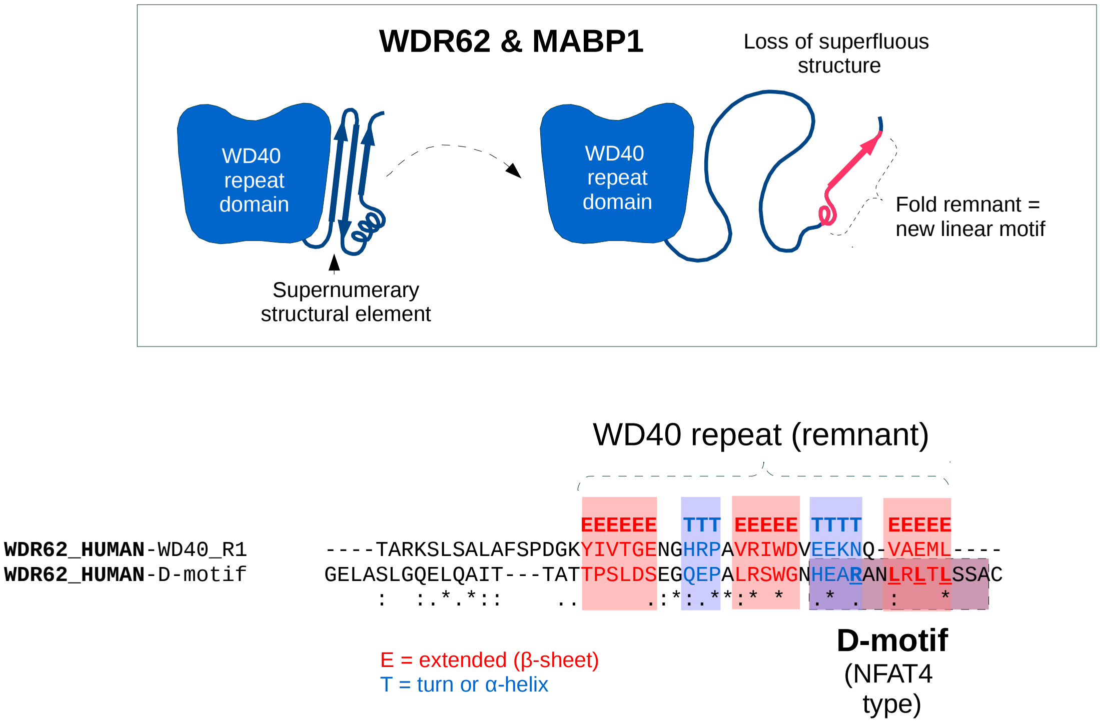

WDR62 & MABP1
Loss of superfluous structure
WD40 repeat domain
WD40 repeat domain
Supernumerary structural element
Fold remnant = new linear motif
WD40 repeat (remnant)
EEEEEE TTT EEEEE TTTT EEEEE
WDR62_HUMAN-WD40_R1 ----TARKSLSALAFSPDGKYIVTGENGHRPAVRIWDVEEKNQ-VAEML----
WDR62_HUMAN-D-motif GELASLGQELQAIT---TATTPSLDSEGQEPALRSWGNHEARANLRLTLSSAC
E = extended (β-sheet)
T = turn or α-helix
D-motif
(NFAT4 type)

Figure S7. Mechanisms of docking motif emergence

(A) The majority of docking motifs analyzed in the current study seems to have evolved by random mutations (genetic drift) stumbling upon a suitable consensus sequence. This is exemplified by a known JNK-interactor (smoothelin-like 2, SMTL2) (Gordon *et al*, 2013). Comparison of vertebrate SMTL2 sequences shows great diversity in various animals, with the sequence gradually approximating a JIP1-type consensus in non-eutherian mammals. Eutherian (placental) mammals, on the other hand, show a consistently conserved JIP1-type motif locked in place.
(B) A structural alignment between the mitochondrial gatekeeper protein Mcl1 and its closest relative, Bcl2. The analysis suggests that the enlarged N-terminus of Mcl1 (together with its newly-identified JIP1-type docking motif) has no counterpart in Bcl2 (or any other related gene). This disordered segment might have arisen from non-coding DNA sequences by a translational start point shift.
(C) Comparison of mammalian Kinase suppressor of Ras (KSR) proteins 1 and 2. In contrast to KSR1, KSR2 possesses a unique insert in its N-terminal linker segment between the coiled-coil containing sterile alpha motif domain (CC-SAM) and the phospholipid-binding C1 domain. Comparison of vertebrate sequences with the chordate *Branchistoma floridae* KSR (where only a single KSR gene is found, as it has not undergone the whole genome duplication characteristic of vertebrates) suggests that the KSR2-specific insert is secondary and likely arose by splicing site shifts. While both the 5' and the 3' splice sites correspond to each other in KSR1 and KSR2, this exon of KSR1 is minuscule, encoding only a single amino acid. The KSR2-specifc insert, on the other hand, contains a functional D-motif.
(D) Alternative splicing in the PDE4 family of phosphodietherases can endow each PDE4 enzyme with a unique N-terminus with special regulatory motifs. Furthermore, this inventory of alternative exons is conserved across different PDE4 paralogs. Thus, the D-motif-containing PDE4B isoform #1 corresponds to the isoform #7 of PDE4A and isoform #11 of PDE4D. The same alternative N-termini are also found in distantly-related vertebrates as in zebrafish (*Danio rerio*).
(E) Alignments between vertebrate MKP5 (DUSP10) phosphatases and their relatives in certain protostomes reveal that the D-motif of MKP5 is an ancient heritage. However, the D-motif found in seashells or insects satisfies the NFAT4 consensus, instead of a JIP1-type which is characteristic of vertebrates. Similar docking motif transmutations are sometimes also seen in vertebrates: the cytoskeleton-associated serine-rich coiled-coil protein 1 (CCSER1) possesses an NFAT4-type motif in mammals, while the same segment aligns to a JIP1-type motif in certain bony fishes (e.g. *Danio rerio).*
(F) Further examples for potential D-motif transmutations. The ELK family of transcription factors shows a JIP1-type motif in vertebrates, but their protostome relatives tend to contain a Far1-type docking motif at the same place. This phenomenon is also evolutionarily consistent. The sea urchin ELK protein (a deuterostome, thus closer to human than to *Caenorhabditis*) also satisfies a JIP1-type consensus, rather than a Far1-type. The TNF-receptor complex associated Tab1 adaptor hints at similar subtle evolutionary changes. The vertebrate Tab1 motif is grossly equivalent to a greater MEF2A-type motif, with an extra N-terminal extension. The same extension is also found in invertebrate Tab1 proteins, but there the D-motif core is more consistent with a DCC-type arrangement.
(G) Alignment between D-motif containing MEF2 factors (MEF2A or MEF2C) and the unique N-terminus of AAKG2 reveals an unexpected fit extending to broad regions flanking the D-motifs. Tentatively, the D-motif of AAKG2 might have arisen through an illegitimate recombination with a MEF2 gene or pseudogene.
(H) Linear motifs may also arise from a (disrupted) folded domain. In the MABP1/WDR62 family of JNK interacting proteins the D-motif containing segment on their C-termini might have arisen from duplicated, supernumerary WD40 repeats (unable to fold into the closed solenoid WD40 “wheel”) (Cohen-Katsenelson *et al*, 2011; Bogoyevitch *et al*, 2012; Koyano *et al*, 1999). This is more obvious in WDR62, where the surroundings of the docking motif clearly correspond to a partial WD40 repeat, and even aligns with it in a structural sense (i.e. the extended hydrophobic part of the D-motif aligns with a beta-sheet, while the presumably helical N-terminus aligns with a turn/loop, etc.). In the more divergent paralog MABP1, the sequence has more radically changed and almost no conservation tendencies can be seen outside the D-motif itself.

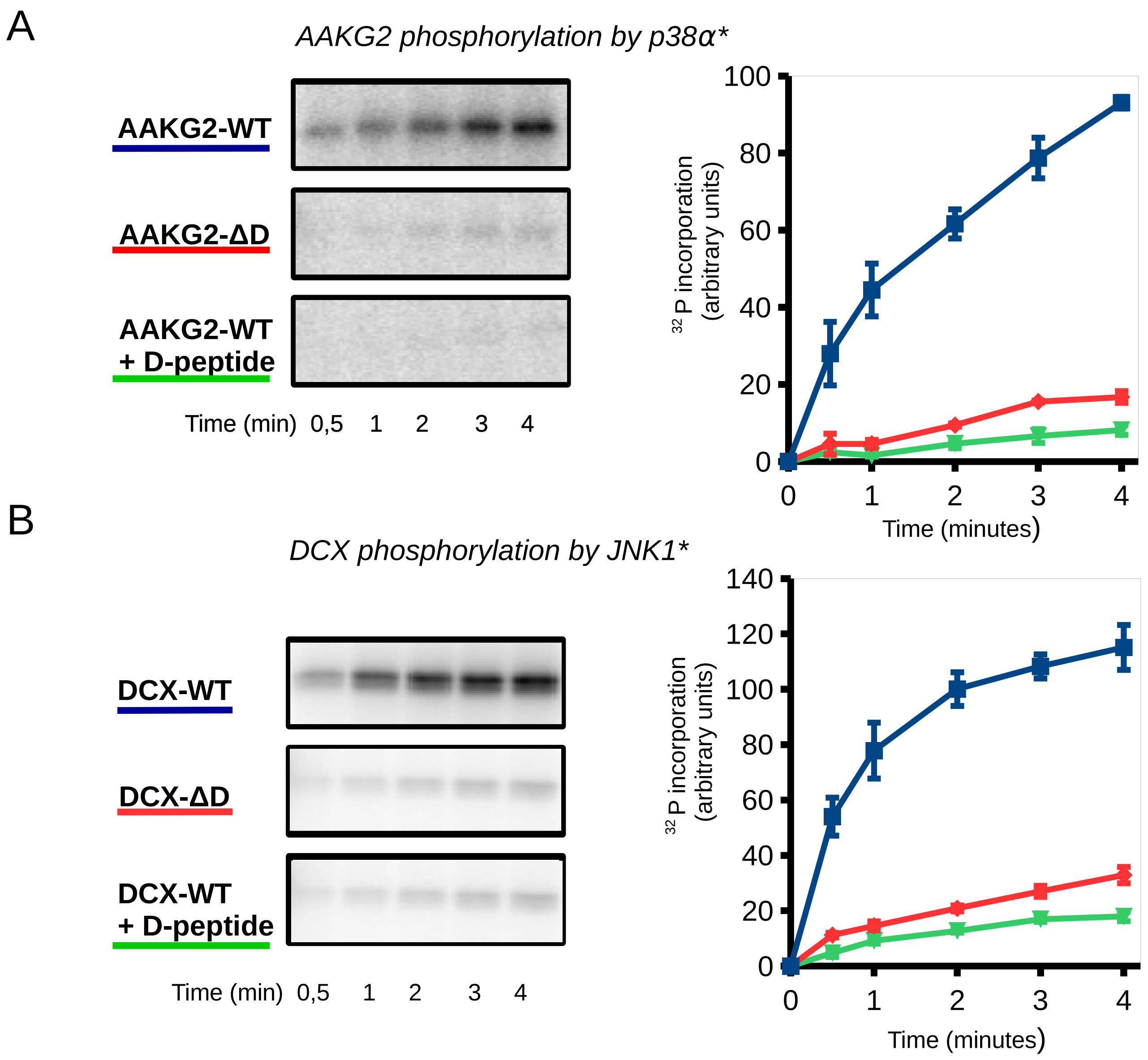

Figure S8. In vitro kinase assays on AAKG2 and DCX

(A) Time profiles of *in vitro* phosphorylation assays (with ^{32}P-labelled ATP) using purified AAKG2 proteins as substrate (WT: wild-type, ΔD: AAKG2 lacking the D-motif, WT+D-peptide: mixture of wild-type AAKG2 with the competitor D-peptide of MK2 which selectively interferes with docking) and activated p38α as the enzyme.
(B) Time profiles of *in vitro* phosphorylation assays (with ^{32}P-labelled ATP) using DCX as substrate (WT: wild-type, ΔD: DCX lacking the D-motif, WT+D-peptide: mixture of wild-type DCX with the competitor D-peptide of JIP1 which selectively interferes with docking) and activated JNK1 as the enzyme. Results of three parallel experiments (N=3) are shown on the graph on the right. Error bars show standard deviations from the mean.

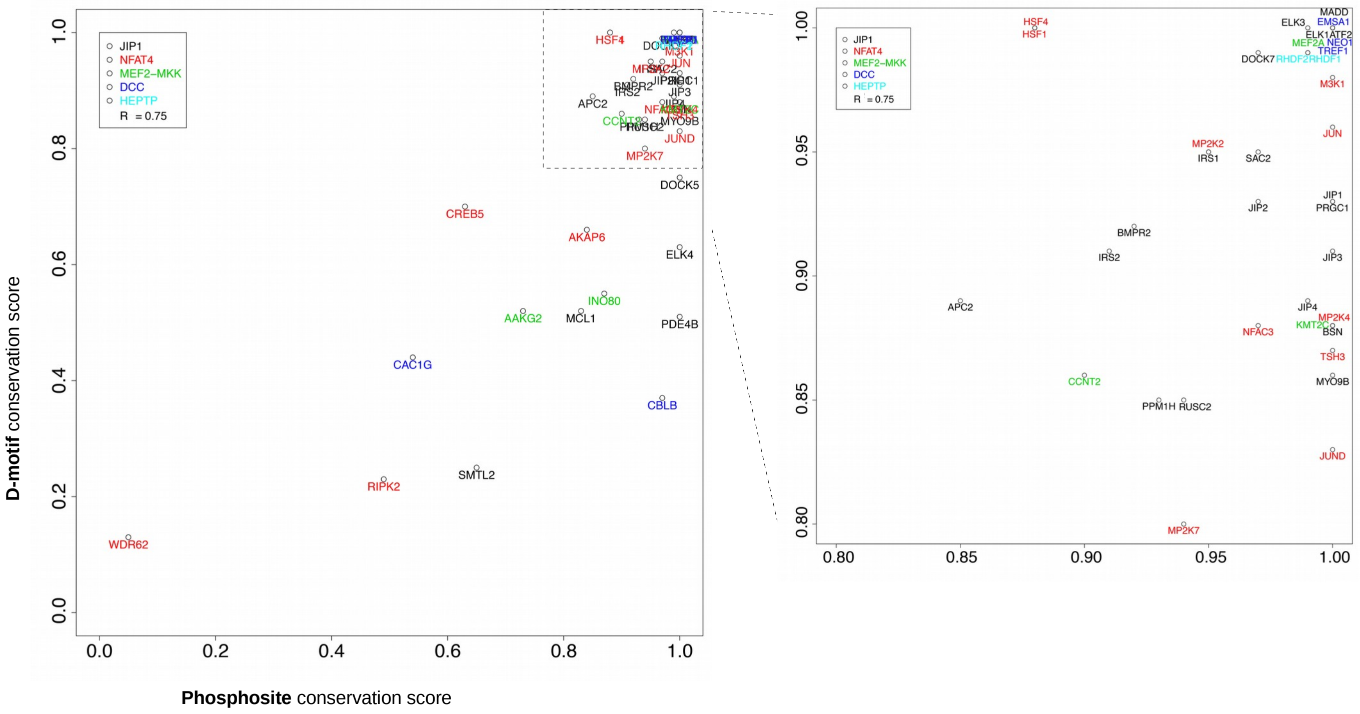

Figure S9. Evolutionary sequence conservation of D-motifs and phosphorylation target motifs

The plots show the evolutionary conservation of the human motifs among vertebrate homologs based on eggNOG vertebrate alignments. The panel on the right shows the upper-right hand side corner of the complete plot on the left. Notice that most proteins lie close to the diagonal indicating that their D-motif and their most conserved putative phosphorylation target motif display similar evolutionary conservation. D-motif or phosphosite conservation score: normalized average motif depth in vertebrate alignments of eggNOG. R: Pearson product-moment correlation coefficient.

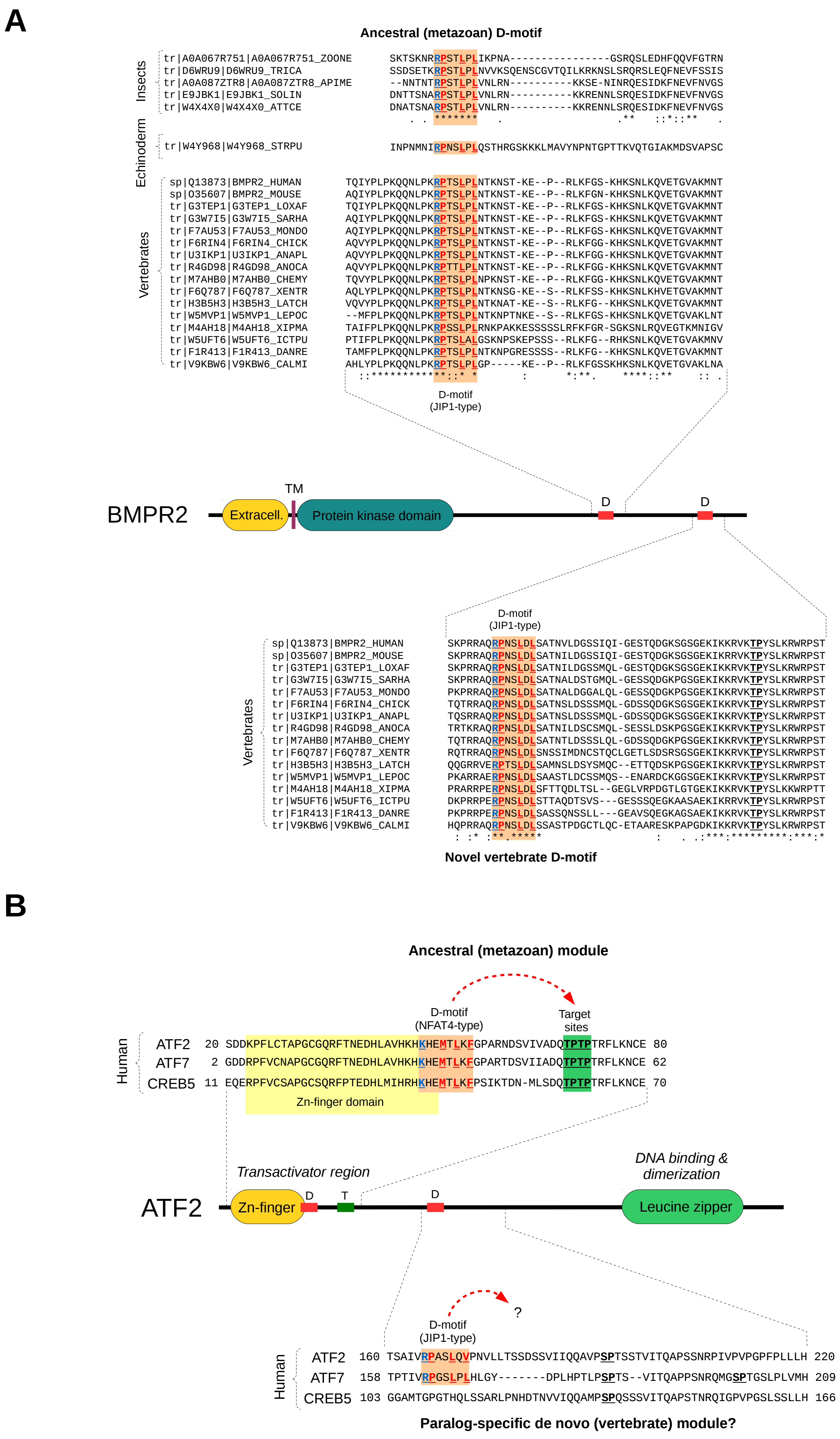
A
Ancestral (metazoan) D-motif
Insects
Echinoderm
Vertebrates
tr|A0A067R751|A0A067R751_ZOONE SKTSKNRRPSTLPLIKPNA---------------GSRQSLEDHFQQVFGTRN
tr|D6WRU9|D6WRU9_TRICA SSDSETKRPSTLPLNVVKSQENSCGVTQILKRKNSLSRQRSLEQFNEVFSSIS
tr|A0A087ZTR8|A0A087ZTR8_APIME --NNTNTRPSTLPLVNLRN----------KKSE-NINRQESIDKFNEVFNVGS
tr|E9JBK1|E9JBK1_SOLIN DNTTSNARPSTLPLVNLRN----------KKRENNLSRQESIDKFNEVFNVGS
tr|W4X4X0|W4X4X0_ATTCE DNATSNARPSTLPLVNLRN----------KKRENNLSRQESIDKFNEVFNVGS
tr|W4Y968|W4Y968_STRPU INPNMNIRPNSLPLQSTHRGSKKKLMAVYNPNTGPTTKVQTGIAKMDSVAPSC
sp|Q13873|BMPR2_HUMAN TQIYPLPKQQNLPKRPTSLPLNTKNST-KE--P--RLKFGS-KHKSNLKQVETGVAKMNT
sp|O35607|BMPR2_MOUSE AQIYPLPKQQNLPKRPTSLPLNTKNST-KE--P--RLKFGN-KHKSNLKQVETGVAKMNT
tr|G3TEP1|G3TEP1_LOXAF TQIYPLPKQQNLPKRPTSLPLNTKNST-KE--P--RLKFGS-KHKSNLKQVETGVAKMNT
tr|G3W7I5|G3W7I5_SARHA AQIYPLPKQQNLPKRPTSLPLNTKNST-KE--P--RLKFGS-KHKSNLKQVETGVAKMNT
tr|F7AU53|F7AU53_MONDO AQIYPLPKQQNLPKRPTSLPLNTKNST-KE--P--RLKFGS-KHKSNLKQVETGVAKMNT
tr|F6RIN4|F6RIN4_CHICK AQVYPLPKQQNLPKRPTSLPLNTKNST-KE--P--RLKFGG-KHKSNLKQVETGVAKMNT
tr|U3IKP1|U3IKP1_ANAPL AQVYPLPKQQNLPKRPTSLPLNTKNST-KE--P--RLKFGG-KHKSNLKQVETGVAKMNT
tr|R4GD98|R4GD98_ANOCA AQVYPLPKQQNLPKRPTTLPLNTKNST-KE--P--RLKFGG-KHKSNLKQVETGVAKMNT
tr|M7AHB0|M7AHB0_CHEMY TQVYPLPKQQNLPKRPTSLPLNPKNST-KE--P--RLKFGG-KHKSNLKQVETGVAKMNT
tr|F6Q787|F6Q787_XENTR AQLYPLPKQQNLPKRPTSLPLNTKNSG-KE--S--RLKFSS-KHKSNLKHVETGVAKMNT
tr|H3B5H3|H3B5H3_LATCH VQVYPLPKQQNLPKRPTSLPLNTKNAT-KE--S--RLKFG--KHKSNLKQVETGVAKMNT
tr|W5MVP1|W5MVP1_LEPOC --MFPLPKQQNLPKRPTSLPLNTKNPTNKE--S--RLKFGS-KHKSNLKQVETGVAKLNT
tr|M4AH18|M4AH18_XIPMA TAIFPLPKQQNLPKRPSSLPLRNKPAKKESSSSSLRFKFGR-SGKSNLRQVEGTKMNIGV
tr|W5UFT6|W5UFT6_ICTPU PTIFPLPKQQNLPKRPTSLALGSKNPSKEPSSS--RLKFG--RHKSNLKQVETGVAKMNV
tr|F1R413|F1R413_DANRE TAMFPLPKQQNLPKRPTSLPLNTKNPGRESSSS--RLKFG--KHKSNLKQVETGVAKMNT
tr|V9KBW6|V9KBW6_CALMI AHLYPLPKQQNLPKRPTSLPLGP-----KE--P--RLKFGSSKHKSNLKQVETGVAKLNA
D-motif
(JIP1-type)
BMPR2
TM
Extracell.
Protein kinase domain
D
D
D-motif
(JIP1-type)
sp|Q13873|BMPR2_HUMAN SKPRRAQRPNSLDLSATNVLDGSSIQI-GESTQDGKSGSGEKIKKRVKTPYSLKRWRPST
sp|O35607|BMPR2_MOUSE SKPRRAQRPNSLDLSATNILDGSSIQI-GESTQDGKSGSGEKIKRRVKTPYSLKRWRPST
tr|G3TEP1|G3TEP1_LOXAF SKPRRAQRPNSLDLSATNILDGSSMQL-GESTQDGKSGSGEKIKKRVKTPYSLKRWRPST
tr|G3W7I5|G3W7I5_SARHA SKPRRAQRPNSLDLSATNALDSTGMQL-GESSQDGKPGSGEKIKKRVKTPYSLKRWRPST
tr|F7AU53|F7AU53_MONDO PKPRRAQRPNSLDLSATNALDGGALQL-GESSQDGKPGSGEKIKKRVKTPYSLKRWRPST
tr|F6RIN4|F6RIN4_CHICK TQTRRAQRPNSLDLSATNSLDSSSMQL-GDSSQDGKSGSGEKIKKRVKTPYSLKRWRPST
tr|U3IKP1|U3IKP1_ANAPL TQSRRAQRPNSLDLSATNSLDSSSMQL-GDSSQDGKSGSGEKIKKRVKTPYSLKRWRPST
tr|R4GD98|R4GD98_ANOCA TRTKRAQRPNSLDLSATNILDSCSMQL-SESSLDSKPGSGEKIKKRVKTPYSLKRWRPST
tr|M7AHB0|M7AHB0_CHEMY TQTRRAQRPNSLDLSATNTLDSSSLQL-GDSSQDGKPGSGEKIKKRVKTPYSLKRWRPST
tr|F6Q787|F6Q787_XENTR RQTRRAQRPNSLDLSNSSIMDNCSTQCLGETLSDSRSGSGEKIKKRVKTPYSLKRWRPST
tr|H3B5H3|H3B5H3_LATCH QQGRRVERPTSLDLSAMNSLDSYSMQC--ETTQDSKPGSGEKIKKRVKTPYSLKRWRPST
tr|W5MVP1|W5MVP1_LEPOC PKARRAERPNSLDLSAASTLDCSSMQS--ENARDCKGGSGEKIKKRVKTPYSLKRWRPST
tr|M4AH18|M4AH18_XIPMA PRARRPERPNSLDLSFTTQDLTSL--GEGLVRPDGTLGTGEKIKKRVKTPYSLKKWRPTT
tr|W5UFT6|W5UFT6_ICTPU DKPRRPERPNSLDLSTTAQDTSVS---GESSSQEGKAASAEKIKRRVKTPYSLKRWRPST
tr|F1R413|F1R413_DANRE PKPRRPERPNSLDLSASSQNSSLL---GEAVSQEGKAGSAEKIKKRVKTPYSLKRWRPST
tr|V9KBW6|V9KBW6_CALMI HQPRRAQRPNSLDLSSASTPDGCTLQC-ETAARESKPAPGDKIKKRVKTPYSLKRWRPST
Novel vertebrate D-motif
B
Ancestral (metazoan) module
D-motif
(NFAT4-type)
Target
sites
Human
ATF2 20 SDDKPFLCTAPGCGQRFTNEDHLAVHKHKHEMTLKFGPARNDSVIVADQTPTPTRFLKNCE 80
ATF7 2 GDDRPFVCNAPGCGQRFTNEDHLAVHKHKHEMTLKFGPARTDSVIIADQTPTPTRFLKNCE 62
CREB5 11 EQERPFVCSAPGCSQRFPTEDHLMIHRHKHEMTLKFPSIKTDN-MLSDQTPTPTRFLKNCE 70
Zn-finger domain
Transactivator region
DNA binding &
dimerization
ATF2
Zn-finger
D
T
D
Leucine zipper
?
D-motif
(JIP1-type)
Human
ATF2 160 TSAIVRPASLQVPNVLLTSSDSSVIIQQAVPSPTSSTVITQAPSSNRPIVPVPGPFPLLLH 220
ATF7 158 TPTIVRPGSLPLHLGY-------DPLHPTLPSPTS--VITQAPPSNRQMGSPTGSLPLVMH 209
CREB5 103 GGAMTGPGTHQLSSARLPNHDTNVVIQQAMPSPQSSSVITQAPSTNRQIGPVPGSLSSLLH 166
Paralog-specific de novo (vertebrate) module?

Figure S10. Independent emergence of multiple D-motifs in a single protein

(A) The human bone morphogenic protein receptor 2 (BMPR2) possesses two very similar, JIP1-type motifs on its disordered C-terminus (Podkowa *et al*, 2010). Despite the sequence similarity, both have distinct origins. The upstream motif is detected in most multicellular animals including insects. At the same time, the downstream motif appears to be a relatively recent addition found only in vertebrates.
(B) All proteins belonging to the ATF2 family of transcription factors carry a similar, NFAT4-like docking motif on their N-terminus (partially overlapping with a Zn-finger). This element is known to control phosphorylation of a downstream transactivation motif essential for ATF2 activity (Livingstone *et al*, 1995). In vertebrates, out of the three closely related paralogs (ATF2, ATF7 and CREB5), two (ATF2 and ATF7) have an additional, JIP1-type docking motif to recruit JNK to a different region of the protein (Chen *et al*, 2014).

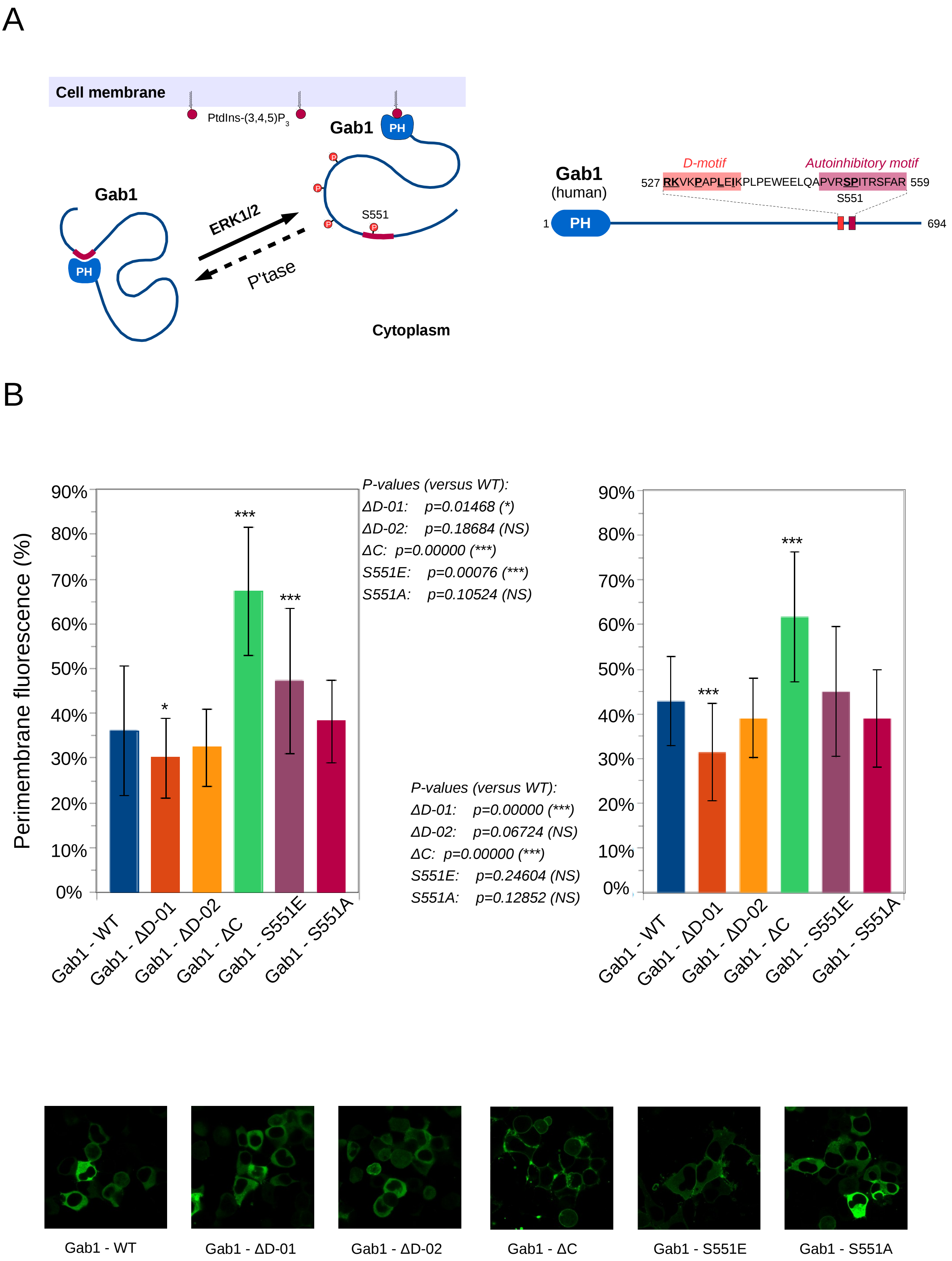
A
Cell membrane
PtdIns-(3,4,5)P3
Gab1
PH
Gab1
PH
ERK1/2
P'tase
S551
P
Cytoplasm
Gab1
(human)
D-motif
Autoinhibitory motif
527 RKVKPAPLEIKPLPEWEELQAPVRSPITRSFAR 559
S551
1
PH
694
B
Perimembrane fluorescence (%)
90%
80%
70%
60%
50%
40%
30%
20%
10%
0%
*
***
***
P-values (versus WT):
ΔD-01: p=0.01468 (*)
ΔD-02: p=0.18684 (NS)
ΔC: p=0.00000 (***)
S551E: p=0.00076 (***)
S551A: p=0.10524 (NS)
Gab1 - WT
Gab1 - ΔD-01
Gab1 - ΔD-02
Gab1 - ΔC
Gab1 - S551E
Gab1 - S551A
P-values (versus WT):
ΔD-01: p=0.00000 (***)
ΔD-02: p=0.06724 (NS)
ΔC: p=0.00000 (***)
S551E: p=0.24604 (NS)
S551A: p=0.12852 (NS)
Gab1 - WT
Gab1 - ΔD-01
Gab1 - ΔD-02
Gab1 - ΔC
Gab1 - S551E
Gab1 - S551A

C

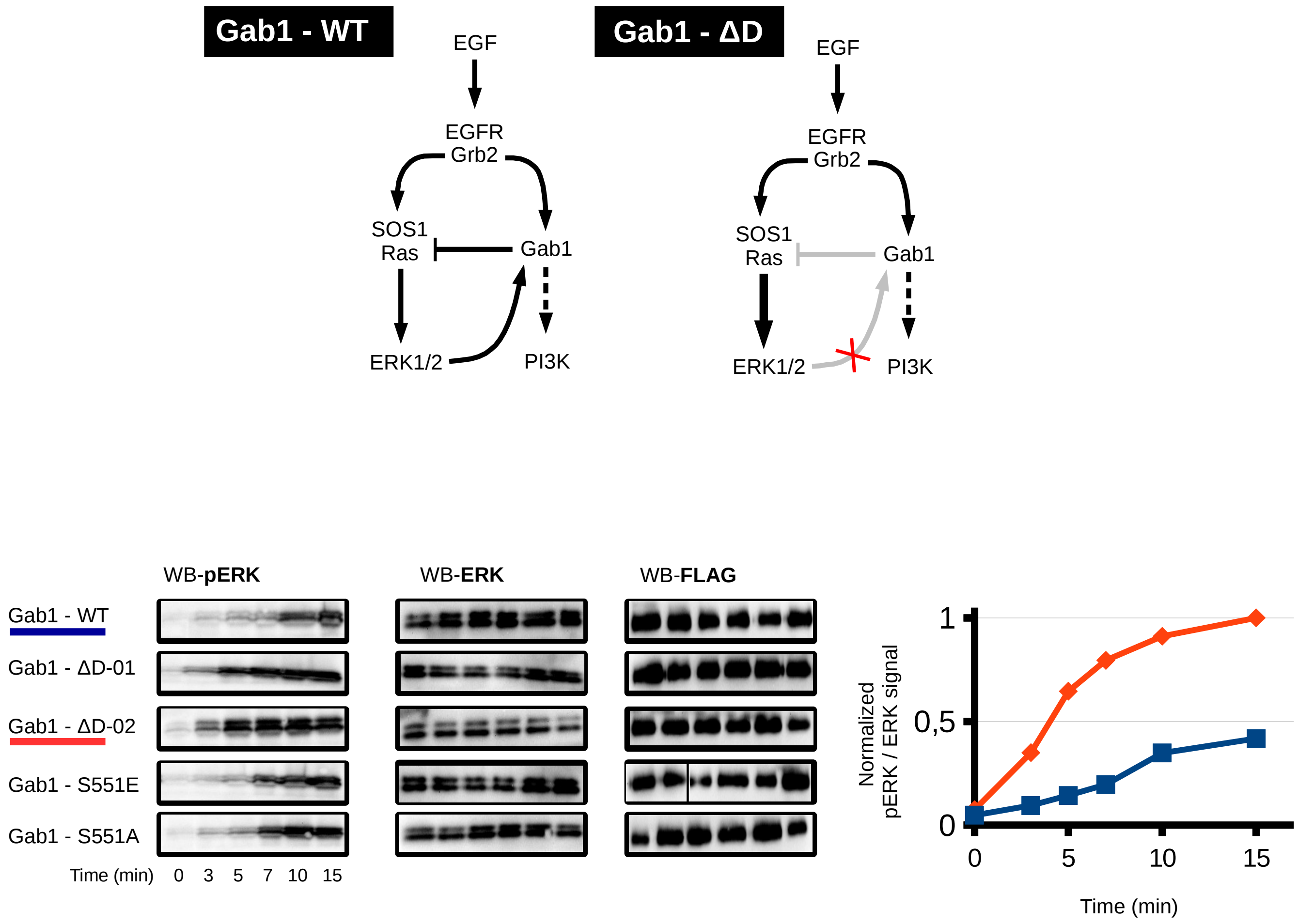

Figure S11. Role of a GAB1 D-motif in the context of the EGF/Ras→ERK signaling cascade

(A) GAB1 contains an N-terminal PH domain and a C-terminally located intra-molecular switch controlling the PH domain's capacity to bind to the cell membrane. This autoinhibition can be relieved by phosphorylation on Ser551.
(**B**) Subcellular localization of CFP-GAB1 constructs expressed in HEK293T cells in serum-containing medium (10% FBS, left) or under serum-free conditions after 10 minutes of EGF stimulation (right). Fraction of all fluorescence within a 2μM range from the cell membrane versus the total fluorescence of the cell are shown on the plot (N=50 cells from all wells). Sample images of cells (obtained through confocal laser scanning microscopy, in 40x magnification) are shown below. The isolated PH domain is predominantly at the cell membrane, as is, to a lesser extent, the Gab1-S551E mutant. In contrast Gab1-S551A does not show elevated perimembrane localization. More importantly, GAB1 docking mutants have a tendency to be located in the cytoplasm under both conditions. (ΔD-01: L534E mutant, where the pivotal φA hydrophopic contact is ablated in the Far1-type 527-RKVKPAPLEI-536 D-motif; ΔD-02: R527 and K528 were mutated to alanine to interfere with electrostatic interactions in the CD groove; ΔC: 1-318 GAB1 construct containing an unmasked PH domain; S551E: Ser551 was replaced by phosphorylation mimicking glutamate residue; S551A: Ser551 was replaced to alanine.) P-values are from Mann-Whitney U-test (*: $p< 0.05$; ***: $p< 0.001$; NS: not significant).
(C) Time course of ERK1/2 phosphorylation in EGF-stimulated, GAB1-transfected HEK293T cells. The marked enhancement of ERK1/2 signaling in the GAB1 docking mutants versus the wild-type GAB1 can be explained by the existence of negative cross-feeding circuits driven by ERK1/2-dependent phosphorylation of GAB1 (such as membrane-bound GAB1 recruiting RasGAP1 to inhibit Ras) (Montagner *et al*, 2005). Note that GAB1 is phosphorylated on multiple sites by ERK1/2, not only affecting its localization, but its signaling properties as well (Lehr *et al*, 2004). WB-Western blot; WB-pERK: phosphoERK; WB-ERK: total ERK; WB-FLAG was used to show equal amount of GAB1 expression levels as the transiently expressed CFP-GAB1 construct was FLAG-tagged.

Table S1: FoldX templates used per motif subtypes

Motif classes and subclasses, consensus motif definitions and the structural templates (with Protein Data Bank identifiers) are shown in a tabulated format.

Name of motif class (or subtype)		Consensus sequence*	Remarks[#]	PDB ID of the structural template[&]
JIP1 class		θ-φ_L-x_1-x_2-φ_A-x_3-φ_B	φ_L = P<br>φ_A = L[$]<br>$x_1$, x_2 = [^P]	1UKH (JNK1)
NFAT4 class		θ-x_1-x_2-φ_L-x_3-φ_A-x_4-φ_B	φ_L = [LIM][$]<br>$\varphi_A$ = L[$]<br>$x_1$, x_2 = [^P]	2XS0 (JNK1)
greater MEF2A class	MEF2A type	θ-θ-x_1-x_2-φ_L-x_3-φ_A-x_4-φ_B	-	1LEW[a] (p38α)
	MKK6 type	θ-x_1-θ-x{3}-φ_L-x_5-φ_A-x_6-φ_B	-	2Y8O[a] (p38α)
	other types	θ{1-2}-x{2-4}-φ_L-x_5-φ_A-x_6-φ_B	-	N/A
greater DCC class	DCC type	θ{1-2}-x{2-4}-φ_L-x_5-x_6-φ_A-x_7-φ_B	φ_L= [PLIV]<br>x_5=P[$]	3O71[a] (ERK2)
	Far1 type	θ{1-2}-x{2-4}-φ_L-x_5-x_6-φ_A-x_7-φ_B	φ_L= [PLIV]<br>x_6=P[$]	3O71[ab] (ERK2)
greater HePTP class	Ste7 type	φ_U-x_1-θ-θ-x{4}-φ_L-x_6-φ_A-x_7-φ_B	x_1 = [^P]	2B9I[c] (FUS3)
	HePTP type	φ_U-x_1-x_2-θ-θ-x_3-x{4}-φ_L-x_8-φ_A-x_9-φ_B	x_1, x_2 = [^P]<br>x_3=[G]	2GPH[d] (ERK2)

* By default, θ: [RK], φ_U:[LIV], φ_L: [LIVMP], φ_A: [LIV], φ_B: [LIVMF]; x can be any amino acid unless defined otherwise in the following column. Numbers in brackets indicate intervening region length. (Initial searches used more liberal amino acid definitions per core positions, also allowing Pro in the φ_B position)

[#] For x, numbering is from left to right in the consensus; ^ means exclusion of an amino acid like proline for example [^P]. For some consensus patterns further amino acid requirements were specified based on known structures of MAPK–docking motif complexes (e.g. x_2 = [^P] in the NFAT4 motif class indicates that the second intervening region position from the start of the motif, from left to right, may not be a helical conformation breaking proline.)

[$] These additional constraints were introduced only after the conclusion of experimental testing & evolutionary conservation analyses

[&] Name of the MAPK in the structural model is shown in brackets.

[a] The N-terminal regions with all θ positions (which were flexible in the crystal structures) were not used for FoldX-based scoring

[b] The human ERK2-DCC complex was used for energy estimations in all greater DCC class motifs. The yeast FUS3-Far1 model (PDB ID: 2B9J) only served as a theoretical basis to establish subtype splitting between DCC and Far1 types. (The proline-rich segment between φ_L and φ_A has nearly identical conformation in models 3O71 and 2B9J. It differs only in the exact placement of proline amino acids. Thus homology modeling based on the yeast 2B9J complex was unnecessary.)

[c] The FUS3-Msg5 complex served as a model for this class (instead of the incomplete FUS3-Ste7 complex that was crystallized with an N-terminally truncated peptide [PDB ID: 2B9H]). The yeast FUS3 MAPK was subsequently exchanged for human ERK2 and the resulting theoretic structure was optimized with PepFlexDock.

[d] The artificial disulfide bridge (formed as a crystallization artifact in this complex) was removed. Afterwards, the peptide chain was remodeled with FlexPepDock.

Appendix References

Bayer M, Fischer J, Kremerskothen J, Ossendorf E, Matanis T, Konczal M, Weide T & Barnekow A (2005) Identification and characterization of Iporin as a novel interaction partner for rab1. *BMC Cell Biol.* **6:** 15

Bogoyevitch MA, Yeap YYC, Qu Z, Ngoei KR, Yip YY, Zhao TT, Heng JI & Ng DCH (2012) WD40-repeat protein 62 is a JNK-phosphorylated spindle pole protein required for spindle maintenance and timely mitotic progression. *J. Cell Sci.* **125:** 5096–5109

Cai Q, Pan P-Y & Sheng Z-H (2007) Syntabulin-kinesin-1 family member 5B-mediated axonal transport contributes to activity-dependent presynaptic assembly. *J. Neurosci.* **27:** 7284–96

Cohen-Katsenelson K, Wasserman T, Khateb S, Whitmarsh AJ & Aronheim A (2011) Docking interactions of the JNK scaffold protein WDR62. *Biochem. J.* **439:** 381–90

Deller T, Korte M, Chabanis S, Drakew A, Schwegler H, Stefani GG, Zuniga A, Schwarz K, Bonhoeffer T, Zeller R, Frotscher M & Mundel P (2003) Synaptopodin-deficient mice lack a spine apparatus and show deficits in synaptic plasticity. *Proc. Natl. Acad. Sci. U. S. A.* **100:** 10494–9

Gdalyahu A, Ghosh I, Levy T, Sapir T, Sapoznik S, Fishler Y, Azoulai D & Reiner O (2004) DCX, a new mediator of the JNK pathway. *EMBO J.* **23:** 823–32

Heydet D, Chen LX, Larter CZ, Inglis C, Silverman MA, Farrell GC & Leroux MR (2013) A truncating mutation of Alms1 reduces the number of hypothalamic neuronal cilia in obese mice. *Dev. Neurobiol.* **73:** 1–13

Kim JG, Armstrong RC, v Agoston D, Robinsky A, Wiese C, Nagle J & Hudson LD (1997) Myelin transcription factor 1 (Myt1) of the oligodendrocyte lineage, along with a closely related CCHC zinc finger, is expressed in developing neurons in the mammalian central nervous system. *J. Neurosci. Res.* **50:** 272–90

Kim MO, Kim S-H, Cho Y-Y, Nadas J, Jeong C-H, Yao K, Kim DJ, Yu D-H, Keum Y-S, Lee K-Y, Huang Z, Bode AM & Dong Z (2012) ERK1 and ERK2 regulate embryonic stem cell self-renewal through phosphorylation of Klf4. *Nat. Struct. Mol. Biol.* **19:** 283–90

Koyano S, Ito M, Takamatsu N, Shiba T, Yamamoto K & Yoshioka K (1999) A novel Jun N-terminal kinase (JNK)-binding protein that enhances the activation of JNK by MEK kinase 1 and TGF-beta-activated kinase 1. *FEBS Lett.* **457:** 385–8

Lamba DA, Hayes S, Karl MO & Reh T (2008) Baf60c is a component of the neural progenitor-specific BAF complex in developing retina. *Dev. Dyn.* **237:** 3016–23

Lehr S, Kotzka J, Avci H, Sickmann A, Meyer HE, Herkner A & Muller-Wieland D (2004) Identification of major ERK-related phosphorylation sites in Gab1. *Biochemistry* **43:** 12133–40

Lu QR, Cai L, Rowitch D, Cepko CL & Stiles CD (2001) Ectopic expression of Olig1 promotes oligodendrocyte formation and reduces neuronal survival in developing mouse cortex. *Nat. Neurosci.* **4:** 973–4

Mihaylova MM & Shaw RJ (2011) The AMPK signalling pathway coordinates cell growth, autophagy and metabolism. *Nat. Cell Biol.* **13:** 1016–23

Montagner A, Yart A, Dance M, Perret B, Salles J-P & Raynal P (2005) A novel role for Gab1 and SHP2 in epidermal growth factor-induced Ras activation. *J. Biol. Chem.* **280:** 5350–60

Okamoto M & Südhof TC (1997) Mints, Munc18-interacting proteins in synaptic vesicle exocytosis. *J. Biol. Chem.* **272:** 31459–64

Shen C-H, Yuan P, Perez-Lorenzo R, Zhang Y, Lee SX, Ou Y, Asara JM, Cantley LC & Zheng B (2013) Phosphorylation of BRAF by AMPK impairs BRAF-KSR1 association and cell proliferation. *Mol. Cell* **52:** 161–72

Simon-Areces J, Dopazo A, Dettenhofer M, Rodriguez-Tebar A, Garcia-Segura LM & Arevalo M-A (2011) Formin1 mediates the induction of dendritogenesis and synaptogenesis by neurogenin3 in mouse hippocampal neurons. *PLoS One* **6:** e21825

Sun T, Yu N, Zhai L-K, Li N, Zhang C, Zhou L, Huang Z, Jiang X-Y, Shen Y & Chen Z-Y (2013) c-Jun NH2-terminal Kinase (JNK)-interacting Protein-3 (JIP3) Regulates Neuronal Axon Elongation in a Kinesin- and JNK-dependent Manner. *J. Biol. Chem.* **288:** 14531–14543

Watabe-Uchida M, John KA, Janas JA, Newey SE & Van Aelst L (2006) The Rac activator DOCK7 regulates neuronal polarity through local phosphorylation of stathmin/Op18. *Neuron* **51:** 727–39

Yau KW, van Beuningen SFB, Cunha-Ferreira I, Cloin BMC, van Battum EY, Will L, Schätzle P, Tas RP, van Krugten J, Katrukha EA, Jiang K, Wulf PS, Mikhaylova M, Harterink M, Pasterkamp RJ, Akhmanova A, Kapitein LC & Hoogenraad CC (2014) Microtubule minus-end binding protein CAMSAP2 controls axon specification and dendrite development. *Neuron* **82:** 1058–73
