# Supplementary material for: Systematic discovery of linear binding motifs targeting an ancient protein interaction surface on MAP kinases
Source: Mol Syst Biol. 2015 Nov 4;11(11):837. doi: 10.15252/msb.20156269 (PMC4670726; doi:10.15252/msb.20156269)
Supplement: Supplementary file 2 — Expanded View Figures PDF [file MSB-11-837-s002.pdf]

Expanded View Figures

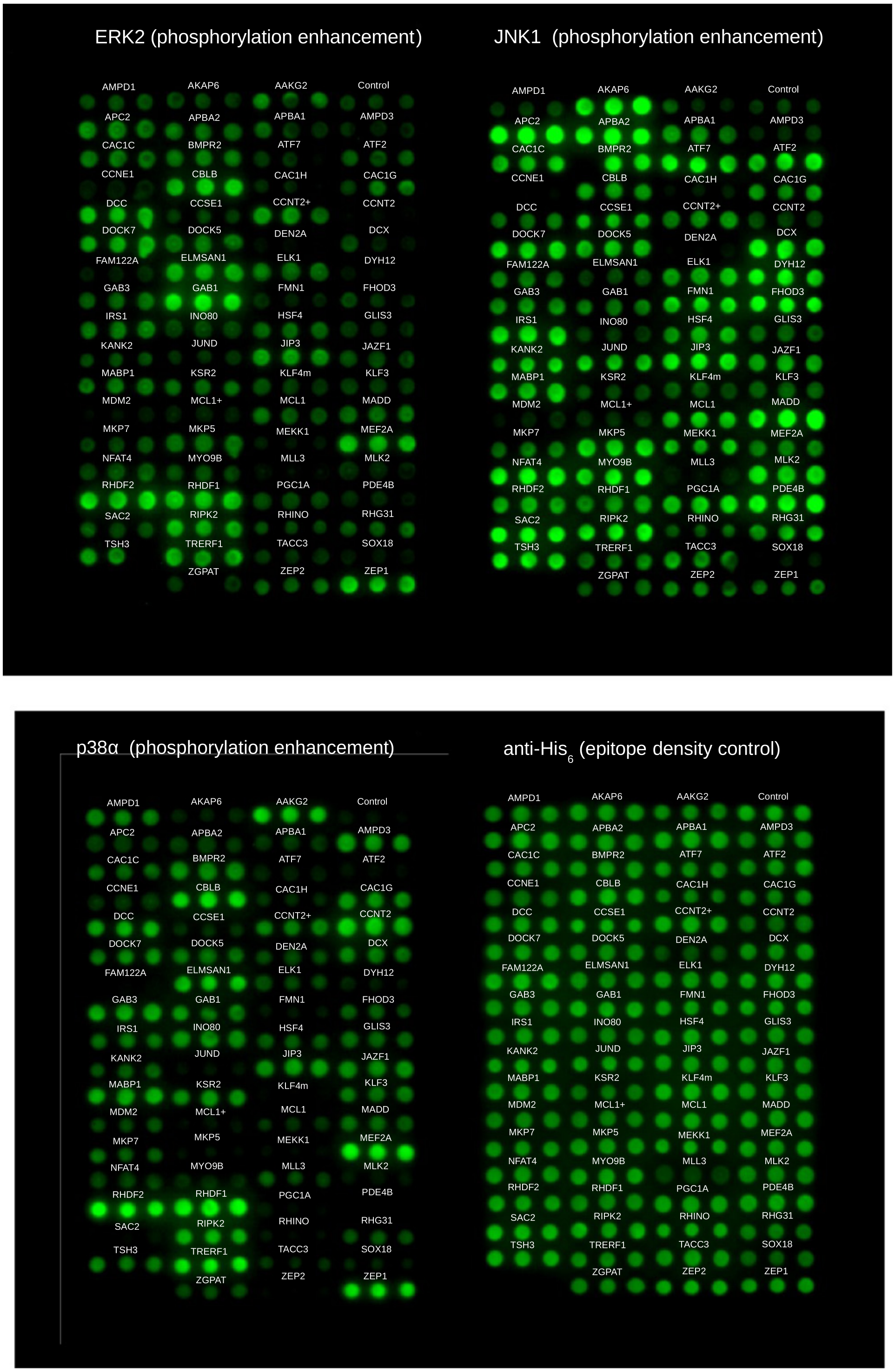

Figure EV1. Results of dot-blot experiments.
Panels show dot blots of all 71 constructs (70 D-motif-containing substrates + 1 negative control) against three MAPKs and the epitope density control (see also Appendix Fig S1).

 © 2015 The Authors

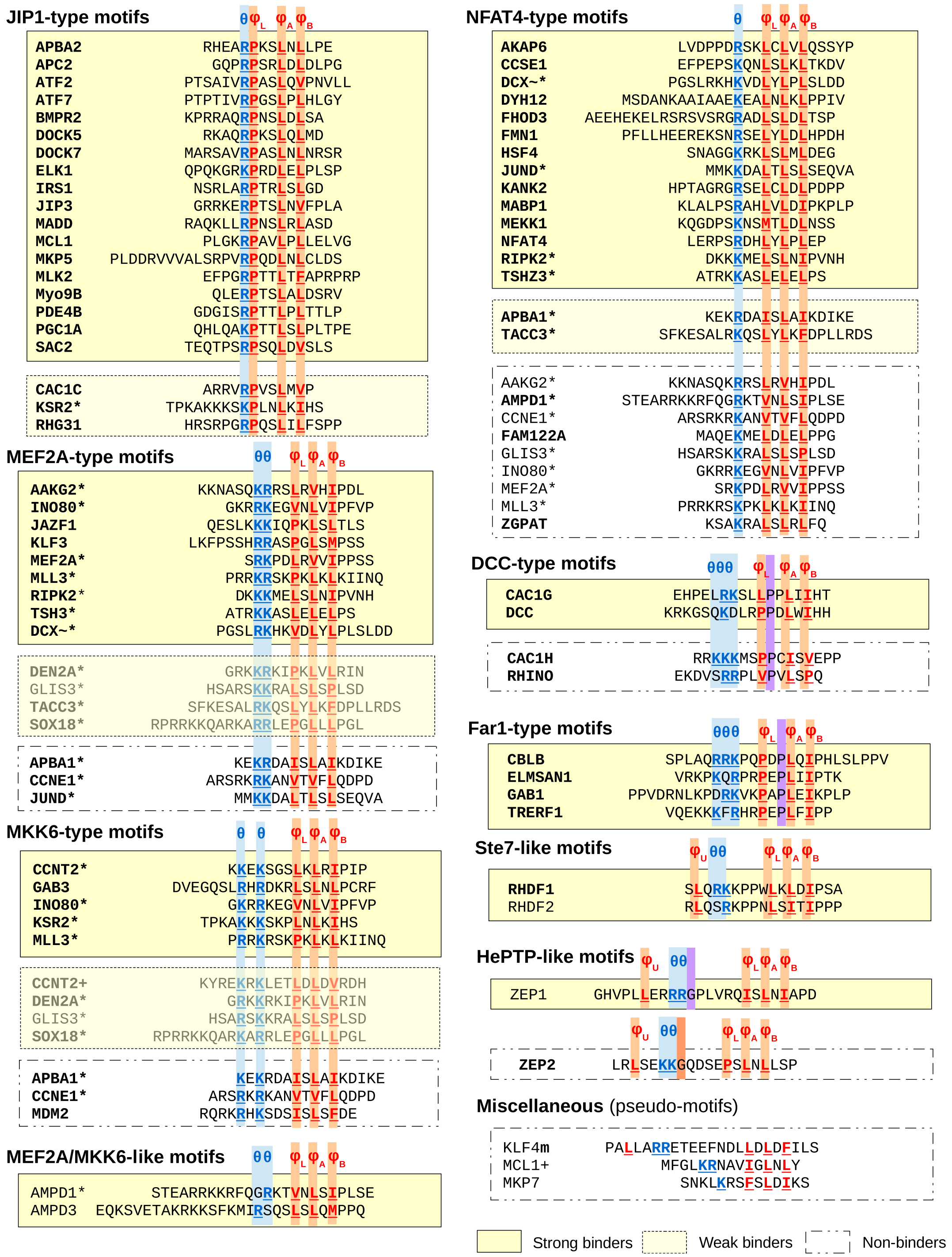

Figure EV2. Constructs used in dot-blot experiments and grouped according to motif class.
Amino acid sequences of D-motif-containing short segments ligated into the pAZAD vector are displayed in a tabulated format. Tested sequences are grouped according to D-motif types. In addition, the conformity of the motif to a loose or tight motif definition (see Appendix Fig S2) is also indicated: Motifs whose sequence fits to its tight D-motif class definition is written in bold type, while those that only correspond to a looser definition is shown in normal type. The outcome of dot-blot experiments is also indicated: A motif is regarded as a "strong binder" (unequivocally positive) if it performed above the cutoff (non-cognate D-motif control) in all experiments. In case if it performed below this cutoff in at least one round of experiments, but still consistently above the level of zero control (substrate with no D-motif), it is considered a "weak binder". Otherwise, the motif is classified as a "non-binder". Only "strong binders" were considered as "positives" for the purpose of sequence and evolutionary analysis. +: These proteins had more than one, non-overlapping MAPK-binding elements tested (names with/without "+" refer to different sequences). *: The sequence of these constructs satisfies at least two different, overlapping consensus motifs (thus, they are featured under more than one motif class/subclass). For the sake of simplicity, not all combinations are shown. ~: This novel isoform is found in NCBI and other databases but not in UniProt. m: This sequence refers to the murine instead of the human protein (Kim et al, 2012).

Protein name	Peptide sequence	Kd values (μM) ERK2	JNK1	p38α	
APBA2	RHEARPKSLNLL	–	10.8	–	JIP1 class
ATF7~	EEPTIVRPGSLPLHLGYE	–	21.1	–	JIP1 class
DOCK5	KAQRPKSLQLM	31.8	5.1	–	JIP1 class
DOCK7	RSAVRPASLNLNRSR	51.2	7.3	–	JIP1 class
IRS1	RLARPTRLSL	–	3.5	74.6	JIP1 class
MYO9B	LERPTSLALD	–	15.9	–	JIP1 class
MKP5	EESRPVRPQDLNLSLDSE	84.7	19.3	–	JIP1 class
PDE4B	EGDGISRPTTLPLTTLPS	–	16.4	–	JIP1 class
DCX	SLRKHKVDLYLPLSL	59.0	16.7	29.5	NFAT4 class
AAKG2	SQKRRSLRVHIP	17.5	–	8.2	greater MEF2A class
CCNT2	KKEKSGSLKLRIPIP	14.4	–	24.5	greater MEF2A class
GAB3	SLRHRDKRLSLNLPSR	22.6	14.5	5.2	greater MEF2A class
JAZF1	SLKKKIQPKLSLTLS	15.0	–	8.9	greater MEF2A class
KSR2	KKKSKPLNLKI	13.5	24.7	4.9	greater MEF2A class
GAB3+	KPQRKSRPPPLDLRNLS	0.7	13.2	2.0	greater DCC class
RHDF1	SLQRKKPPWLKLDIPS	0.9	–	1.2	greater HePTP class

Figure EV3. Protein–peptide binding affinity assays.
The results of fluorescence polarization (FP) titrations are displayed in a tabulated format, showing the sequence of synthetic peptide used together with the dissociation constants (Kd) obtained for each MAPK (see also Appendix Fig S3). Dashes indicate cases where the curves could not be fitted (the Kd is above the limit of quantitation of this assay, approximately ~100 μM). Peptides are grouped in two clusters, the first being the strong JNK1 binders (frequently with little or no ERK2/p38α-binding ability). The second group (below the magenta line) contains peptides with strong ERK2- and p38α-binding capacity (but often little or no affinity toward JNK1). Coloring of the peptide residues displays the critical charged θ residues in blue and the φ hydrophobic positions in red (according to the best fitting structural models). ~: The ATF7 peptide has non-native flanking amino acids (several glutamic acids were added onto both its N- and C-terminal ends) in order to make the peptide soluble near neutral pH. +: Motif not included in the dot-blot screens. Gab3+ is the ortholog of a peptide from Gab1 which was tested as positive in the dot-blot experiments. The two Gab3 motifs are related neither by structure nor by evolutionary origin.

Figure EV4. Coevolution of MAPK-docking motifs and phosphorylation target sites.

A In the case of the NFAT family of transcription factors, phosphorylation of an N-terminal regulatory motif (the so-called serine-rich region 1/SRR1) serves to retain the protein in the cytoplasm, denying nuclear entry. While processive phosphorylation of the SRR1 motif is performed by casein kinase I (CK1) in all four vertebrate paralogs, priming of phosphorylation can be provided by diverse other protein kinases. In the case of NFAT4 (also known as NFATC3), a JNK-specific docking motif ensures that CK1 priming can be driven by JNK kinases (Chow *et al*, 1997). Comparison with other NFAT paralogs suggests that this mechanism in NFAT4 is fairly novel, adding on to a previously evolved regulatory system. (Some elements of the SRR1 motif can already be recognized in non-vertebrate orthologs).

B The docking motif encountered in the human myocyte enhancement factor 2 A (MEF2A) protein is ancient: Transcription factors belonging to the MEF2/MADS-box family carry docking motifs in almost all multicellular animals (and even in some unicellular eukaryotes, fungi, or plants) (Jung *et al*, 2002; Yang *et al*, 1999). The D-motif controls phosphorylation of a conserved motif indispensable for transactivation. However, this ancestral module is preserved in two of the four vertebrate paralogs only (MEF2A and MEF2C). Other vertebrate paralogs show either a partial loss of the docking motif (MEF2D) or a complete loss of the docking motif together with the transactivator motif (MEF2B). The latter is probably an evidence of neofunctionalization, with the divergent MEF2B no longer being involved in generic mesoderm and myocyte development, taking up other specialized roles (such as lymphocyte regulation) instead (Ying *et al*, 2013; Estrella *et al*, 2015).

C The Far1-type MAPK-docking motif identified by our experiments in GAB1 has homologs in 3 out of the 4 human GAB proteins. It is located in the vicinity of a well-known ERK2 phosphorylation site that controls an intramolecular interaction. Residues surrounding this C-terminal site bind to the N-terminal plecstrin homology (PH) domain preventing membrane recruitment of GAB proteins (Eulenfeld & Schaper, 2009). Phosphorylation by ERK2 disrupts the intramolecular interaction allowing GAB1 translocation (Wolf *et al*, 2015). Although testing this hypothesis requires further experiments, it is probable that the ERK2-docking motif and the ERK2 phosphorylation site form a single functional module. The evolutionary analysis suggests that both sites emerged prior to the development of vertebrates, at roughly the same time (the entire module is found in a single *Branchiostoma floridae* protein, but not in other invertebrates). While GAB1 and GAB2 retained the ancestral architecture, GAB4 lost both the docking motif and the phosphorylation target site. GAB3 did lose the phosphorylation target site, yet the D-motif was retained. However, emergence of an additional, MEF2A-type motif in GAB3 further away from the original one suggests that these motifs might now control different target site(s) in GAB3. Data information: *, Match between the regions is insecure due to poor conservation; **, the sequence is so divergent from the other vertebrate paralogs that the remnants of the original MAPK-regulated region could only be matched to the rest with the help of intron–exon architecture (MEF2B); ***, The exact branching pattern is uncertain, due to high divergence of certain paralogs (MEF2B or GAB3).

© 2015 The Authors

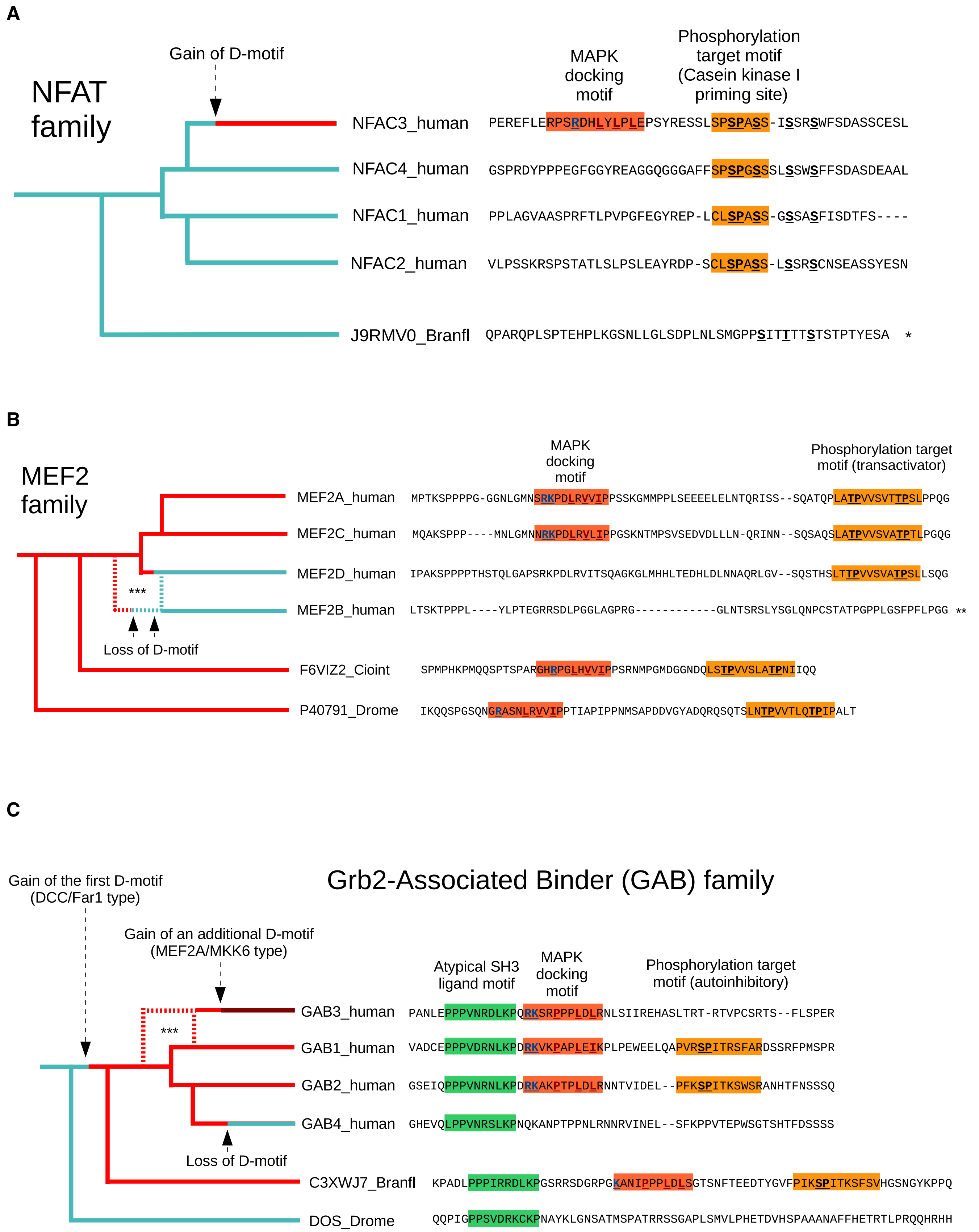

Figure EV4.

© 2015 The Authors

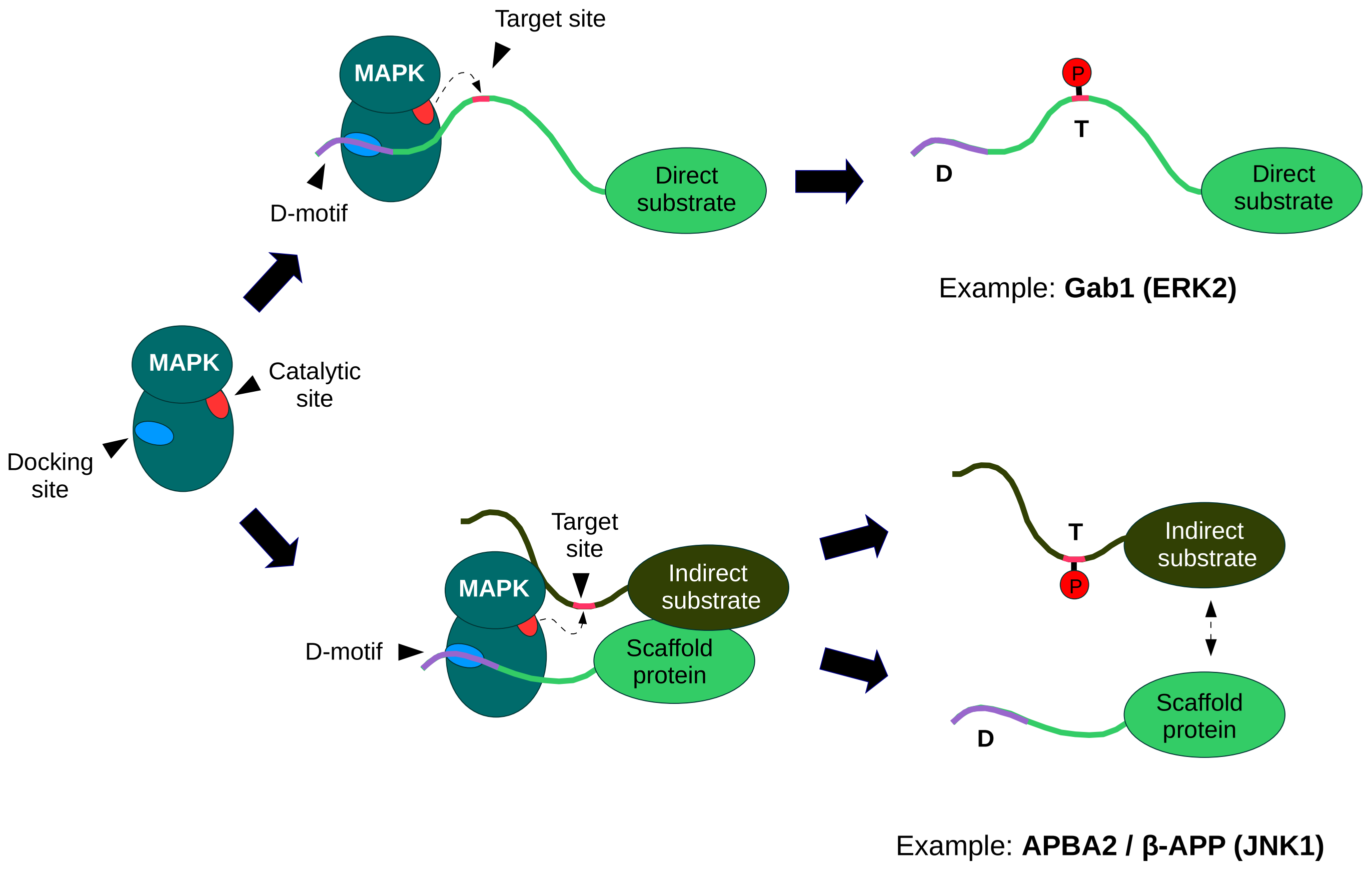

Figure EV5. Illustration of direct and indirect MAPK substrates.
Most of the D-motif-containing proteins likely fall into two categories. In proteins that are direct MAPK substrates (upper diagram), the docking motif might serve to enhance phosphorylation of regulatory sites. This is exemplified by a known substrate, GAB1 (with the target motif being only recently identified) (Wolf *et al*, 2015). Other proteins can act as scaffolds to aid phosphorylation of substrates without docking motifs (here termed indirect MAPK substrates, lower diagram). For example, the requirement for APBA2 to mediate JNK-dependent phosphorylation of beta-amyloid precursor protein (β-APP) has already been described and the minimally required fragment of APBA2 contains the JIP1-type docking motif-containing region as well as a PTB domain (Taru & Suzuki, 2004).

 © 2015 The Authors
